# Design, Synthesis, and Nematocidal Evaluation of Waltherione A Derivatives: Leveraging a Structural Simplification Strategy

**DOI:** 10.3390/ijms25179209

**Published:** 2024-08-25

**Authors:** Zhan Hu, Bin Yang, Shuai Zheng, Ke Zhao, Kaifeng Wang, Ranfeng Sun

**Affiliations:** Key Laboratory of Green Prevention and Control of Tropical Agriculture and Forestry BioDisasters of Ministry of Education, School of Tropical Agriculture and Forestry, Hainan University, Haikou 570228, China; huzhan@hainanu.edu.cn (Z.H.); 20095132210152@hainanu.edu.cn (K.Z.);

**Keywords:** designed and synthesized, fungicide, nematicides, quinoline and quinolone derivatives, Waltherione A

## Abstract

Southern root-knot nematodes are among the most pernicious phytoparasites; they are responsible for substantial yield losses in agricultural crops worldwide. The limited availability of nematicides for the prevention and control of plant-parasitic nematodes necessitates the urgent development of novel nematicides. Natural products have always been a key source for the discovery of pesticides. Waltherione A, an alkaloid, exhibits potent nematocidal activity. In this study, we designed and synthesized a series of quinoline and quinolone derivatives from Waltherione A, leveraging a strategy of structural simplification. Bioassays have revealed that the quinoline derivatives exhibit better activity than quinolone derivatives in terms of both nematocidal and fungicidal activities. Notably, compound D1 demonstrated strong nematocidal activity, with a 72 h LC_50_ of 23.06 μg/mL, and it effectively controlled the infection of root-knot nematodes on cucumbers. The structure–activity relationship suggests that the quinoline moiety is essential for the nematocidal efficacy of Waltherione A. Additionally, compound D1 exhibited broad-spectrum fungicidal activity, with an EC_50_ of 2.98 μg/mL against *Botrytis cinerea*. At a concentration of 200 μg/mL, it significantly inhibited the occurrence of *B. cinerea* on tomato fruits, with an inhibitory effect of 96.65%, which is slightly better than the positive control (90.30%).

## 1. Introduction

The southern root-knot nematode (*Meloidogyne incognita*) is a polyphagous, obligate, sedentary endoparasite; it is one of the most notorious phytoparasitic nematodes [1,2]. After the root is invaded by *M. incognita*, it will be stunted and deformed and will sprout many characteristic galls or knots as a result of the *M. incognita* developing many permanent feeding sites in its vascular tissues [3]. Additionally, the parasitized plant will exhibit other typical symptoms, including stunted growth, wilting, and leaf discoloration [4]. *M. incognita* not only has a wide range of hosts (it can be found in almost every plant family) [5] and living areas (it can be found in every country with a minimum temperature above 3 °C) [6], but it also predisposes the plant roots to secondary infections by other microorganisms (such as fungi, bacteria, and viruses) [7,8], making it perhaps the most threatening of all crop pathogens in modern agriculture [9]. According to the survey, *Meloidogyne* spp. causes crop losses of approximately USD 100 billion worldwide per year, and crop losses due to *M. incognita* even exceed 20% in some areas [5,10]. Therefore, it is crucial to reduce the number of phytoparasitic nematodes for maximizing production of the crop.

Until now, nematicides are still one of the most reliable tools for controlling root-knot nematodes, although there are many other useful means such as cultural measures, plant resistance, and biological control. Nevertheless, most widely used nematicides have been banned due to environmental and human health concerns [11]. However, in the past fifty years, there have been no more than ten newly commercialized nematicides [12,13]. Consequently, it is significant important to research and discover new nematicides that are environmentally friendly and have low toxicity [14].

**Figure 1:**
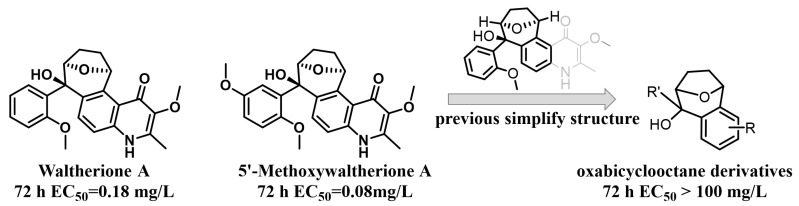
Our previous work.

Given their biologically relevant chemical diversity and high-affinity interactions with their biological targets, natural products have been and continue to be a key arsenal for the discovery of new medicines and pesticides [12,15]. Reports indicate that potentially 17–50% of all pesticides have or could have related to natural products [16] and many natural products show good nematocidal activity [14]. Waltherione A (Figure 1), a quinolone-type alkaloid, was first isolated from *Waltheria douradinha* (Sterculiaceae) in 2005; with its analogue 5′-Methoxywaltherione A (Figure 1) demonstrated strong nematocidal activity against juveniles of *M. arenaria*, *M. hapla*, *M. incognita,* and *Bursaphelenchus xylophilus* [17,18]. The median effective concentration (EC_50/72 h_) values of Waltherione A and 5′-Methoxywaltherione A against the second-stage juveniles (J2) of *M. incognita* were 0.18 μg/mL and 0.08 μg/mL, respectively. The activity is comparable to the positive control avermectin (EC_50/72 h_, 0.11 μg/mL) [18], which is the mainstream nematicide at present [13]. However, like many other plant secondary metabolites, Waltherione A’s potential as a nematicide is constrained by its low natural content in plants [18,19] and the complexity of its large-scale synthesis (involving a quinolone fused with a unique oxabicyclo[3.2.1]octane scaffold) [20]. Functional-oriented synthesis strategies that simplify the structure of natural products to create a synthetically accessible molecule while retaining comparable biological functions may solve this problem [21]. The crop protection industry has seen numerous successes through the application of structure simplification of natural products, such as from eserine to insecticide isoprocarb and from UK-2A to fungicide florylpicoxamid [22].

**Figure 2:**
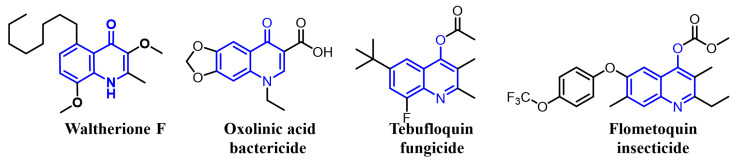
Some representative quinoline structures.

Recently, we have simplified the quinolone moiety in Waltherione A to a benzene ring and synthesized a series of derivatives that maintain the distinctive oxabicyclo[3.2.1]octane framework (Figure 1) [23]. Unfortunately, their nematocidal activity (EC_50/72 h_ > 100 μg/mL) is significantly reduced compared to that of Waltherione A [23]. This result suggests that the quinolone part in Waltherione A is essential for its effectiveness against nematodes. Additionally, quinolone structures, widely used as antibacterial agents in medicine, are also incorporated into the formulas of agricultural fungicides like Oxolinic acid (Figure 2) and Tebufloquin (Figure 2), as well as in the insecticide Flometoquin (Figure 2) [24]. Recent studies indicate that the derivatives of Waltherione F (Figure 2), similar to Waltherione A and also belonging to the quinolone alkaloids, exhibit potent fungicidal activity [25,26]. Based on prior research, we have developed some simplified analogs of Waltherione A, consisting of multiple novel quinolone and quinoline derivatives, and assessed their nematocidal and fungicidal activities both in vitro and in vivo. We aim to explore the structure–activity relationship of Waltherione A to discover new lead compounds with simple structures and potent nematocidal and fungicidal activities.

## 2. Results and Discussion

### 2.1. Chemistry

**Figure 3:**
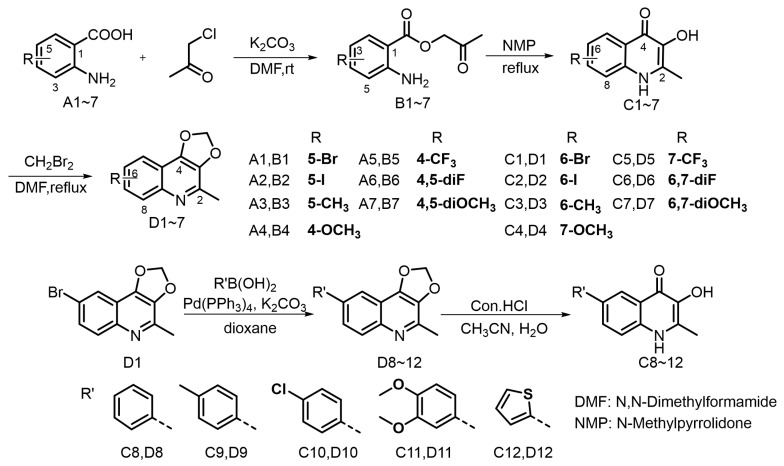
The synthetic route for the target compounds.

Previous studies on the structure–activity relationship of Waltherione A against *M. incognita* indicated the importance of the quinolone structure [23]. Consequently, we designed and synthesized quinolone derivatives to further investigate the relationship; the synthetic route is outlined in Figure 3. The quinolone derivatives were synthesized essentially by the method reported by Horák [27]. Anthranilic acid and chloroacetone underwent esterification reaction to produce intermediate B1~7. Upon heating in NMP, the intermediates transformed into 3-hydroxy-2-methyl-4-quinolinone derivatives C1~7 by a dehydration cyclization rearrangement reaction. It is worth noting that intermediates B can proceed directly to the next reaction without strict purification. However, ensuring their drying can increase the yield of the subsequent dehydration reaction. The 4-quinolinone derivatives have poor solubility in common solvents and readily precipitate. Following filtration, a straightforward washing procedure can obtain a purified product. We initially planned to methylate the hydroxyl group of quinolinone derivatives C1~7, aiming to achieve a structure reminiscent of Waltherione A, but our attempts with iodomethane and dimethyl sulfate did not yield the expected results. This could be attributed to their poor solubility [28]. Fortunately, with K_2_CO_3_ as the base, compound C1~7 can react smoothly with diiodomethane (CH_2_Br_2_) to yield compound D1~7, which exhibit good solubility in common organic solvent. Compound D1 can be effectively converted into compound D8~12 via the Suzuki coupling reaction. Hydrolysis of compound D8~12 with hydrochloric acid can smoothly obtain compound C8~12. The structures of target compounds were confirmed by ^1^H and ^13^C NMR and HRMS. Among the 24 compounds (C1 to D12), compound C3 and C7 are known compounds, whereas all the others are new compounds. The NMR spectrum can be viewed in the Supplementary Materials.

### 2.2. Biology Assays

#### 2.2.1. Nematocidal Activity against *M. incognita* In Vitro and In Vivo

**Figure 4:**
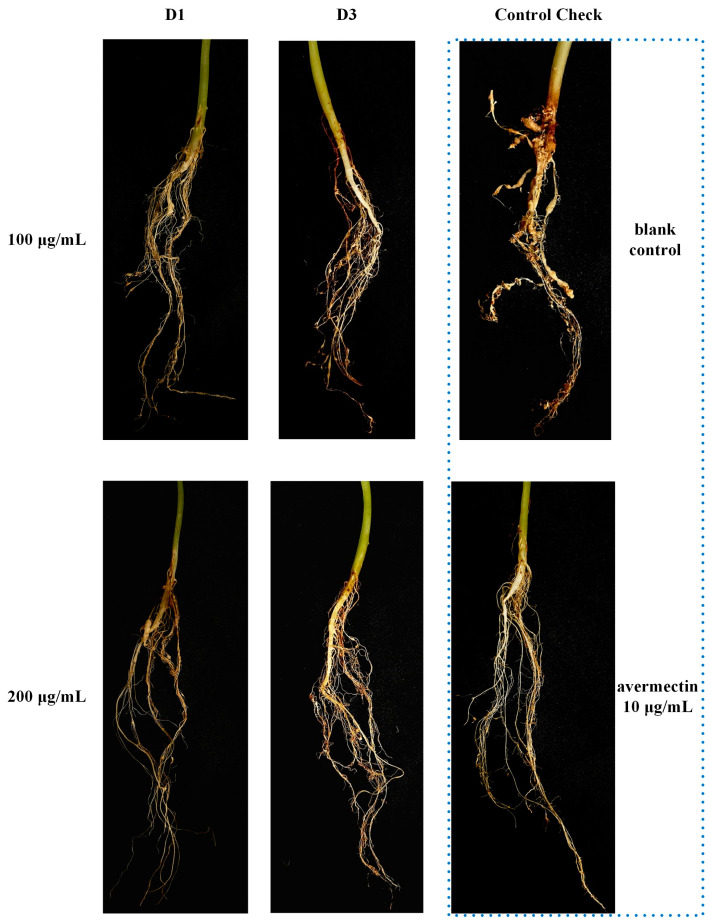
The nematocidal activity in a cucumber pot experiment of Compounds D1, D3, and avermectin.

The lethality against *M. incognita* in vitro was investigated at a concentration of 200 μg/mL, and the results are presented in Table 1. In comparison, the activity of quinoline series compounds (D1~12) is higher than that of quinolone series compounds (C1~12). Compounds D1~3 and D12 showed excellent effect with mortality of 100%. Subsequently, the LC_50_ of highly active compounds was established using tioxazafen and avermectin as positive control, which are shown in Table 2. As the data display, compounds D1 and D3 exhibited good inhibitory effects with LC_50_ values of 23.06 and 30.63 μg/mL, respectively, yet they are slightly less effective than tioxazafen (LC_50_, 17.44 μg/mL) and substantially weaker than avermectin (LC_50_, 0.21 μg/mL). The results of the pot experiment are showed in Table 3 and Figure 4. Compounds D1 and D3 can effectively prevent the formation of root knots on cucumber roots. Specifically, the control effect of Compound D1 (200 μg/mL, 61.57%) was comparable to that of avermectin (10 μg/mL, 64.36%). Compound D1 is a structural simplification of Waltherione A, with the 7-membered oxygen-bridged ring removed. Although its activity (LC_50_ 23.06 μg/mL) is significantly reduced compared to Waltherione A (LC_50_ 0.18 μg/mL), it exhibits much higher activity than the compounds (LC_50_ > 100 μg/mL) obtained from the simplification of the quinolone moiety in the structure of Waltherione A in our previous work. This result indicates that the quinolone structure is crucial for the nematocidal activity of Waltherione A.

#### 2.2.2. Fungicidal Activity In Vitro and In Vivo

**Figure 5:**
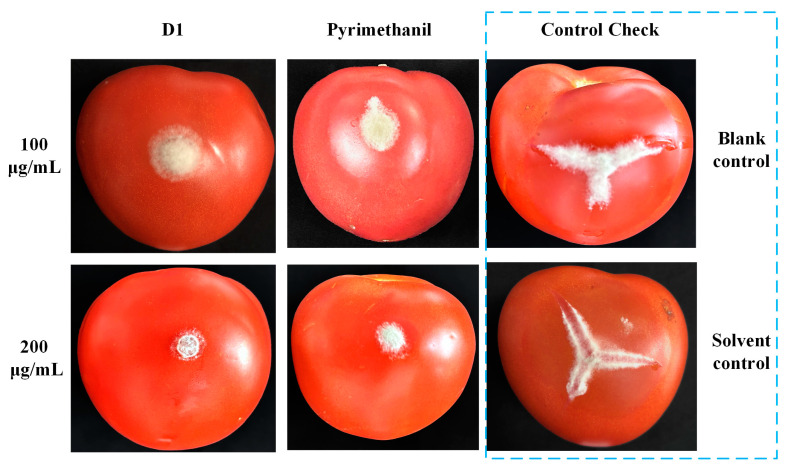
Efficacy of compounds D1 and pyrimethanil on tomato fruit against *B. cinerea* in vivo.

Recently, many studies have highlighted the potent agricultural fungicidal activity of quinoline derivatives [25,26]. In light of this, we evaluated the fungicidal activity of the target compounds against eight prevalent plant pathogens. Initial screenings at a concentration of 50 μg/mL were conducted, and the EC_50_ was also determined for compounds that showed an inhibition rate over 70% in the preliminary screening. Their results are detailed in Table 4 and Table 5. Compounds with a quinolone framework generally exhibited poor fungicidal activity at a concentration of 50 μg/mL. In contrast, many compounds (D1~4, D10~12) with a quinoline structure demonstrated good fungicidal activity. Compound D1, in particular, exhibits remarkable fungicidal activity against a broad spectrum of plant pathogens, achieving an inhibition rate exceeding 70% at a concentration of 50 μg/mL, with EC_50_ values ranging from 2.98 to 29.01 μg/mL. Furthermore, the in vivo activity of compound D1 against *B. cinerea* on tomato fruit was also investigated. As shown in Table 6 and Figure 5, compound D1 has good control effects against pathogens at concentrations of 100 and 200 μg/mL, with inhibition rates of 66.16% and 96.65%, respectively. At higher concentrations, the inhibitory effect of compound D1 is better than the positive control (90.30%). These results indicate that compound D1 is a promising candidate for the management of botrytis rot.

## 3. Materials and Methods

### 3.1. General

All starting materials were purchased from commercial sources like Xilong Chemical Co., Ltd. (Shanghai, China), Innochem (Beijing, China), or Bide Pharmatech Co., Ltd. (Shanghai, China), and were used without further purification. Analytical thin-layer chromatography (TLC) was achieved on silica gel plates using silica gel 60 GF_254_ (Qingdao Haiyang Chemical Co., Ltd., Qingdao, China). Flash column chromatography purification performed with the silica gel (200–300 mesh, Qingdao Haiyang Chemical Limited Company, Qingdao, China). Melting points were determined by the X-4 microscopic melting point apparatus (Beijing Taike Instruments Co., Ltd., Beijing, China). ^1^H and ^13^C nuclear magnetic resonance (NMR) spectra were recorded on Bruker Avance NEO 400 MHz and 100 or 150 MHz instruments (Bruker BioSpin Corp., Karlsruhe, Germany), respectively, using tetramethylsilane (TMS) as the internal standard. High-resolution mass spectra (HRMS) data were obtained using an Ion trap/time-of-flight (IT/TOF) mass spectrometer coupled with an electrospray ionization (ESI) source (Shimadzu Corp., Kyoto, Japan).

### 3.2. Synthesis

The synthetic route for the compounds B1~C12 were outlined in Figure 3.

The general procedure for the synthesis of compounds B1~7 [27] is presented here.

To a solution of anthranilic acid (A1~7, 5 mmol) in *N*,*N*-dimethylformamide (DMF, 10 mL), anhydrous potassium carbonate (K_2_CO_3_, 0.51 g, 3.7 mmol) was added and heated at 100 °C for 1 h. The solution was cooled to 50 °C and chloroacetone (0.46 g, 5 mmol) was added. The solution was stirred at 50 °C for 1 h, cooled to room temperature, and poured into ice/water (50 mL); then, stirring was continued for 1 h at 0 to 10 °C. Precipitate was observed, then filtered and washed with water (15 mL), and dried under a vacuum to give the products B1~7 as solids in 70~84% yields.

2-oxopropyl 2-amino-5-bromobenzoate (B1): Yellow solid, yield 83.9%, m.p., 85–87 °C. ^1^H NMR (400 MHz, CDCl_3_) *δ* 8.04 (d, *J* = 2.4 Hz, 1H), 7.36 (dd, *J* = 8.8, 2.4 Hz, 1H), 6.58 (d, *J* = 8.8 Hz, 1H), 5.74 (s, 2H), 4.84 (s, 2H), 2.23 (s, 4H).

2-oxopropyl 2-amino-5-iodobenzoate (B2): Brown solid, yield 84.3%, m.p., 81–83 °C. ^1^H NMR (400 MHz, CDCl_3_) *δ* 8.20 (d, *J* = 2.2 Hz, 1H), 7.50 (dd, *J* = 8.7, 2.2 Hz, 1H), 6.47 (d, *J* = 8.7 Hz, 1H), 4.83 (s, 2H), 2.23 (s, 3H).

2-oxopropyl 2-amino-5-methylbenzoate (B3): Black solid, yield 81.7%, m.p., 81–83 °C. ^1^H NMR (400 MHz, CDCl_3_) *δ* 7.74 (dd, *J* = 2.0, 1.0 Hz, 1H), 7.13 (dd, *J* = 8.3, 2.2 Hz, 1H), 6.62 (d, *J* = 8.4 Hz, 1H), 4.82 (s, 2H), 2.24 (s, 3H), 2.23 (s, 3H).

2-oxopropyl 2-amino-4-methoxybenzoate (B4): Yellow solid, yield 80.6%, m.p., 87–89 °C. ^1^H NMR (400 MHz, DMSO-*d*_6_) *δ* 7.69 (d, *J* = 9.0 Hz, 1H), 6.70 (s, 2H), 6.29 (d, *J* = 2.5 Hz, 1H), 6.17 (dd, *J* = 9.0, 2.5 Hz, 1H), 4.88 (s, 2H), 3.74 (s, 3H), 2.13 (s, 3H).

2-oxopropyl 2-amino-4-(trifluoromethyl) benzoate (B5): Green solid, yield 73.6%, m.p., 84–86 °C. ^1^H NMR (400 MHz, CDCl_3_) *δ* 8.07–7.97 (m, 1H), 6.92 (d, *J* = 1.7 Hz, 1H), 6.87 (dd, *J* = 8.5, 1.7 Hz, 1H), 4.87 (s, 2H), 2.24 (s, 3H).

2-oxopropyl 2-amino-4,5-difluorobenzoate (B6): Yellow solid, yield 78.8%, m.p., 88–90 °C. ^1^H NMR (400 MHz, CDCl_3_) *δ* 7.74 (dd, *J* = 11.2, 8.9 Hz, 1H), 6.44 (dd, *J* = 12.0, 6.6 Hz, 1H), 4.83 (s, 2H), 2.23 (s, 3H).

2-oxopropyl 2-amino-4,5-dimethoxybenzoate (B7): Yellow solid, yield 70.6%, m.p., 82–84 °C. ^1^H NMR (400 MHz, CDCl_3_) *δ* 7.36 (s, 1H), 6.15 (s, 1H), 4.81 (s, 2H), 3.88 (s, 3H), 3.84 (s, 3H), 2.23 (s, 2H).

The general procedure for the synthesis of compounds C1~7 [27] is presented here.

The intermediate B1~7 (5 mmol) was heated in reflux *N*-methyl-2-pyrrolidone (NMP, 4 mL) for 8 h and then cooled to 50 °C. Ethyl acetate (10 mL) was added; upon cooling to room temperature, a crude solid precipitate was observed. The precipitate was stirred at 0 to 5 °C for 30 min, then filtered and washed with water (3 mL), cold ethyl acetate (3 mL), and cold dichloromethane (4 mL) to give products C1~7 as solids in 54~71% yields.

6-bromo-3-hydroxy-2-methylquinolin-4(1H)-one (C1): Brown solid, yield 71.7%, m.p., 181–183 °C. ^1^H NMR (400 MHz, DMSO-*d*_6_) *δ* 11.72 (s, 1H), 8.34 (s, 1H), 8.18 (d, *J* = 2.4 Hz, 1H), 7.66 (dd, *J* = 8.9, 2.4 Hz, 1H), 7.48 (d, *J* = 8.9 Hz, 1H), 2.37 (s, 3H). ^13^C NMR (100 MHz, DMSO-*d*_6_) *δ* 167.9, 139.1, 136.4, 133.1, 133.0, 127.0, 124.3, 120.8, 114.5, 14.6. HRMS for C_10_H_9_BrNO_2_^+^ [M + H]^+^ 253.9811, found 253.9815.

3-hydroxy-6-iodo-2-methylquinolin-4(1H)-one (C2): Blank solid, yield 70.4%, m.p., 188–190 °C. ^1^H NMR (400 MHz, DMSO-*d*_6_) *δ* 11.69 (s, 1H), 8.39 (d, *J* = 1.9 Hz, 1H), 7.79 (dd, *J* = 8.7, 2.1 Hz, 1H), 7.34 (d, *J* = 8.7 Hz, 1H), 2.37 (s, 3H). ^13^C NMR (100 MHz, DMSO-*d*_6_) *δ* 167.6, 139.0, 138.3, 136.7, 133.4, 133.1, 124.8, 120.7, 86.2, 14.6. HRMS for C_10_H_9_INO_2_^+^ [M + H]^+^ 301.9672, found 301.9651.

3-hydroxy-2,6-dimethylquinolin-4(1H)-one (C3): Pink solid, yield 68.6%, m.p., 168–170 °C. ^1^H NMR (400 MHz, DMSO-*d*_6_) *δ* 11.47 (s, 1H), 7.94–7.80 (m, 1H), 7.48–7.29 (m, 2H), 2.39 (s, 3H), 2.35 (s, 3H). HRMS for C_11_H_12_NO_2_^+^ [M + H]^+^ 190.0863, found 190.0835.

3-hydroxy-7-methoxy-2-methylquinolin-4(1H)-one (C4): White solid, yield 54.1%, m.p., 206–208 °C. ^1^H NMR (400 MHz, DMSO-*d*_6_) *δ* 11.33 (s, 1H), 7.99 (d, *J* = 8.9 Hz, 1H), 6.93–6.68 (m, 2H), 3.83 (s, 3H), 2.33 (s, 3H). ^13^C NMR (100 MHz, DMSO-*d*_6_) *δ* 169.1, 161.2, 139.5, 138.0, 131.1, 126.8, 117.3, 113.0, 98.3, 55.7, 14.4. HRMS for C_11_H_12_NO_3_^+^ [M + H]^+^ 206.0812, found 206.0789.

3-hydroxy-2-methyl-7-(trifluoromethyl)quinolin-4(1H)-one (C5): White solid, yield 57.9%, m.p., 187–189 °C. ^1^H NMR (400 MHz, DMSO-*d*_6_) *δ* 11.89 (s, 1H), 8.31 (d, *J* = 8.5 Hz, 1H), 7.87 (s, 1H), 7.57–7.28 (m, 1H), 2.41 (d, *J* = 1.6 Hz, 3H). ^13^C NMR (100 MHz, DMSO-*d*_6_) *δ* 168.5, 139.9, 136.8, 133.73, 130.2 (d, *J* = 31.6 Hz), 127.1, 124.8, 124.5 (q, *J* = 270 Hz), 117.37 (q, *J* = 3.2 Hz), 115.83 (q, *J* = 4.6 Hz) 14.74. HRMS for C_11_H_9_F_3_NO_2_^+^ [M + H]^+^ 244.0580, found 244.0558.

6,7-difluoro-3-hydroxy-2-methylquinolin-4(1H)-one (C6): White solid, yield 58.7%, m.p., 178–180 °C.^1^H NMR (400 MHz, DMSO-*d*_6_) *δ* 11.75 (s, 1H), 7.93 (dd, *J* = 11.2, 8.8 Hz, 1H), 7.44 (dd, *J* = 11.4, 6.8 Hz, 1H), 2.36 (s, 3H). ^13^C NMR (100 MHz, DMSO-*d*_6_) *δ* 168.1 (d, *J* = 2.8 Hz), 151.7 (dd, *J* = 249.3, 16.0 Hz), 146.45 (dd, *J* = 243.5, 14.7 Hz), 138.5, 134.4 (d, *J* = 10.7 Hz), 133.2, 119.7 (d, *J* = 5.0 Hz), 111.66 (dd, *J* = 16.3, 2.0 Hz), 105.8 (d, *J* = 20.6 Hz), 14.6. HRMS for C_10_H_8_F_2_NO_2_^+^ [M + H]^+^ 212.0518, found 212.0489.

3-hydroxy-6,7-dimethoxy-2-methylquinolin-4(1H)-one (C7): White solid, yield 67.2%, m.p., 191–193 °C. ^1^H NMR (400 MHz, DMSO-*d*_6_) *δ* 11.32 (s, 1H), 7.39 (s, 1H), 6.89 (s, 1H), 3.84 (d, *J* = 5.6 Hz, 6H), 2.32 (s, 3H). ^13^C NMR (100 MHz, DMSO-*d*_6_) *δ* 168.0, 152.5, 146.3, 137.7, 133.6, 130.1, 116.7, 103.8, 98.8, 56.0, 55.9, 14.4. HRMS for C_12_H_14_NO_4_^+^ [M + H]^+^ 236.0917, found 236.0893.

The general procedure for the synthesis of compounds D1~7 [27] is presented here.

To a solution of compounds B1~7 (2 mmol) in DMF (6 mL), anhydrous K_2_CO_3_ (0.83 g, 6 mmol) and dibromomethane (CH_2_Br_2_, 0.3 mL, 4 mol) were added and the mixture was heated at 120 °C under the nitrogen atmosphere for 2–4 h. When the reaction was complete, as determined by TLC analysis, the solution was cooled to room temperature and then partitioned between ethyl acetate (EA, 30 mL) and water (15 mL). The organic layer was washed with water (10 mL × 3); after drying by anhydrous sodium sulfate (Na_2_SO_4_), the mixture was concentrated to obtain the crude product and then this was purified by flash column chromatography on silica gel to obtain products D1~7 as solids in 83~91% yields.

8-bromo-4-methyl-[1,3]dioxolo[4,5-c]quinoline (D1): Pink solid, yield 89.7%, m.p., 97–99 °C. ^1^H NMR (400 MHz, CDCl_3_) *δ* 7.91 (d, *J* = 2.2 Hz, 1H), 7.80 (d, *J* = 9.1 Hz, 1H), 7.58 (dd, *J* = 9.1, 2.3 Hz, 1H), 6.25 (s, 2H), 2.62 (s, 3H). ^13^C NMR (100 MHz, CDCl_3_) *δ* 147.4, 144.1, 143.8, 140.4, 131.3, 130.5, 122.2, 119.5, 116.5, 103.0, 19.3. HRMS for C_11_H_9_BrNO_2_^+^ [M + H]^+^ 265.9811, found 265.9818.

8-iodo-4-methyl-[1,3]dioxolo[4,5-c]quinoline(D2): White solid, yield 90.1%, m.p., 103–105 °C. ^1^H NMR (400 MHz, CDCl_3_) *δ* 8.16 (d, *J* = 1.9 Hz, 1H), 7.76 (dd, *J* = 9.0, 2.0 Hz, 1H), 7.68 (d, *J* = 9.1 Hz, 1H), 6.25 (s, 2H), 2.63 (s, 3H). ^13^C NMR (100 MHz, CDCl_3_) *δ* 147.2, 144.3, 144.0, 140.3, 136.6, 130.4, 128.9, 117.2, 103.1, 90.9, 19.3. HRMS for C_11_H_9_INO_2_^+^ [M + H]^+^ 313.9672, found 313.9651.

4,8-dimethyl-[1,3]dioxolo[4,5-c]quinoline (D3): Yellow solid, yield 93.7%, m.p., 93–95 °C. ^1^H NMR (400 MHz, CDCl_3_) *δ* 7.84 (d, *J* = 8.7 Hz, 1H), 7.51 (dt, *J* = 2.0, 1.0 Hz, 1H), 7.36 (dd, *J* = 8.8, 2.0 Hz, 1H), 6.20 (s, 2H), 2.62 (s, 3H), 2.48 (s, 3H). ^13^C NMR (100 MHz, CDCl_3_) *δ* 147.8, 144.0, 142.5, 139.7, 135.6, 130.3, 128.4, 118.5, 115.4, 102.6, 21.6, 19.2. HRMS for C_12_H_12_NO_2_^+^ [M + H]^+^ 202.0863, found 202.0848.

7-methoxy-4-methyl-[1,3]dioxolo[4,5-c]quinoline (D4): Yellow solid, yield 91.6%, m.p., 117–119 °C. ^1^H NMR (400 MHz, CDCl_3_) *δ* 7.66 (d, *J* = 9.1 Hz, 1H), 7.31 (d, *J* = 2.4 Hz, 1H), 7.10 (dd, *J* = 9.1, 2.4 Hz, 1H), 6.20 (s, 2H), 3.91 (s, 3H), 2.62 (s, 3H). ^13^C NMR (100 MHz, CDCl_3_) *δ* 159.7, 148.9, 147.0, 143.1, 138.7, 121.0, 119.3, 110.15, 106.6, 102.5, 55.4, 19.2. HRMS for C_12_H_12_NO_3_^+^ [M + H]^+^ 218.0812, found 218.0811.

4-methyl-7-(trifluoromethyl)-[1,3]dioxolo[4,5-c]quinoline (D5): White solid, yield 84.1%, m.p., 107–109 °C. ^1^H NMR (400 MHz, CDCl_3_) *δ* 8.29 (s, 1H), 7.89 (d, *J* = 8.7 Hz, 1H), 7.60 (dd, *J* = 8.7, 1.7 Hz, 1H), 6.31 (s, 2H), 2.68 (s, 3H). ^13^C NMR (100 MHz, CDCl_3_) *δ* 148.3, 145.4, 144.0, 141.2, 129.7 (q, *J* = 32.7 Hz), 126.7 (q, *J* = 4.5 Hz), 124.08 (d, *J* = 272.3 Hz), 121.56–120.54 (m, 2C) 116.9, 103.3, 19.4. HRMS for C_12_H_9_F_3_NO_2_^+^ [M + H]^+^ 256.0580, found 256.0570.

7,8-difluoro-4-methyl-[1,3]dioxolo[4,5-c]quinoline (D6): Pink solid, yield 83.2%, m.p., 104–106 °C. ^1^H NMR (400 MHz, CDCl_3_) *δ* 7.71 (dd, *J* = 11.7, 7.6 Hz, 1H), 7.46 (dd, *J* = 10.1, 8.4 Hz, 1H), 6.25 (s, 2H), 2.62 (s, 3H). ^13^C NMR (100 MHz, CDCl_3_) *δ* 151.4 (dd, *J* = 252.2, 16.3 Hz), 149.8 (dd, *J* = 252.0, 16.4 Hz),148.3 (dd, *J* = 4.0, 2.6 Hz), 144.0 (d, *J* = 2.6 Hz), 142.3 (d, *J* = 11.4 Hz), 140.1 (d, *J* = 2.6 Hz), 115.2 (dd, *J* = 16.8, 1.5 Hz), 111.9 (d, *J* = 8.4 Hz), 105.5 (dd, *J* = 19.6, 1.5 Hz), 103.0, 19.2. HRMS for C_11_H_8_F_2_NO_2_^+^ [M + H]^+^ 224.0518, found 224.0485.

7,8-dimethoxy-4-methyl-[1,3]dioxolo[4,5-c]quinoline (D7): Yellow solid, yield 89.6%, m.p., 110–112 °C. ^1^H NMR (400 MHz, CDCl_3_) *δ* 7.30 (d, *J* = 1.7 Hz, 1H), 6.94 (s, 1H), 6.18 (s, 2H), 3.98 (s, 6H), 2.59 (s, 3H). ^13^C NMR (100 MHz, CDCl_3_) *δ* 146.6, 144.7, 143.0, 137.6, 135.5, 134.2, 105.2, 102.8, 97.6, 92.6, 51.3, 51.2, 14.1. HRMS for C_13_H_14_NO_4_^+^ [M + H]^+^ 248.0917, found 248.0881.

The general procedure for the synthesis of compounds D8~12 [29] is presented here.

A mixture of Tetrakis(triphenylphosphine) palladium(Pd(PPh_3_)_4_, 0.05 mmol), anhydrous K_2_CO_3_ (0.69 g, 5 mmol), compounds D1 (0.26 g, 1 mmol), and aryl boronic acid (1 mmol) in 1,4-Dioxane (10 mL) was degassed by flushing with nitrogen. After being stirred at 100 °C for 10 h, the mixture was then partitioned between EA (30 mL) and water (10 mL). The organic layer was separated, washed with water, dried, and concentrated. The residue was then purified by column chromatography to give the coupled products D8~12 as solids in 50~66% yields.

4-methyl-8-phenyl-[1,3]dioxolo[4,5-c]quinoline (D8): White solid, yield 90.8%, m.p., 114–116 °C. ^1^H NMR (400 MHz, CDCl_3_) *δ* 8.02 (dd, *J* = 9.0, 0.7 Hz, 1H), 7.96 (dd, *J* = 2.1, 0.7 Hz, 1H), 7.81 (dd, *J* = 8.9, 2.1 Hz, 1H), 7.74–7.67 (m, 2H), 7.51–7.43 (m, 2H), 7.41–7.34 (m, 1H), 6.25 (d, *J* = 0.8 Hz, 2H), 2.66 (s, 3H). ^13^C NMR (100 MHz, CDCl_3_) *δ* 148.5, 144.8, 143.5, 140.3, 140.1, 138.3, 129.2, 128.9, 127.7, 127.6, 127.3, 117.5, 115.7, 102.8, 19.4. HRMS for C_17_H_14_NO_2_^+^ [M + H]^+^ 264.1019, found 264.1023.

4-methyl-8-(*p*-tolyl)-[1,3]dioxolo[4,5-c]quinoline (D9): White solid, yield 92.8%, m.p., 148–150 °C. ^1^H NMR (400 MHz, CDCl_3_) *δ* 8.00 (d, *J* = 9.0 Hz, 1H), 7.93 (d, *J* = 2.0 Hz, 1H), 7.80 (dd, *J* = 8.9, 2.1 Hz, 1H), 7.63–7.56 (m, 2H), 7.31–7.26 (m, 2H), 6.24 (s, 2H), 2.65 (s, 3H), 2.41 (s, 3H). ^13^C NMR (100 MHz, CDCl_3_) *δ* 148.4, 144.7, 143.3, 140.0, 138.2, 137.6, 137.4, 129.7, 129.1, 127.6, 127.1, 117.0, 115.7, 102.8, 21.2, 19.3. HRMS for C_18_H_16_NO_2_^+^ [M + H]^+^ 278.1176, found 278.1183.

8-(4-chlorophenyl)-4-methyl-[1,3]dioxolo[4,5-c]quinoline (D10): Yellow solid, yield 95.3%, m.p., 170–172 °C. ^1^H NMR (400 MHz, CDCl_3_) *δ* 8.01 (d, *J* = 8.9 Hz, 1H), 7.90 (d, *J* = 2.1 Hz, 1H), 7.75 (dd, *J* = 8.9, 2.2 Hz, 1H), 7.61 (d, *J* = 8.6 Hz, 2H), 7.43 (d, *J* = 8.5 Hz, 2H), 6.26 (s, 2H), 2.66 (s, 3H). ^13^C NMR (100 MHz, CDCl_3_) *δ* 148.5, 144.7, 143.8, 140.2, 138.7, 137.0, 133.9, 129.4, 129.1, 128.5, 127.2, 117.5, 115.6, 102.9, 19.3. HRMS for: C_17_H_13_ClNO_2_^+^ [M + H]^+^ 298.0629, found 298.0631.

8-(3,4-dimethoxyphenyl)-4-methyl-[1,3]dioxolo[4,5-c]quinoline (D11): White solid, yield 89.7%, m.p., 120–122 °C. ^1^H NMR (400 MHz, CDCl_3_) *δ* 8.03–7.97 (m, 1H), 7.92–7.88 (m, 1H), 7.79 (dd, *J* = 8.9, 2.1 Hz, 1H), 7.30–7.24 (m, 1H), 7.21 (d, *J* = 2.2 Hz, 1H), 6.98 (d, *J* = 8.3 Hz, 1H), 6.26 (s, 2H), 3.96 (d, *J* = 16.7 Hz, 6H), 2.66 (s, 3H). ^13^C NMR (100 MHz, CDCl_3_) *δ* 149.3, 149.0, 148.3, 144.6, 143.3, 140.1, 138.1, 133.2, 129.1, 127.5, 119.7, 116.7, 115.7, 111.6, 110.5, 102.8, 56.0, 56.0, 19.3. HRMS for C_19_H_18_NO_4_^+^ [M + H]^+^ 324.1230, found 324.1245.

4-methyl-8-(thiophen-2-yl)-[1,3]dioxolo[4,5-c]quinoline (D12): White solid, yield 87.2%, m.p., 130–132 °C. ^1^H NMR (400 MHz, Chloroform-*d*) *δ* 7.98–7.91 (m, 2H), 7.81 (dd, *J* = 9.1, 2.0 Hz, 1H), 7.43 (dd, *J* = 3.6, 1.1 Hz, 1H), 7.33 (dd, *J* = 5.1, 1.1 Hz, 1H), 7.11 (dd, *J* = 5.1, 3.6 Hz, 1H), 6.25 (s, 2H), 2.64 (s, 3H). ^13^C NMR (100 MHz, CDCl_3_) *δ* 148.4, 144.7, 143.6, 143.4, 140.3, 131.6, 129.3, 128.3, 126.5, 125.6, 124.0, 115.8, 115.7, 102.9, 19.3. HRMS for C_15_H_12_NO_2_S^+^ [M + H]^+^ 270.0583, found 270.0583.

The general procedure for the synthesis of compounds C8~12 [30] is presented here.

A suspension of compounds D8~12 (1 mmol) in 5 mL of diluted hydrochloric acid (HCl/H_2_O, 3 mol/L) was heated to 75 °C for 0.5 h. The suspension turned into a clear solution. The solution continued heating and refluxed for 6 h. A crude solid precipitate was observed. Then, the mixture was adjusted to pH 7 with saturated sodium carbonate solution. The precipitate was filtered, washed with water (3 mL), ethanol (3 mL), and dichloromethane (4 mL), and dried under vacuum to provide products C8~12 as solids in 70~84% yields.

3-hydroxy-2-methyl-6-phenylquinolin-4(1H)-one (C8): White solid, yield 51.4%, m.p., > 300 °C ^1^H NMR (400 MHz, DMSO-*d*_6_) *δ* 8.53 (s, 1H), 8.11 (q, *J* = 8.5 Hz, 2H), 7.83 (d, *J* = 7.6 Hz, 2H), 7.54 (t, *J* = 7.5 Hz, 2H), 7.44 (t, *J* = 7.2 Hz, 1H), 2.70 (s, 2H). ^13^C NMR (150 MHz, DMSO-*d*_6_) *δ* 157.6, 147.0, 139.0, 138.6, 136.9, 134.9, 131.0, 129.7, 128.8, 127.6, 120.4, 120.3, 120.2, 16.3. HRMS for C_16_H_14_NO_2_^+^ [M + H]^+^ 252.1019, found 252.1022.

3-hydroxy-2-methyl-6-(*p*-tolyl)quinolin-4(1H)-one (C9): White solid, yield 59.9%, m.p., > 300 °C. ^1^H NMR (400 MHz, DMSO-*d*_6_) *δ* 14.39 (s, 1H), 8.49 (d, *J* = 1.9 Hz, 1H), 8.10 (dd, *J* = 8.9, 2.0 Hz, 1H), 8.02 (d, *J* = 8.9 Hz, 1H), 7.82–7.61 (m, 2H), 7.34 (d, *J* = 8.0 Hz, 2H), 2.68 (s, 3H), 2.38 (s, 3H). ^13^C NMR (150 MHz, DMSO-*d*_6_) *δ* 144.0, 138.2, 137.7, 137.3, 136.3, 135.2, 130.6, 130.3, 127.3, 120.9, 120.1, 120.0, 21.2, 16.0. HRMS for C_17_H_16_NO_2_^+^ [M + H]^+^ 266.1176, found 266.1153.

6-(4-chlorophenyl)-3-hydroxy-2-methylquinolin-4(1H)-one (C10): White solid, yield 50.7%, m.p., > 300 °C. ^1^H NMR (400 MHz, DMSO-*d*_6_) *δ* 11.66 (s, 1H), 8.33 (s, 1H), 7.88 (d, *J* = 8.6 Hz, 1H), 7.77 (d, *J* = 8.1 Hz, 2H), 7.61 (d, *J* = 8.7 Hz, 1H), 7.54 (d, *J* = 8.2 Hz, 2H), 2.39 (s, 3H). ^13^C NMR (150 MHz, DMSO-*d*_6_) *δ* 144.9, 138.0, 137.3, 136.4, 135.3, 133.6, 130.5, 129.6, 129.2, 120.8, 120.7, 120.3, 16.1. HRMS for C_16_H_13_ClNO_2_^+^ [M + H]^+^ 286.0629, found 286.0633.

6-(3,4-dimethoxyphenyl)-3-hydroxy-2-methylquinolin-4 (1H)-one (C11): White solid, yield 61.3%, m.p., > 300 °C. ^1^H NMR (400 MHz, DMSO-*d*_6_) *δ* 14.71 (s, 1H), 8.55 (d, *J* = 2.0 Hz, 1H), 8.15 (dd, *J* = 8.9, 2.0 Hz, 1H), 8.05 (d, *J* = 8.8 Hz, 1H), 7.40 (d, *J* = 2.2 Hz, 1H), 7.36 (dd, *J* = 8.3, 2.2 Hz, 1H), 7.10 (d, *J* = 8.4 Hz, 1H), 3.90 (s, 3H), 3.83 (s, 3H), 2.71 (s, 3H). ^13^C NMR (150 MHz, DMSO-*d*_6_) *δ* 157.7, 149.8, 149.7, 146.2, 138.3, 137.0, 134.7, 131.6, 130.8, 120.5, 120.2, 120.0, 119.4, 112.7, 111.0, 56.5, 56.1, 16.2. HRMS for C_18_H_19_NO_4_^+^ [M + H]^+^ 312.1230, found 312.1231.

3-hydroxy-2-methyl-6-(thiophen-2-yl)quinolin-4(1H)-one (C12): White solid, yield 66.6%, m.p., > 300 °C. ^1^H NMR (400 MHz, DMSO-*d*_6_) *δ* 14.40 (s, 1H), 8.45–8.40 (m, 1H), 8.16–8.07 (m, 1H), 8.01–7.96 (m, 1H), 7.70 (d, *J* = 3.7 Hz, 1H), 7.66 (d, *J* = 4.9 Hz, 1H), 7.21 (dd, *J* = 5.1, 3.6 Hz, 1H), 2.66 (s, 3H). ^13^C NMR (150 MHz, DMSO-*d*_6_) *δ* 158.8, 144.3, 142.6, 137.5, 135.2, 131.4, 129.4, 129.3, 127.4, 125.6, 121.1, 120.4, 118.7, 15.9. HRMS for C_14_H_12_NO_2_S^+^ [M + H]^+^ 258.0583, found 258.0553.

### 3.3. Nematocidal Assays [31]

#### 3.3.1. Cultivation of *M. incognita*

The eggs of *M. incognita* were collected from the roots of nematode-infected cucumber plants, disinfected with a 0.5% sodium hypochlorite solution for 3 min, rinsed with sterile water 5 to 7 times, and then incubated at 25 °C in the dark. All second-stage juveniles (J2s) of *M. incognita* were collected for the experiments within 7 days.

#### 3.3.2. In Vitro Nematocidal Assays

First, analogues were dissolved in DMF to prepare a stock solution, which was then diluted to different concentrations, ensuring that the final concentration of DMF did not exceed 0.5% by volume. *M. incognita* (J2) were suspended in sterile water to a concentration of 100–120 nematodes/mL. Next, 1 mL of nematode solution and 1 mL of test compound solution was added into a well of a 12-well plate. The solution was then oscillated to mix nematodes and compound evenly and cultured at 25 °C in the dark. Sterile water with an equal amount of DMF served as the blank control, and avermectin as a positive control. The mortality of the nematodes was determined through counts after 24, 48, and 72 h using a stereomicroscope. Each concentration of the test compound had three replicates. The correct mortality rate (MR) of *M. incognita* was calculated according to the formula.

$$\mathrm{Correct}\;\mathrm{MR}=\frac{\;\mathrm{MRtreated}\;-\;\mathrm{MRcontrol}}{1-\mathrm{MRcontrol}}\times 100$$

where MR_treated_ and MR_control_ are the mortality rates for the treated and control groups, respectively.

#### 3.3.3. In Vivo Nematocidal Assays

A total of 1.5 kg of air-dried and steam-sterilized soil (sand/nursery soil, 1:1, *v*/*v*) was added to 17 cm plastic pots. Cucumber seedlings at the two-leaf stage with the same growth were selected, and 100 mL of test compounds solution at 100 and 200 μg/mL was injected into the soil around the roots of cucumber plants. The following day, three thousand newly hatched J2 *M. incognita* were inoculated through three 10 cm holes drilled around the roots of the cucumber plants. Avermectin (10 μg/mL) was used as a positive control and 0.5% Tween-80 aqueous solution was used as a blank control. Each concentration was repeated three times, with each replicate consisting of five plants. Cucumber seedlings were grown under normal water and fertilizer management in the greenhouse at 25 °C. The number of root knots produced on the roots of cucumber plants was counted, and the control effect (CE) was calculated after 60 days by the following formula:

$$\mathrm{CE}=1-\frac{\mathrm{Total}\;\mathrm{number}\;\mathrm{of}\;\mathrm{root}\;\mathrm{knots}\;\left(\mathrm{Treated}\;\mathrm{group}\right)}{\mathrm{Total}\;\mathrm{number}\;\mathrm{of}\;\mathrm{root}\;\mathrm{knots}\;\left(\mathrm{Control}\;\mathrm{group}\right)}\times 100$$

### 3.4. Antifungal Activity Assay

#### 3.4.1. In Vitro Antifungal Assay

The mycelial growth rate method [32] was employed to screen in vitro antifungal activity of the title compounds against eight phytopathogenic fungi. The title compounds were dissolved in DMF before mixing with potato dextrose agar (PDA) medium, and the concentration of test compounds in the medium was fixed at 50 μg/mL. The mycelia disks (5 mm) were inoculated in the center of PDA medium (three replicates for each treatment) and incubated at 27 ± 1 °C for 4 to 7 days. DMF without compounds served as the negative control and Azoxystrobin as the positive control. The inhibition of the title compound against these fungi was calculated by the following formula:

$$I=\frac{C\;-\;T}{C\;-\;0.5}\;\times 100$$

I represents the inhibition rate, C represents the diameter of fungal growth on untreated PDA, and T represents the diameter of fungi on treated PDA.

#### 3.4.2. In Vivo Antifungal Assay

According to the reported procedures [33], healthy tomatoes of even sizes were washed with clean water, followed by a wipe with 75% ethanol and three rinses with sterile water; they were then air-dried in a clean bench. The tomatoes were evenly sprayed with compounds at concentrations of 100 and 200 μg/mL and subsequently cultivated at 22 °C for 24 h before inoculation with strain *Botrytis cinerea*. DMF 1% in 10 mL water was used as the blank control and Pyrimethanil was used as the positive control; each concentration was triplicated, with each replicate comprising five tomatoes. The results were observed as diameters of symptoms after 4-day cultivation. The efficacy of disease control was calculated by the following formula:

$$I=\frac{Ø-\;T}{Ø\;-\;0.5}\;\times 100$$

I represents the inhibition rate; Ø is the diameter of the negative control; T is the diameter of the treatment.

### 3.5. Statistical Analysis

SPSS (Statistical Package, Version 20.0, Armonk, NY, USA) was adopted to conduct statistical analyses. All obtained results were calculated as the mean value and SD. The acquired data were processed using one-way analysis of variation (ANOVA), and the statistical significance was determined at *p* < 0.05.

## 4. Conclusions

In summary, by leveraging a strategy of structural simplification and integrating our prior research findings, the current study successfully highlighted quinoline derivatives with a simplified version of the seven-membered oxygen-bridged ring structure of Waltherione A, with the aim of developing novel nematicides. This study demonstrates that quinoline derivatives exhibit superior nematocidal activity compared to quinolone derivatives, with compound D1 exhibiting the highest potency (LC_50_ 23.06 μg/mL). At a concentration of 200 μg/mL, compound D1 achieves a 61.57% inhibition rate against nematodes in vivo, demonstrating its efficacy in controlling nematode infection. Although the nematocidal activity of the compound designed in this study is inferior to that of the lead compound, Waltherione A, it exhibits significantly enhanced activity compared to the derivative retaining the seven-membered oxygen-bridged ring in our previous work, thereby simplifying the quinolone structure. This suggests that the quinolone moiety is critical for the nematocidal efficacy of Waltherione A. Additionally, the target compounds demonstrated inhibitory activity against eight prevalent plant pathogens, with quinoline-derived compounds exhibiting superior fungicidal activity compared to the quinolone structure. Notably, compound D1 showed enhanced efficacy against *B. cinerea*. In vivo studies revealed that the inhibitory effects of higher concentrations (200 μg/mL, 96.65%) were slightly superior to those of the positive control Pyrimethanil (200 μg/mL, 90.30%). These findings suggest that quinoline structures warrant further study for the development of nematicides, addressing the critical need for effective treatments against plant parasitic nematode diseases in the face of a limited arsenal of nematicides.

## Figures and Tables

**Table 1:** Nematicidal activities of target compounds against *M. incognita* in vitro at 200 μg/mL for 72 h.

Compound	Mortality (%)	Compound	Mortality (%)
C1	27.5 ± 4.7 ^e^	D1	100 ± 0.0 ^a^
C2	22.1 ± 0.4 ^efg^	D2	100 ± 0.0 ^a^
C3	24.7 ± 0.6 ^efg^	D3	100 ± 0.0 ^a^
C4	17.9 ± 0.7 ^g^	D4	46.3 ± 0.8 ^d^
C5	20.0 ± 1.5 ^fg^	D5	54.6 ± 10.8 ^c^
C6	22.1 ± 1.4 ^efg^	D6	45.4 ± 15.6 ^d^
C7	26.1 ± 1.2 ^ef^	D7	83.7 ± 1.3 ^b^
C8	5.8 ± 1.1 ^hi^	D8	11.3 ± 1.7 ^h^
C9	9.2 ± 1.1 ^hi^	D9	8.9 ± 0.1 ^hi^
C10	7.8 ± 1.7 ^hi^	D10	3.2 ± 1.2 ^i^
C11	3.6 ± 2.3 ^i^	D11	1.9 ± 0.2 ^i^
C12	3.7 ± 0.2 ^i^	D12	100 ± 0.0 ^a^
avermectin	100 ± 0.00 ^a^	Tioxazafen	100 ± 0.0 ^a^

Note: Lowercase letters (^a^, ^b^, ^c^, ^d^, ^e^, ^f^, ^g^, ^h^, and ^i^) represent the results of one-way ANOVA among different treatments at the same concentration, with significant differences indicated by Duncan’s analysis at *p* ≤ 0.05. These letters are marked in descending order of inhibition rate, with ‘^a^’ indicating the highest and ‘^i^’ the lowest rate of inhibition among the groups.

**Table 2:** LC_50_ of compounds D1~3, D12, tioxazafen, and avermectin against *M. incognita* for 72 h.

Compound	LC_50_ (μg/mL)	Toxic Regression Equations	R^2^
D1	23.06	y = −3.23 + 2.38x	0.978
D2	38.58	y = −3.45 + 2.17x	0.969
D3	30.63	y = −4.22 + 2.58x	0.981
D12	68.15	y = −8.52 + 4.65x	0.992
tioxazafen	17.44	y = −4.08 + 3.29x	0.996
avermectin	0.21	y = 1.26 + 1.83x	0.960

**Table 3:** Effect of compound D1 and D3 on control of *M. incognita* in cucumber pot experiment.

Treatment		Total Number of Root Knots	Inhibition Rate (%)
D1	100 μg/mL	59 ± 3.55 ^c^	32.18 ± 0.68 ^d^
	200 μg/mL	34 ± 1.31 ^e^	61.57 ± 1.28 ^b^
D3	100 μg/mL	68 ± 5.14 ^b^	21.83 ± 0.73 ^e^
	200 μg/mL	48 ± 3.15 ^d^	44.82 ± 1.05 ^c^
avermectin	10 μg/mL	31 ± 1.01 ^e^	64.36 ± 1.11 ^a^
blank control		87 ± 6.53 ^a^	-

Note: Lowercase letters represent one-way ANOVA results between different groups at the same time point (Duncan analysis *p* ≤ 0.05). ^a^, ^b^, ^c^, ^d^, and ^e^ indicate significant differences between different groups, and ^a^, ^b^, ^c^, ^d^, and ^e^ are labeled in descending order of inhibition rate.

**Table 4:** Inhibitory rate of target compounds at 50 μg/mL on plant pathogen mycelium growth (%).

Compound	Inhibitory Rate (%)							
	*F.g*	*C.m*	*P.c*	*F.o*	*C.s*	*P.o*	*R.s*	*B.c*
C1	29.4 ± 1.2 ^ij^	76.5 ± 2.2 ^c^	31.9 ± 1.0 ^e^	61.2 ± 0.2 ^b^	47.1 ± 0.5 ^c^	41.2 ± 2.1 ^de^	8.2 ± 1.8 ^hi^	29.4 ± 1.2 ^g^
C2	58.0 ± 4.3 ^de^	37.2 ± 1.6 ^i^	57.1 ± 2.4 ^c^	62.0 ± 4.0 ^b^	38.8 ± 0.7 ^d^	53.3 ± 6.7 ^c^	43.8 ± 2.2 ^e^	73.1 ± 5.3 *^c^*
C3	18.8 ± 2.7 ^kl^	69.4 ± 0.0 ^e^	67.5 ± 1.1 ^b^	30.6 ± 6.5 ^f^	0.0 ± 0.0 ^l^	0.0 ± 0.0 ^k^	78.8 ± 1.0 ^a^	66.7 ± 0.2 ^d^
C4	7.9 ± 2.1 ^mn^	11.4 ± 1.0 ^l^	26.6 ± 0.9 ^fgh^	12.4 ± 1.5 ^h^	15.6 ± 1.5 ^hi^	0.0 ± 0.0 ^k^	0.0 ± 0.0 ^k^	0.0 ± 0.0 ^j^
C5	8.4 ± 1.0 ^mn^	37.0 ± 0.5 ^i^	26.9 ± 1.3 ^fgh^	20.6 ± 1.9 ^g^	13.3 ± 1.4 ^i^	33.40 ± 2.1 ^gh^	0.0 ± 0.0 ^k^	0.0 ± 0.0 ^j^
C6	0.0 ± 0.0 ^o^	7.6 ± 1.4 ^m^	10.2 ± 1.0 ^j^	38.3 ± 1.7 ^e^	39.1 ± 0.3 ^d^	28.9 ± 0.7 ^h^	18.9 ± 0.4 ^g^	28.4 ± 0.6 g
C7	0.0 ± 0.0 ^o^	0.0 ± 0.0 ^n^	1.4 ± 1.0 ^m^	13.9 ± 1.7 ^gh^	21.5 ± 0.9 ^g^	37.1 ± 0.1 ^efg^	9.2 ± 2.2 ^h^	9.1 ± 1.1 ^f^
C8	6.2 ± 0.6 ^mn^	1.7 ± 0.8 ^n^	0.0 ± 0.0 ^m^	7.9 ± 0.4 ^hi^	0.0 ± 0.0 ^l^	8.4 ± 0.4 ^j^	4.1 ± 0.6 ^j^	3.0 ± 0.7 ^ij^
C9	11.3 ± 0.3 ^m^	2.1 ± 0.2 ^n^	3.2 ± 0.2 ^lm^	5.0 ± 0.3 ^ij^	0.0 ± 0.0 ^l^	7.7 ± 0.8 ^j^	6.9 ± 0.4 ^hi^	4.1 ± 0.2 ^i^
C10	4.0 ± 0.4 ^o^	8.0 ± 0.4 ^m^	6.1 ± 0.3 ^kl^	6.2 ± 0.3 ^i^	3.5 ± 0.5 ^k^	4.6 ± 0.2 ^jk^	3.7 ± 0.7 ^jk^	5.2 ± 0.3 ^i^
C11	8.7 ± 1.7 ^mn^	5.9 ± 0.1 ^m^	7.0 ± 0.7 ^k^	8.5 ± 0.4 ^hi^	8.5 ± 0.6 ^j^	1.7 ± 0.7 ^k^	6.3 ± 0.4 ^hij^	5.0 ± 0.8 ^i^
C12	9.1 ± 1.7 ^mn^	10.9 ± 0.7 ^l^	23.3 ± 0.1 ^h^	9.1 ± 0.2 ^hi^	7.7 ± 0.9 ^j^	9.3 ± 0.4 ^j^	4.7 ± 0.8 ^ij^	6.5 ± 0.5 ^fi^
D1	84.7 ± 0.2 ^a^	86.3 ± 1.4 ^a^	76.5 ± 1.7 ^a^	55.3 ± 1.5 ^c^	57.7 ± 1.9 ^a^	77.7 ± 1.4 ^a^	78.8 ± 0.8 ^a^	90.6 ± 0.9 ^a^
D2	43.0 ± 3.0 ^fg^	63.4 ± 1.6 ^f^	34.0 ± 0.9 ^e^	32.0 ± 5.9 ^f^	35.2 ± 1.7 ^e^	72.7 ± 4.5 ^ab^	63.0 ± 1.1 ^c^	94.0 ± 1.5 ^a^
D3	57.6 ± 1.4 ^de^	43.5 ± 1.9 ^h^	28.2 ± 1.1 ^f^	35.3 ± 2.0 ^ef^	15.3 ± 1.8 ^hi^	74.6 ± 0.8 ^ab^	43.3 ± 0.4 ^e^	82.8 ± 0.7 ^b^
D4	34.9 ± 1.1 ^hi^	37.8 ± 1.5 ^i^	23.7 ± 1.0 ^gh^	38.5 ± 1.4 ^e^	8.8 ± 1.1 ^j^	41.5 ± 0.6 ^de^	78.1 ± 0.5 ^a^	10.0 ± 0.7 ^f^
D5	17.6 ± 1.5 ^l^	0.0 ± 0.0 ^n^	18.5 ± 1.9 ^i^	23.4 ± 1.1 ^g^	6.2 ± 0.7 ^jk^	17.8 ± 1.2 ^i^	0.0 ± 0.0 ^k^	43.4 ± 0.6 ^f^
D6	24.1 ± 1.6 ^jk^	60.5 ± 1.1 ^fg^	24.1 ± 0.3 ^gh^	19.3 ± 2.1	17.8 ± 0.9 ^h^	39.8 ± 1.4 ^ef^	57.6 ± 0.6 ^d^	68.0 ± 1.6 ^d^
D7	30.2 ± 2.0 ^i^	21.2 ± 1.5 ^k^	25.7 ± 1.0 ^fgh^	0.0 ± 0.0 ^j^	0.0 ± 0.0 ^l^	28.9 ± 1.6 ^h^	57.1 ± 1.1 ^d^	0.0 ± 0.0 ^j^
D8	37.6 ± 0.9 ^gh^	35.3 ± 1.6 ^i^	17.7 ± 1.6 ^i^	35.3 ± 0.1 ^ef^	29.4 ± 2.2 ^f^	57.7 ±1.5 ^c^	29.4 ± 1.9 ^f^	41.2 ± 0.7 ^f^
D9	44.7 ± 2.2 ^f^	29.4 ± 1.8 ^j^	27.1 ± 1.9 ^fg^	47.1 ± 0.5 ^d^	5.8 ± 0.6 ^jk^	35.3 ± 2.6 ^fg^	0.0 ± 0.0 ^k^	61.3 ± 0.2 ^e^
D10	67.3 ± 7.7 ^bc^	81.4 ± 1.0 ^b^	27.1 ± 1.9 ^fg^	47.1 ± 0.5 ^d^	5.8 ± 0.6 ^jk^	73.6 ± 0.9 ^ab^	59.4 ± 4.7 ^cd^	72.6 ± 0.7 ^cd^
D11	52.7 ± 0.9 ^e^	59.0 ± 0.6 ^g^	40.4 ± 3.1 ^d^	57.3 ± 0.9 ^bc^	46.6 ± 3.0 ^c^	70.3 ± 1.7 ^b^	62.6 ± 0.7 ^c^	70.2 ± 3.2 ^cd^
D12	62.4 ± 1.3 ^cd^	63.5 ± 1.9 ^f^	55.3 ± 0.0 ^c^	58.8 ± 1.4 ^bc^	48.2 ± 1.1 ^bc^	45.8 ± 0.0 ^d^	72.9 ± 1.7 ^b^	68.2 ± 2.2 ^d^
Pyrimethanil	71.1 ± 2.1 ^b^	72.8 ± 0.3 ^d^	65.9 ± 0.4 ^b^	72.3 ± 0.4 ^a^	50.7 ± 0.9 ^b^	71.3 ± 0.3 ^b^	77.6 ± 1.4 ^a^	70.7 ± 0.6 ^cd^

Note: Lowercase letters (^a^, ^b^, ^c^, ^d^, ^e^, ^f^, ^g^, ^h^, ^i^, ^j^, ^k^, ^l^, ^m^, ^n^ and ^o^) represent the results of one-way ANOVA for the same pathogen with different treatments at the same concentration (Duncan’s analysis, *p* ≤ 0.05). These letters indicate significant differences among the different groups, with ‘^a^’ representing the highest and ‘^o^’ the lowest inhibition rate in descending order. ***F.g***, *Fusarium graminearum*; ***C.m***, *Colletotrichum musae*; ***P.c***, *Phytophthora capsici*; ***F.o***, *Fusarium oxysporum*; ***C.s***, *Colletotrichum siamense*; ***P.o***, *Pyricularia oryae*; ***R.s***, *Rhizoctonia solani*; ***B.c***, *Botrytis cinerea.*

**Table 5:** EC_50_ (μg/mL) value of compounds against plant pathogens.

Compound	Phytopathogena	EC_50_	Toxic Regression Equation	R^2^
C2	*B.c*	17.87	y = 3.30x − 4.07	0.993
C3	*R.s*	11.19	y = 4.98x − 5.25	0.953
D1	*F.g*	5.56	y = 1.67x − 1.24	0.983
	*C.m*	3.85	y = 2.33x − 1.34	0.961
	*P.c*	29.01	y = 1.58x − 2.35	0.987
	*P.o*	6.59	y = 3.55x − 2.91	0.997
	*R.s*	7.66	y = 4.19x − 3.70	0.995
	*B.c*	2.98	y = 1.82x − 0.85	0.957
D2	*P.o*	7.53	y = 2.96x − 2.64	0.987
	*B.c*	3.19	y = 2.67x − 1.33	0.974
D3	*P.o*	12.59	y = 1.90x − 2.09	0.987
	*B.c*	9.15	y = 6.06x − 5.82	0.984
D4	*R.s*	37.17	y = 0.50x + 3.15	0.9548
D10	*C.m*	15.04	y = 2.21x − 2.61	0.973
	*P.o*	12.42	y = 3.13x − 3.43	0.991
	*B.c*	19.93	y = 1.48x − 1.93	0.975
D11	*P.o*	7.72	y = 3.75x − 3.44	0.978
	*B.c*	16.42	y = 1.71x − 2.09	0.975
D12	*R.s*	1.32	y = 1.70x − 0.22	0.990

Note: F.g, Fusarium graminearum; C.m, Colletotrichum musae; P.c, Phytophthora capsici; P.o, Pyricularia oryae; R.s, Rhizoctonia solani; B.c, Botrytis cinerea.

**Table 6:** Efficacy of compounds D1 and pyrimethanil on tomato fruit against *B. cinerea* in vivo.

Treatment		Lesion Area (cm^3^)	Inhibition Rate (%)
D1	100 μg/mL	10.71 ± 0.04 ^b^	66.16 ± 0.14 ^d^
	200 μg/mL	1.06 ± 0.34 ^e^	96.65 ± 1.06 ^a^
Pyrimethanil	100 μg/mL	7.11 ± 0.15 ^c^	77.53 ± 0.51 ^c^
	200 μg/mL	3.07 ± 0.31 ^d^	90.30 ± 1.01 ^b^
Solvent control		31.41 ± 0.77 ^a^	-
Blank control		31.65 ± 0.13 ^a^	-

Note: Lowercase letters represent one-way ANOVA results between different groups at the same time point (Duncan analysis *p* ≤ 0.05). ^a^, ^b^, ^c^, ^d^, and ^e^ indicate significant differences between different groups, and ^a^, ^b^, ^c^, ^d^, and ^e^ are labeled in descending order of inhibition rate.

## Data Availability

Data has been stored in the library repository of Hainan University, Haikou, China.

## Supplementary Materials

The following supporting information can be downloaded at: https://www.mdpi.com/article/10.3390/ijms25179209/s1.
