# Supplementary material for: Design, Synthesis, and Nematocidal Evaluation of Waltherione A Derivatives: Leveraging a Structural Simplification Strategy
Source: Int J Mol Sci. 2024 Aug 25;25(17):9209. doi: 10.3390/ijms25179209 (PMC11394673; doi:10.3390/ijms25179209)
Supplement: Supplementary file 1 [file ijms-25-09209-s001.zip › ijms-3155996-supplementary.pdf]

Design, Synthesis, and Nematocidal Evaluation of Waltherione A Derivatives Leveraging A Structural Simplification Strategy

Zhan Hu, Bin Yang, Shuai Zheng, Ke Zhao, Kaifeng Wang, Ranfeng Sun *

Key Laboratory of Green Prevention and Control of Tropical Agriculture and Forestry BioDisasters of Ministry of Education, School of Tropical Agriculture and Forestry, Hainan University, Haikou 570228, China.

(Supporting Information)

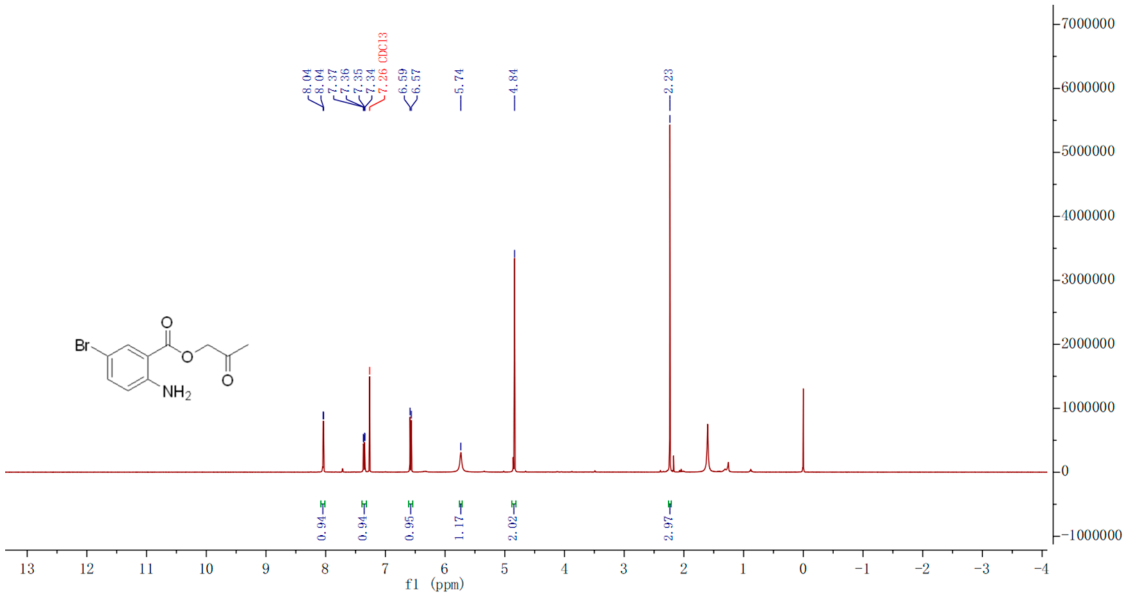

Figure S1. Compound B1 ^{1}H NMR

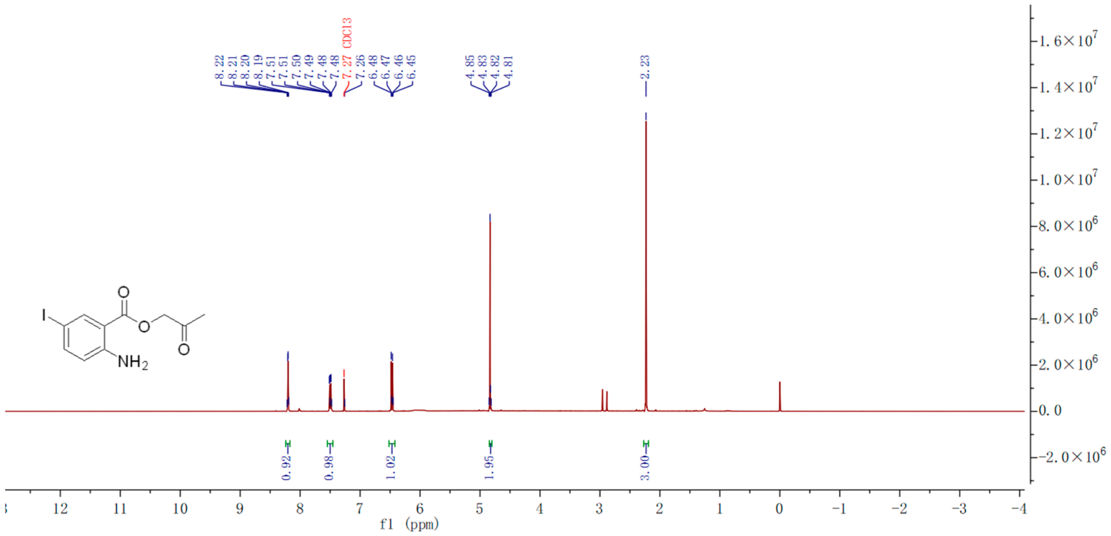

Figure S2. Compound B2 ^{1}H NMR

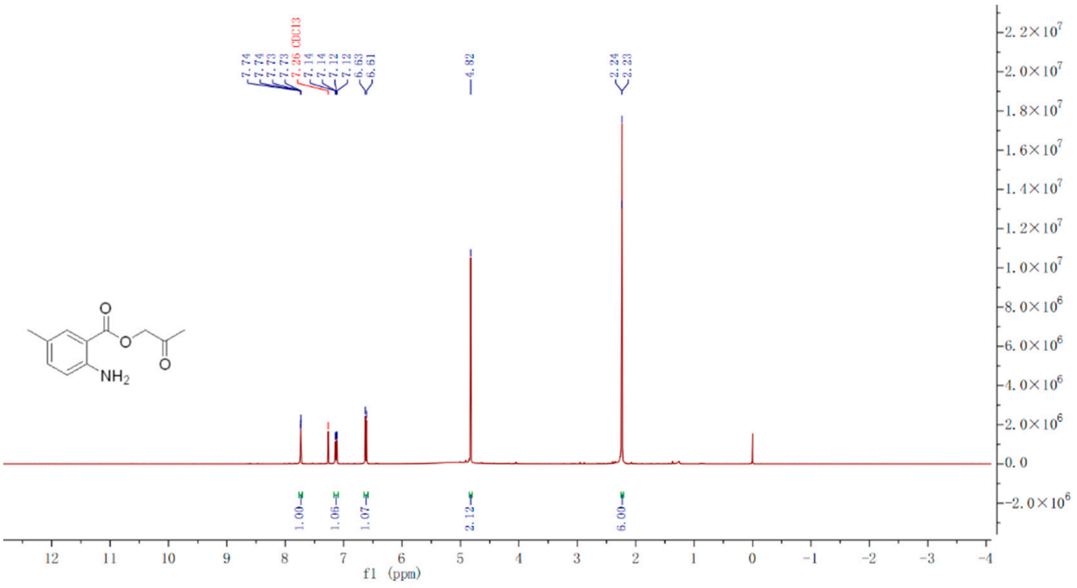

Figure S3. Compound B3 ^{1}H NMR

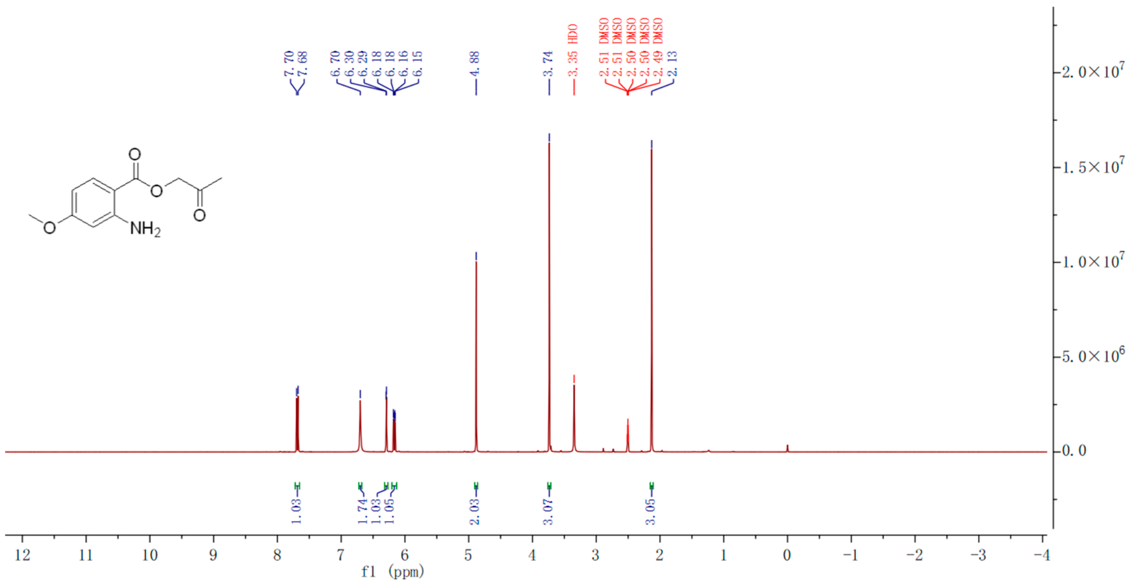

Figure S4. Compound B4 ^{1}H NMR

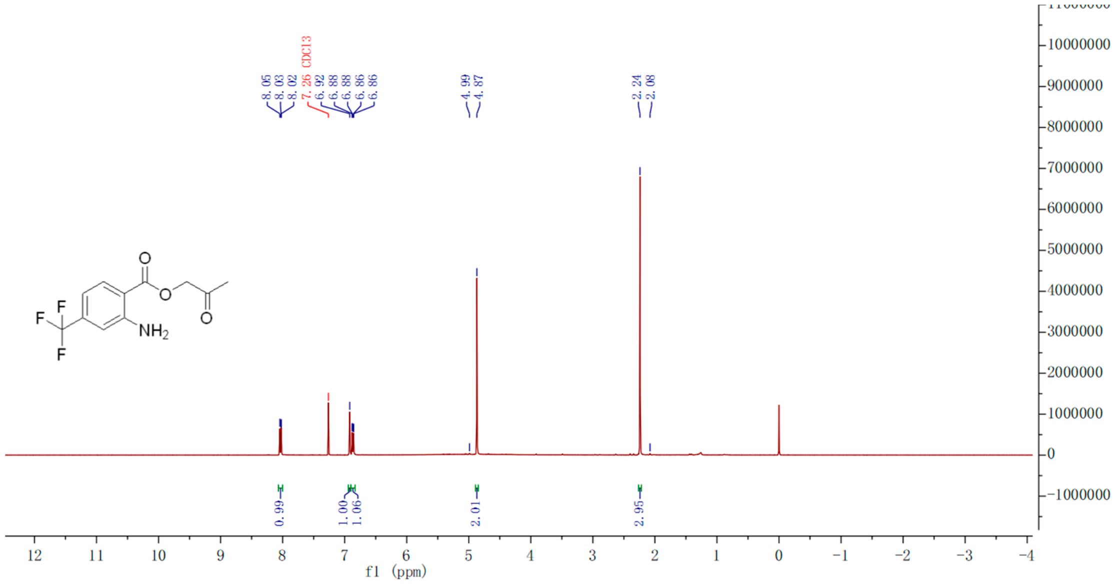

Figure S5. Compound B5 ^{1}H NMR

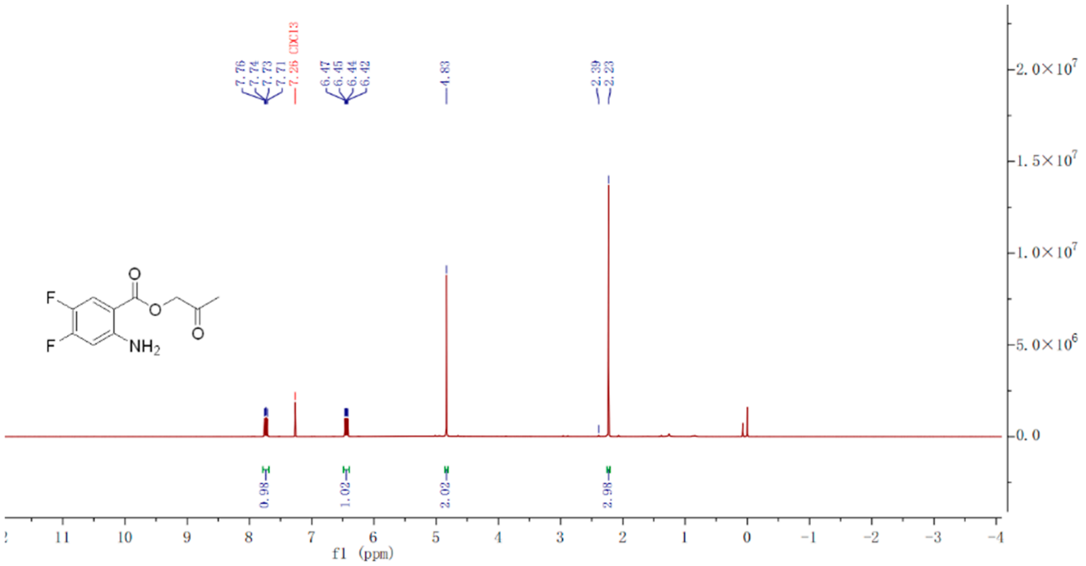

Figure S6. Compound B6 ^{1}H NMR

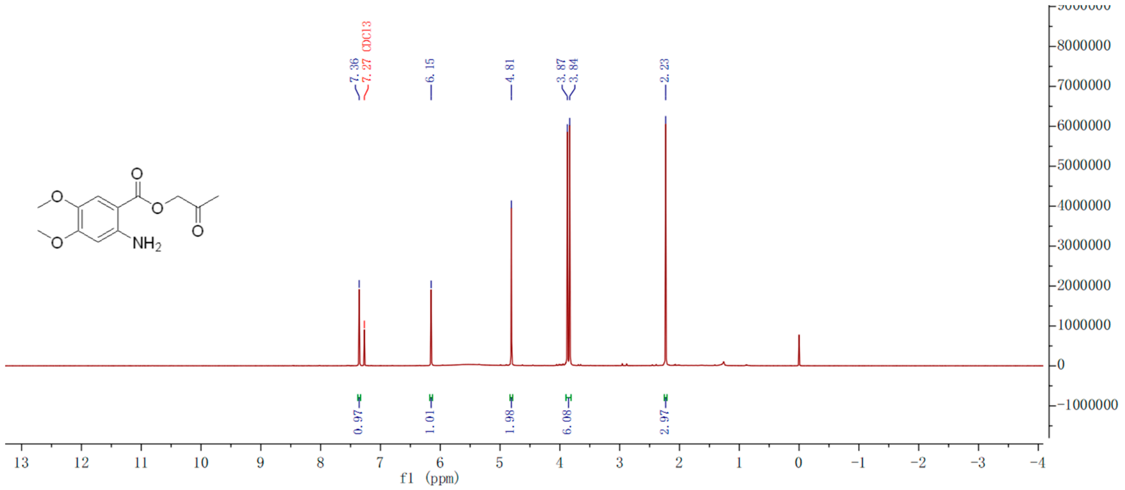

Figure S7. Compound B7 ^{1}H NMR

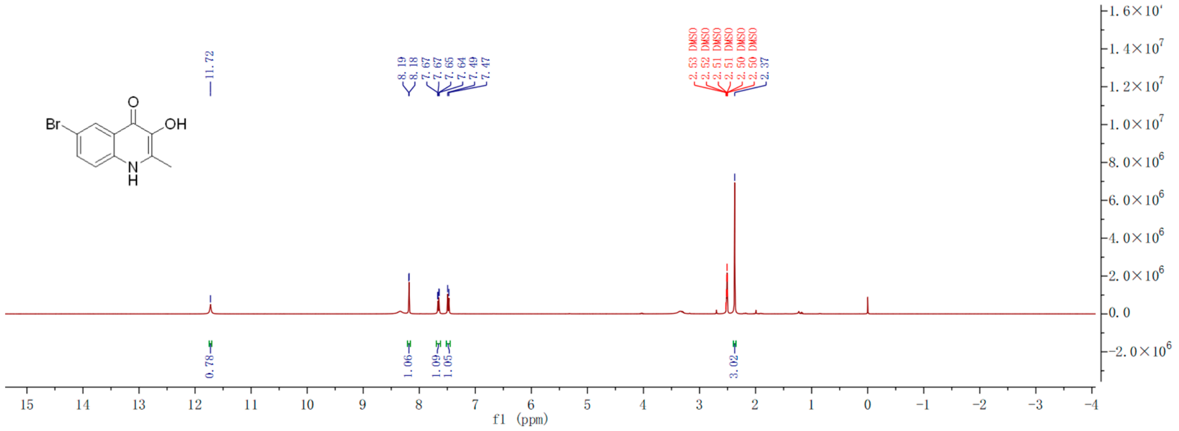

Figure S8. Compound C1 ^{1}H NMR

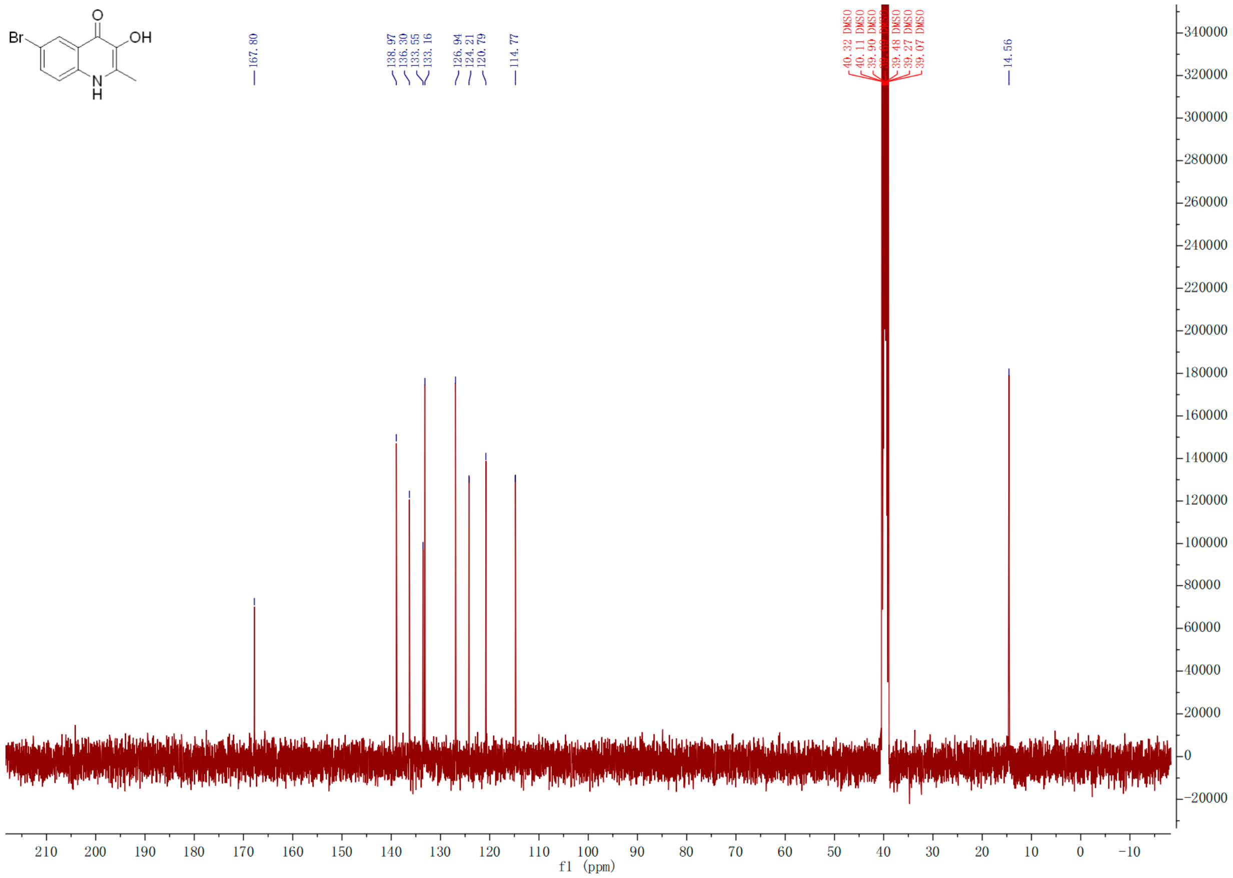

Figure S9. Compound C1 ^{13}C NMR

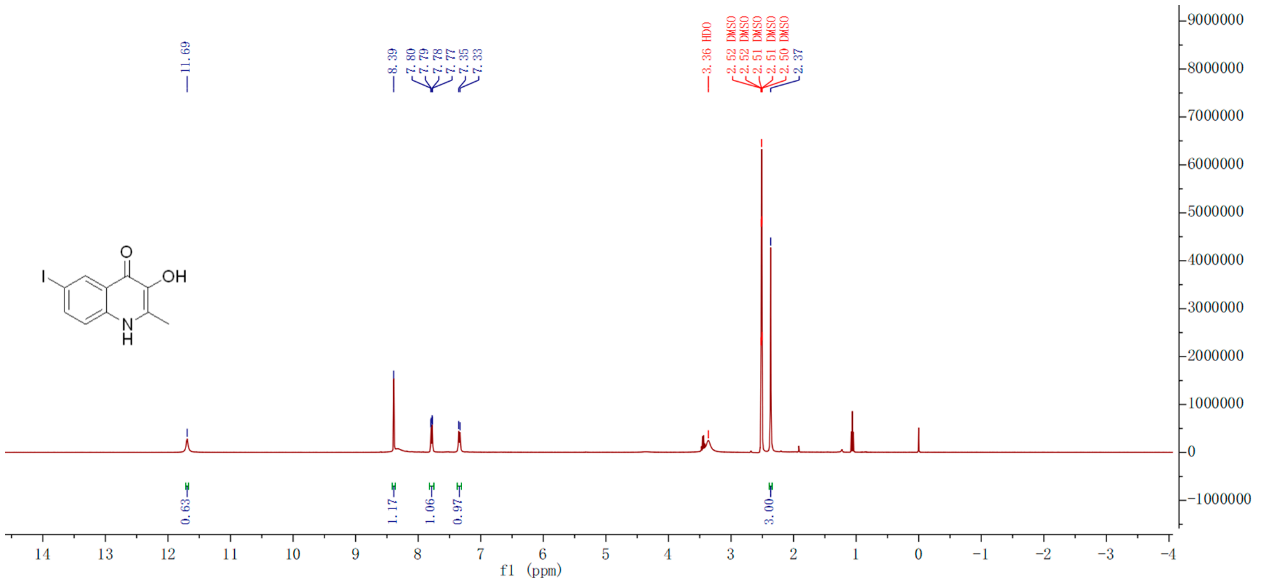

Figure S10. Compound C2 ^{1}H NMR

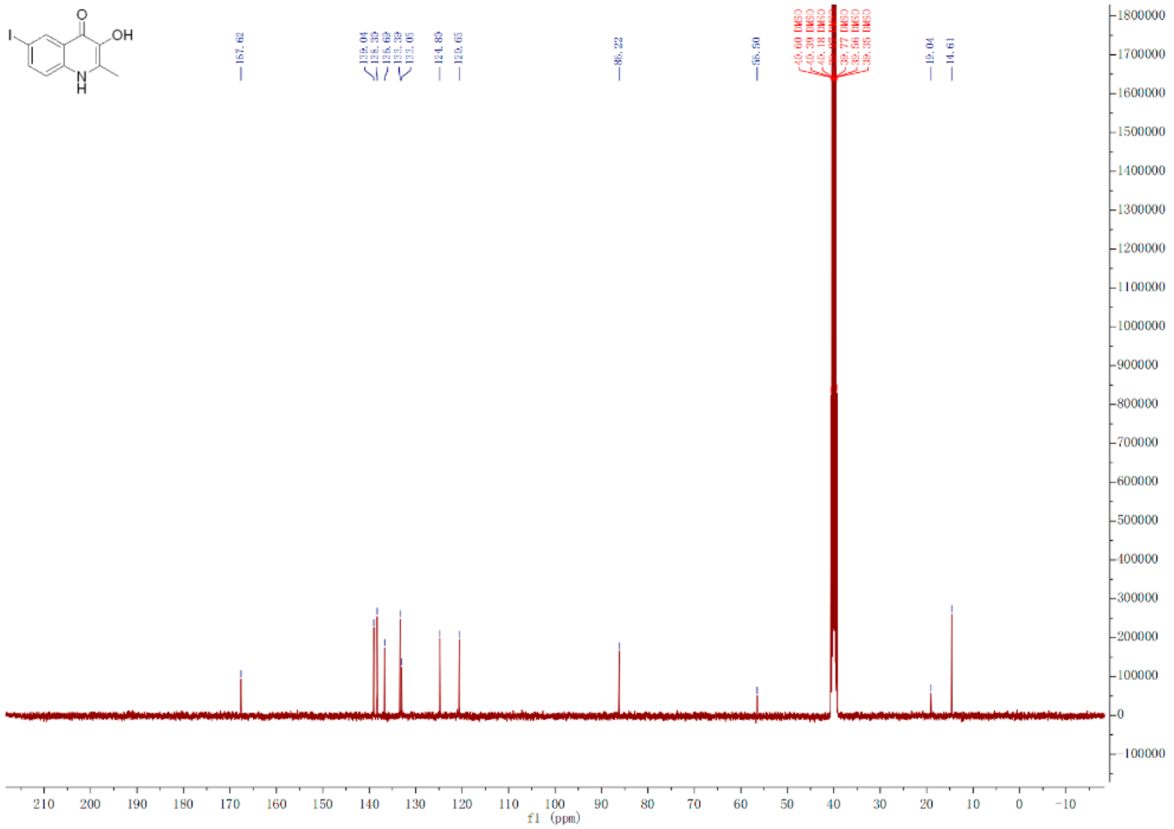

Figure S11. Compound C2 ^{13}C NMR

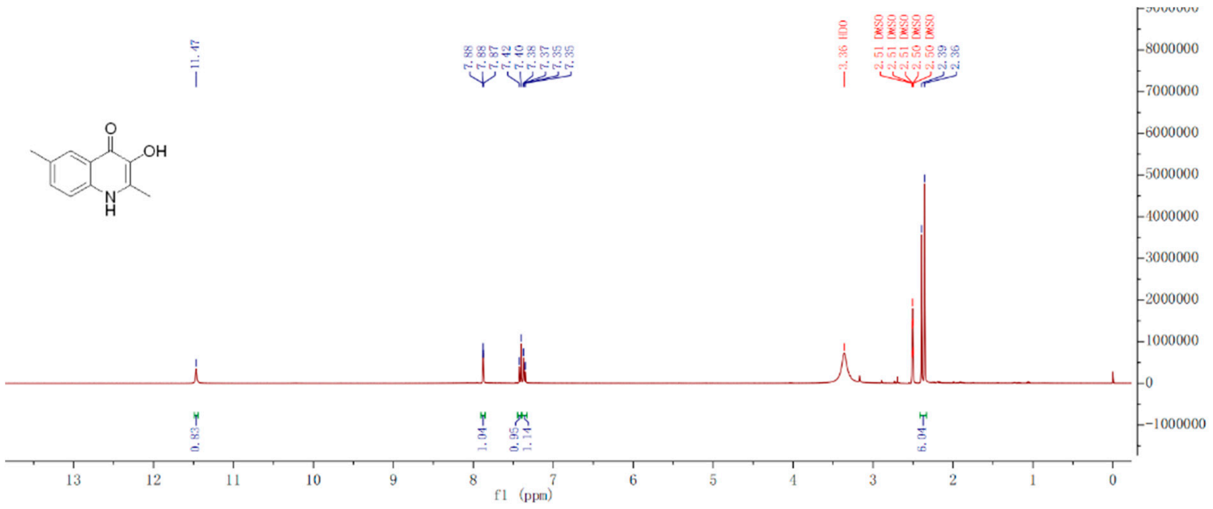

Figure S12. Compound C3 ^{1}H NMR

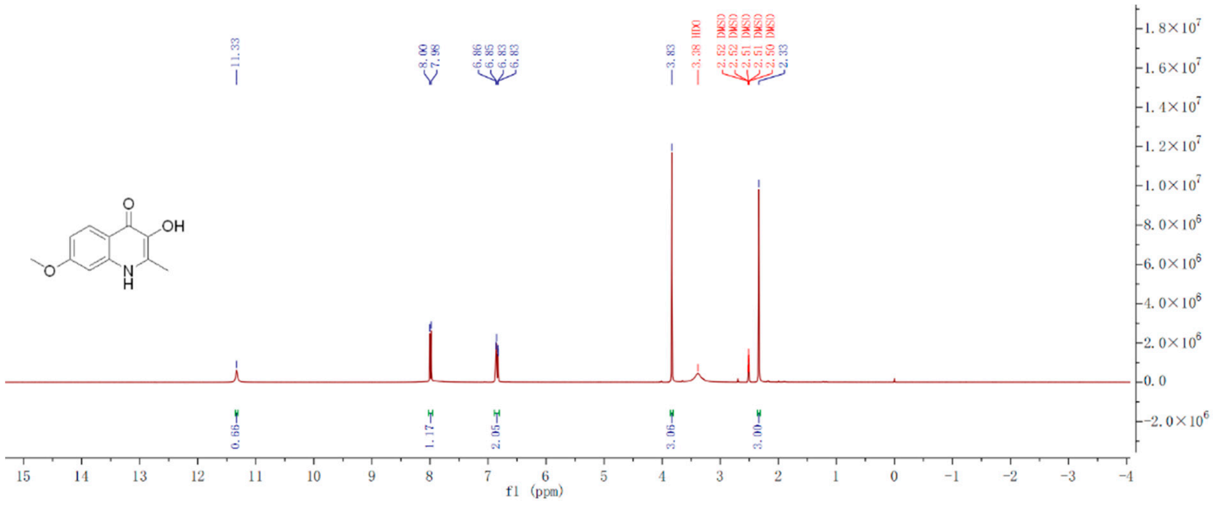

Figure S13. Compound C4 ^{1}H NMR

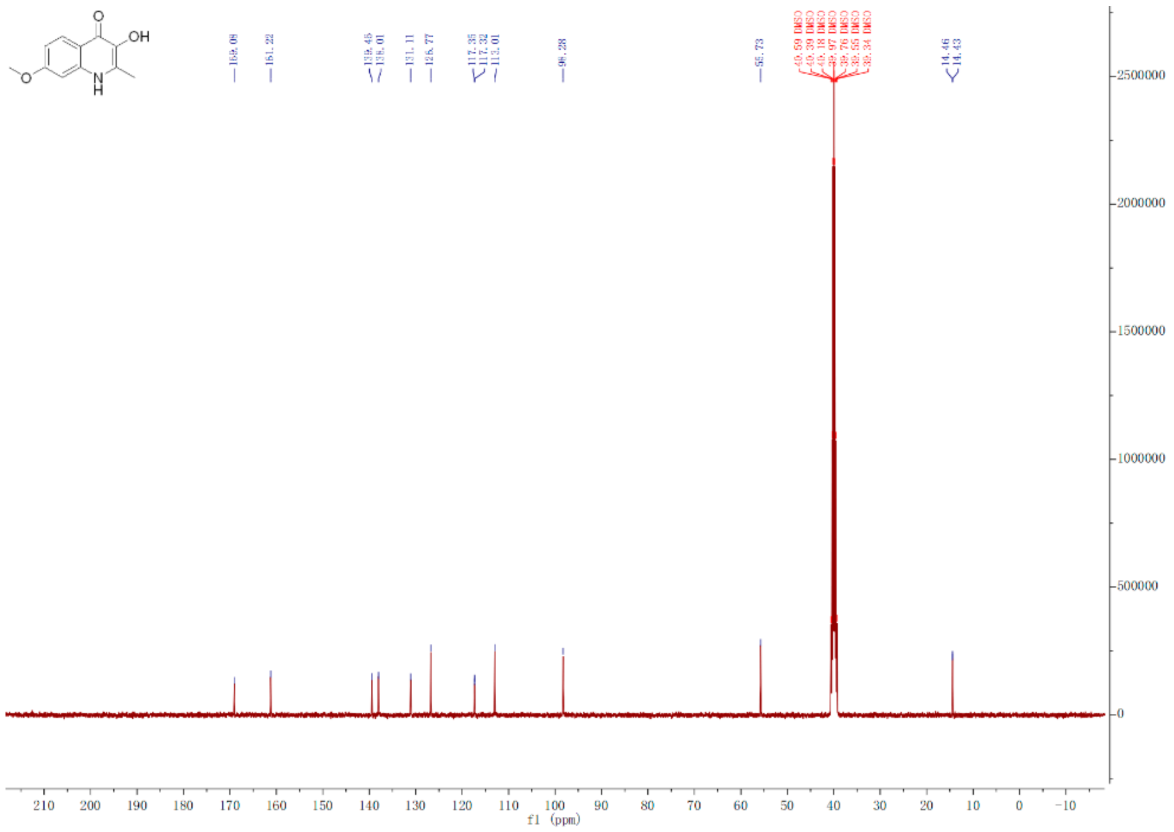

Figure S14. Compound C4 ^{13}C NMR

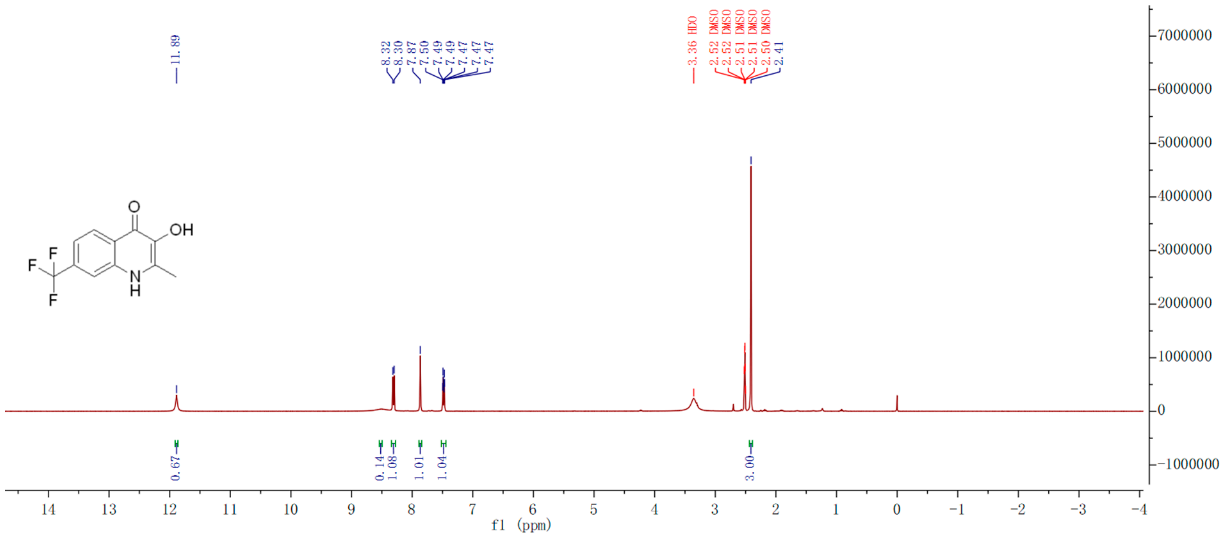

Figure S15. Compound C5 ^{1}H NMR

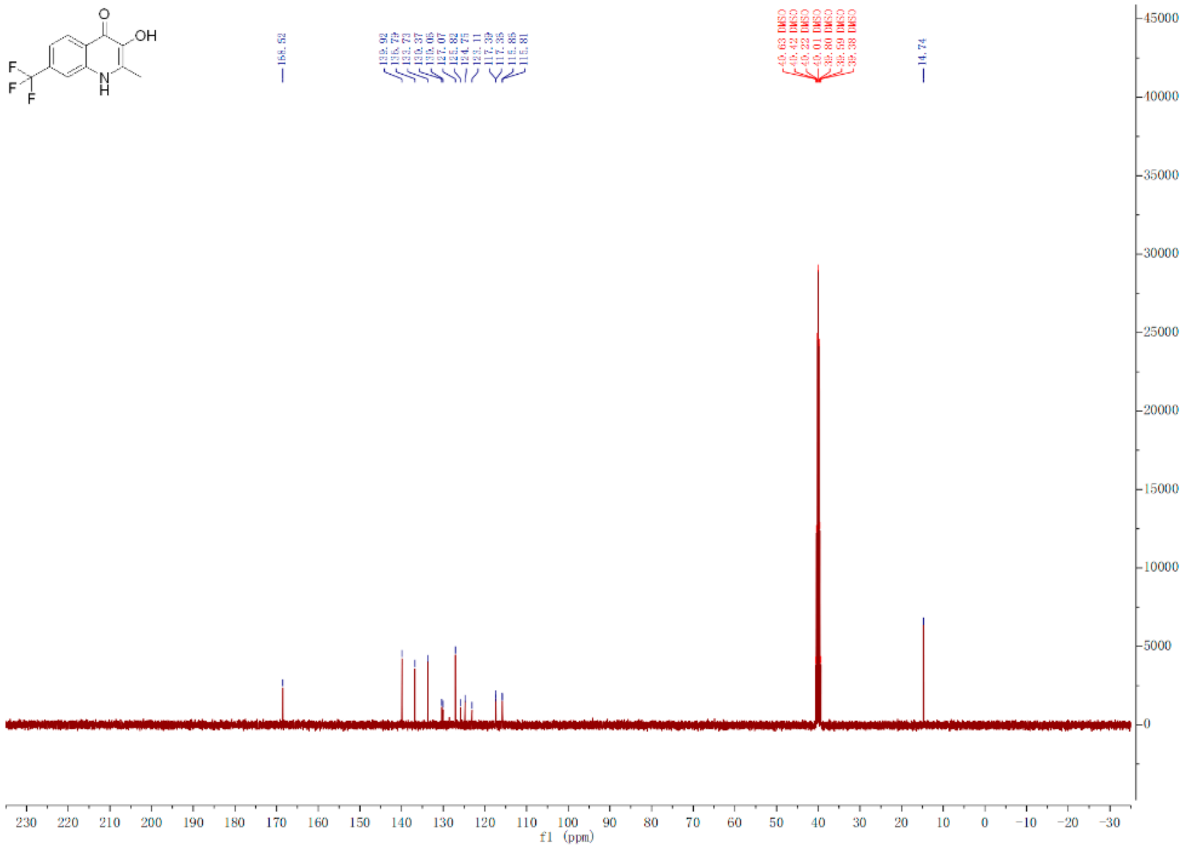

Figure S16. Compound C5 ^{13}C NMR

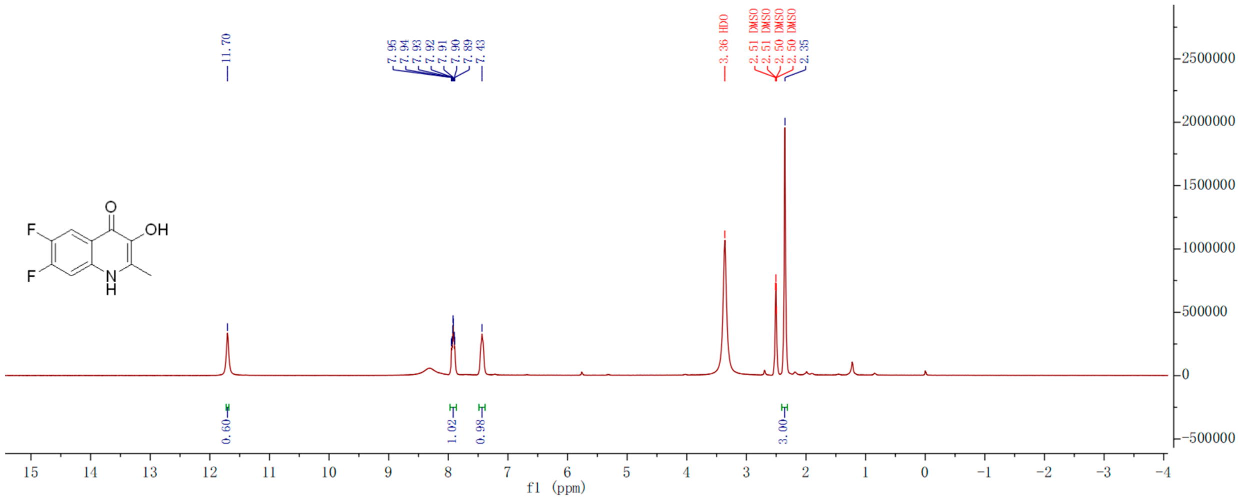

Figure S17. Compound C6 ^{1}H NMR

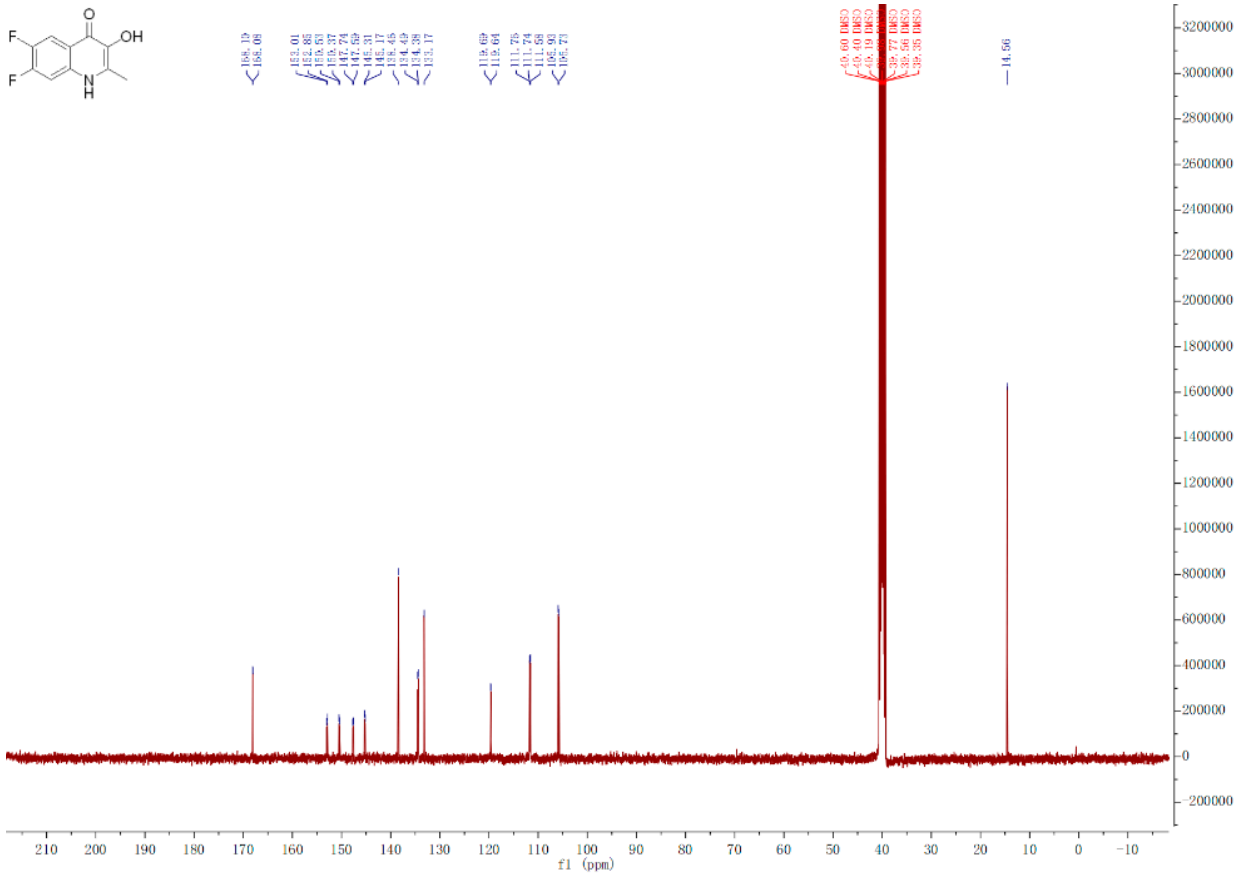

Figure S18. Compound C6 ^{13}C NMR

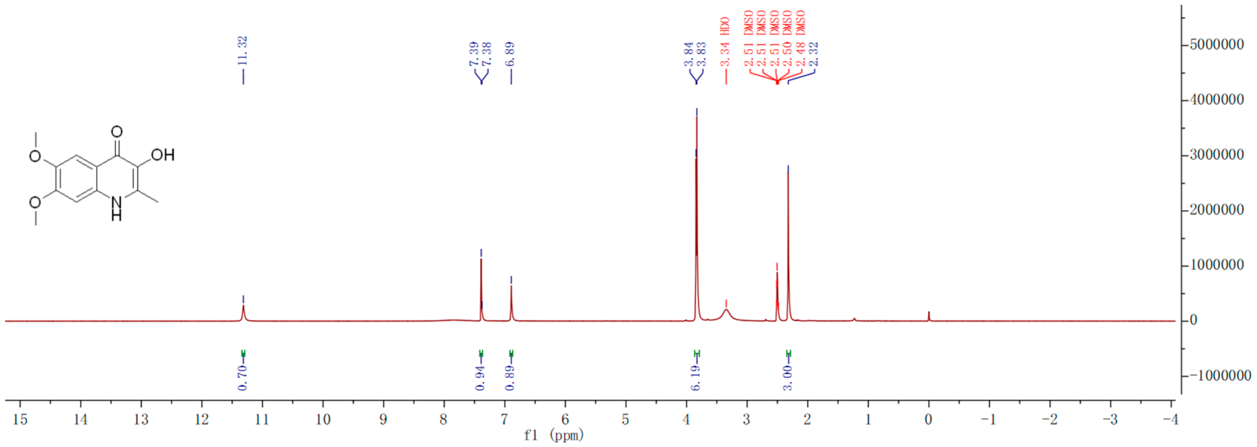

Figure S19. Compound C7 ^{1}H NMR

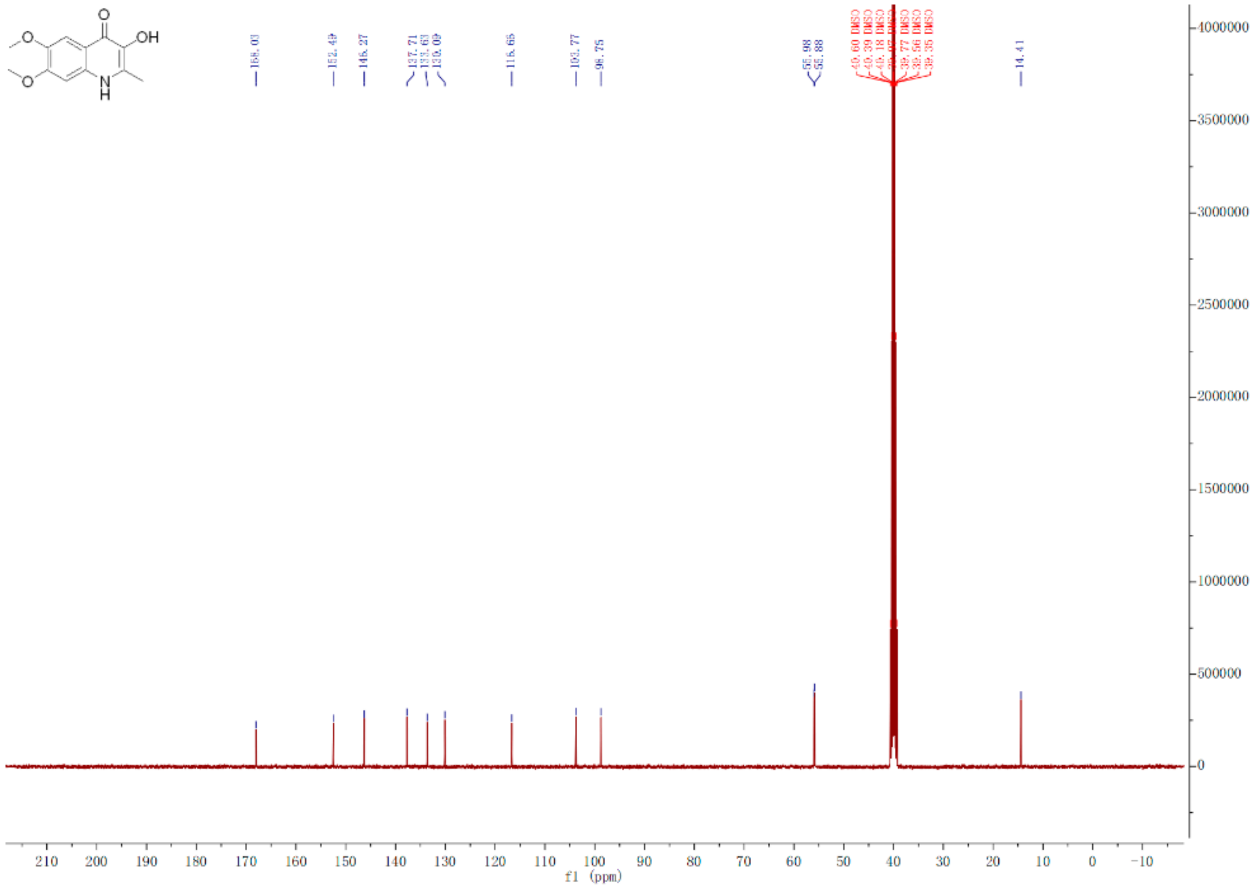

Figure S20. Compound C7 ^{13}C NMR

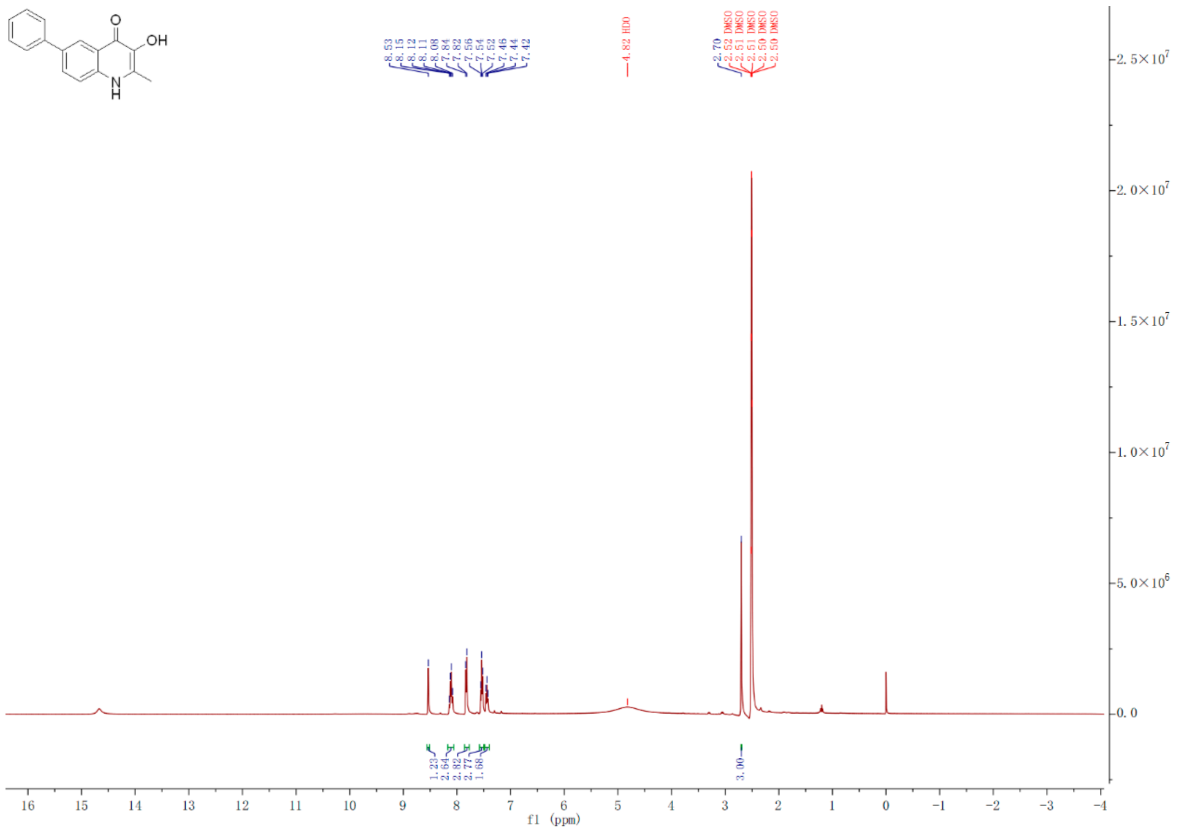

Figure S21. Compound C8 ^{1}H NMR

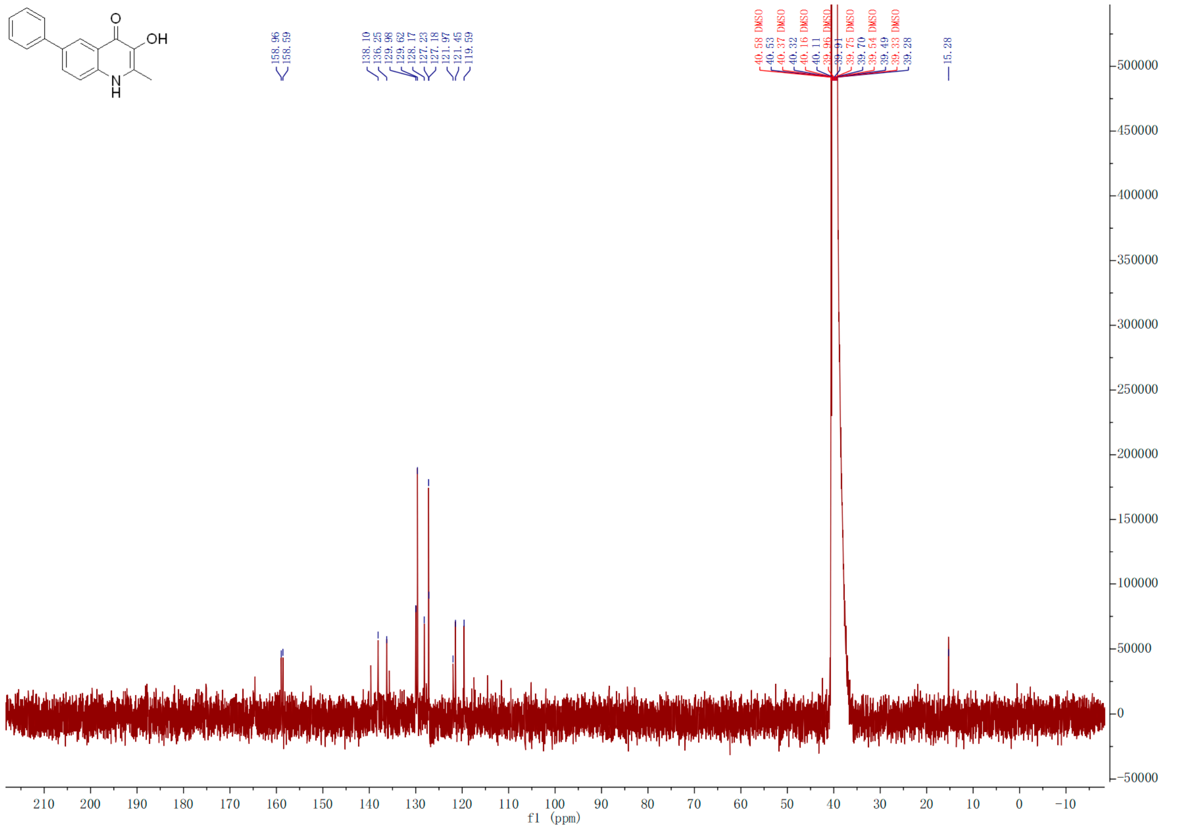

Figure S22. Compound C8 ^{13}C NMR

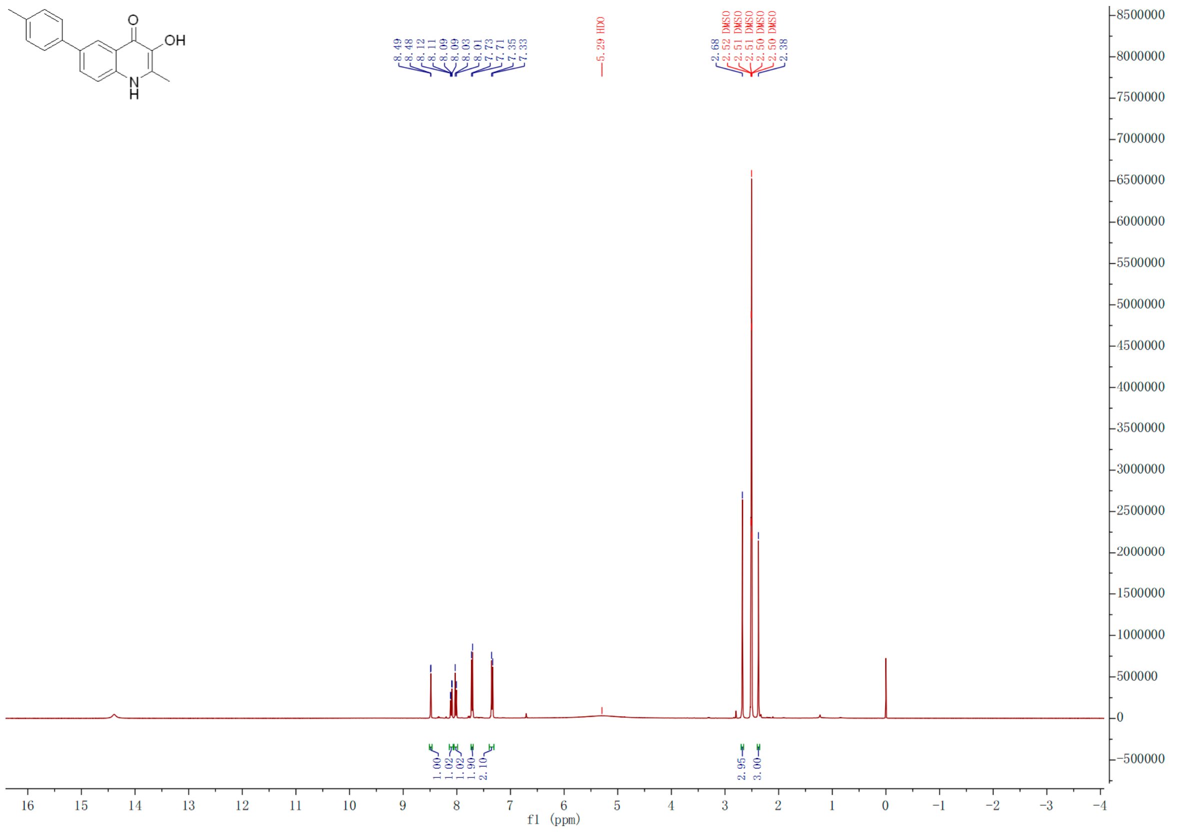

Figure S23. Compound C9 ^{1}H NMR

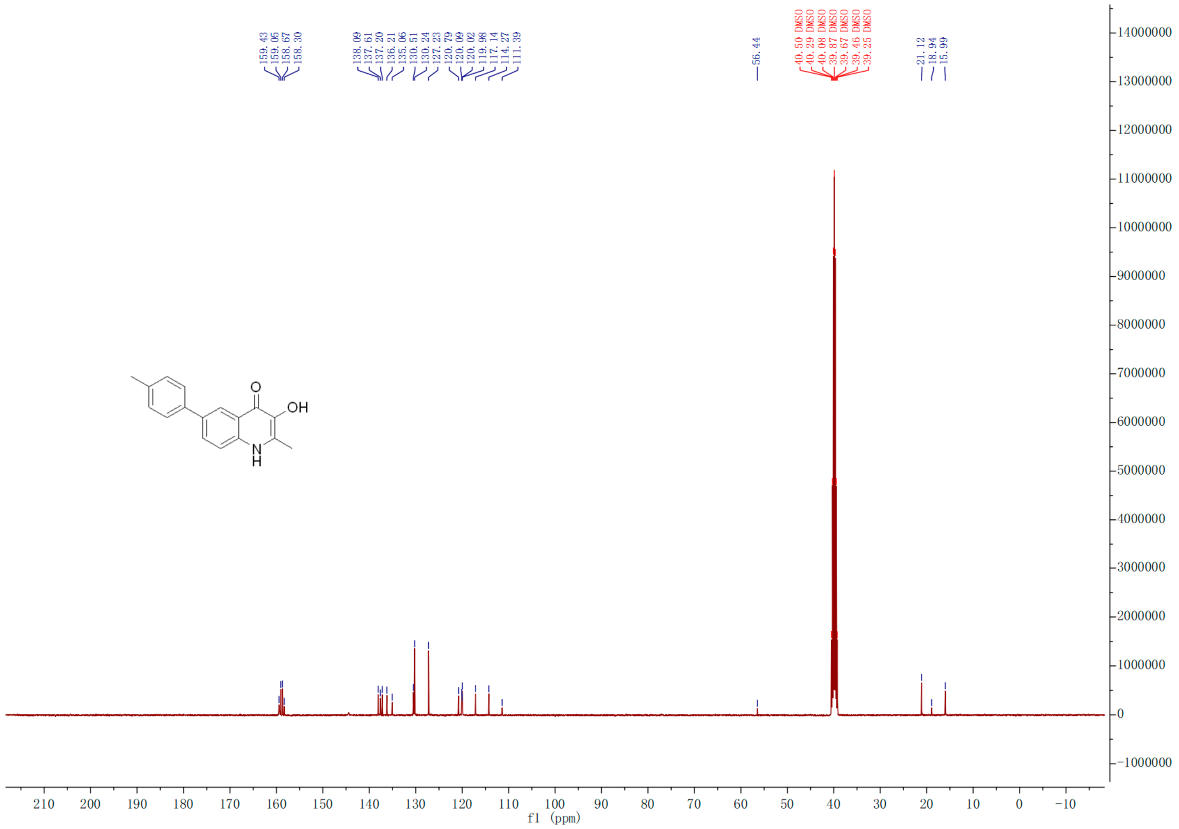

Figure S24. Compound C9 ^{13}C NMR

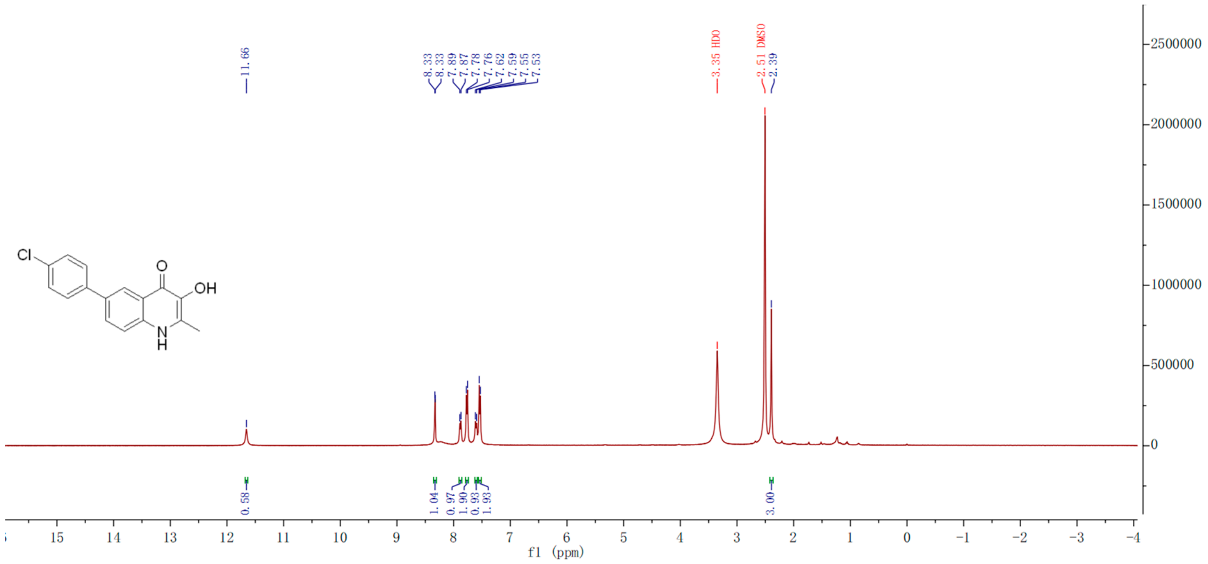

Figure S25. Compound C10 ^{1}H NMR

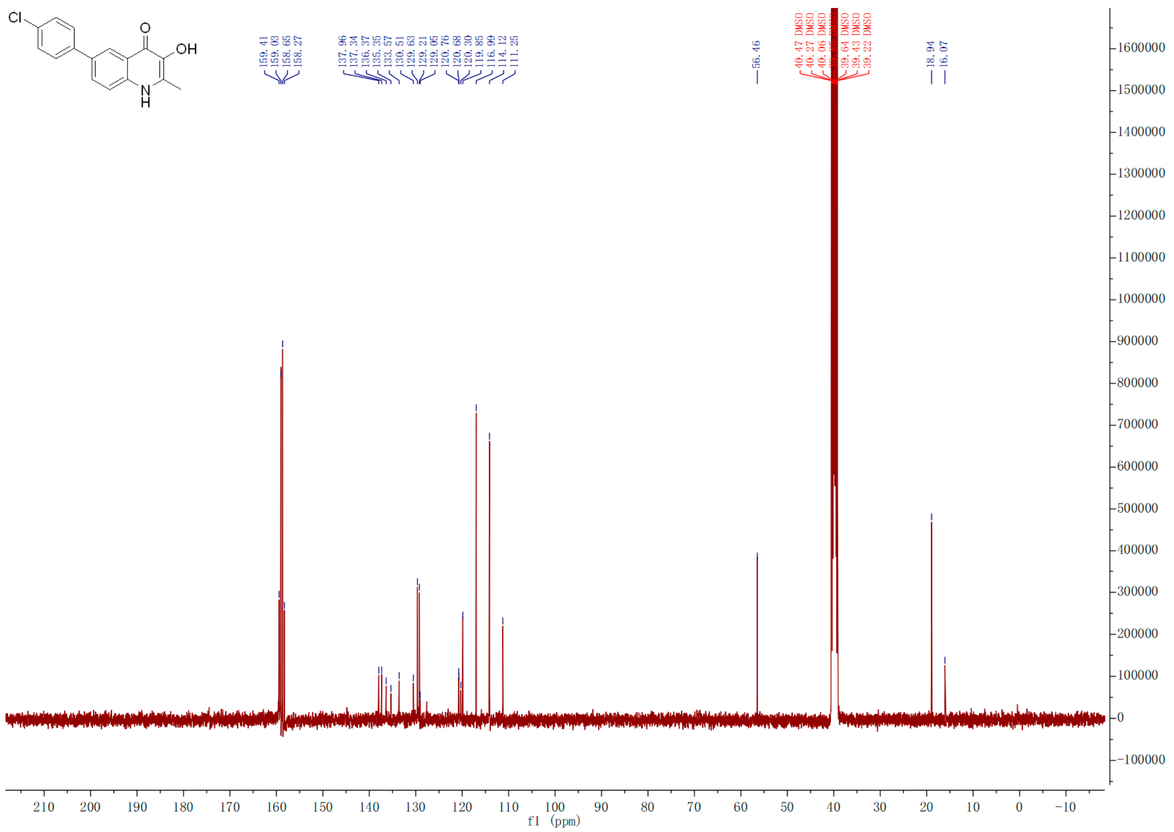

Figure S26. Compound C10 ^{13}C NMR

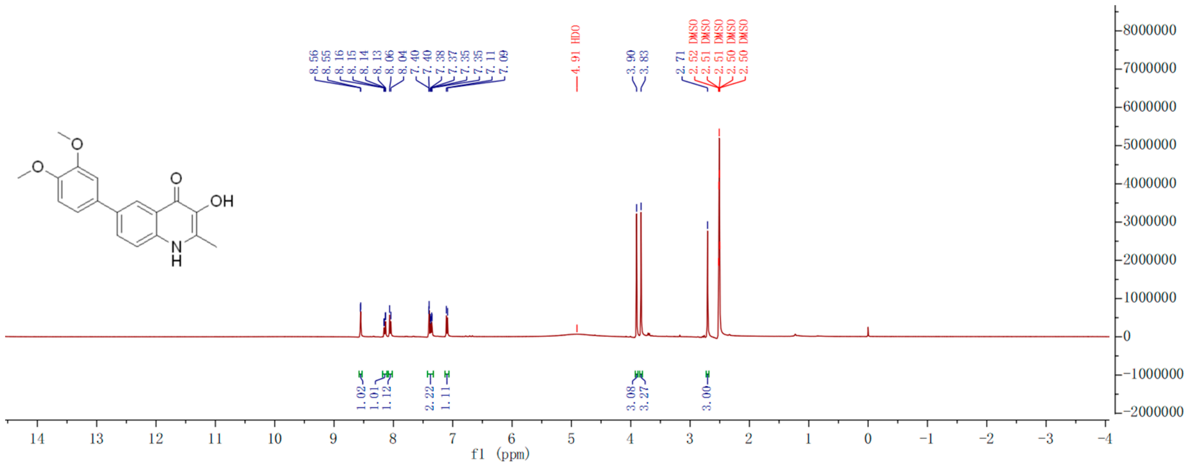

Figure S27. Compound C11 ^{1}H NMR

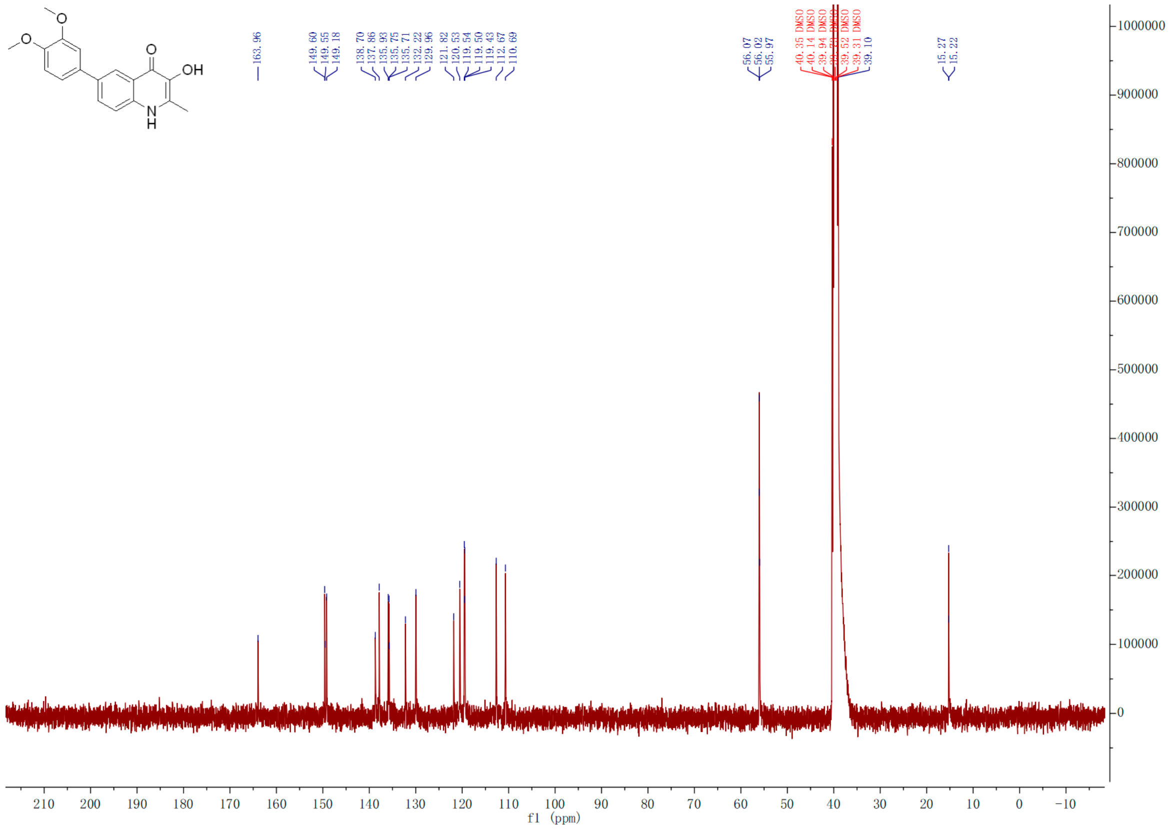

Figure S28. Compound C11 ^{13}C NMR

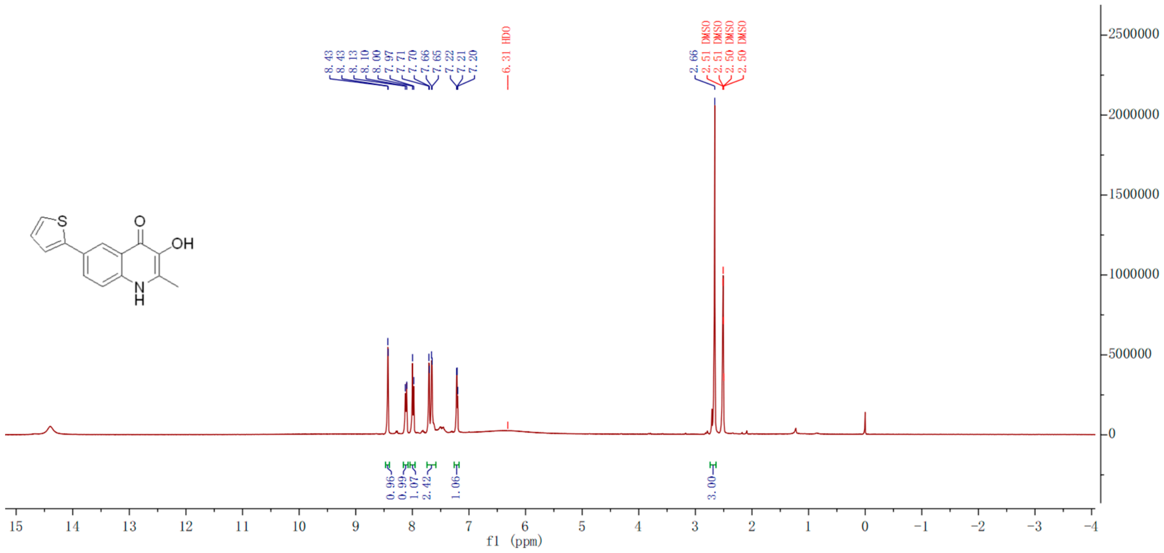

Figure S29. Compound C12 ^{1}H NMR

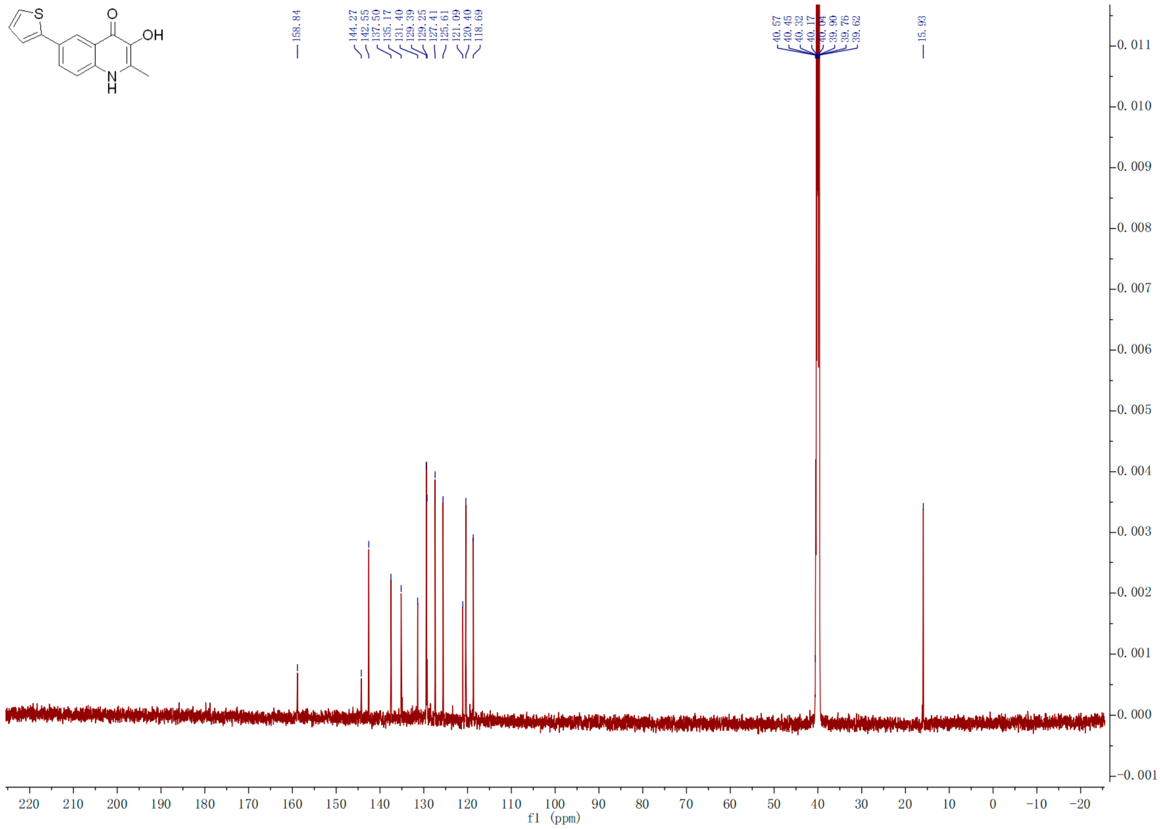

Figure S30. Compound C12 ^{13}C NMR

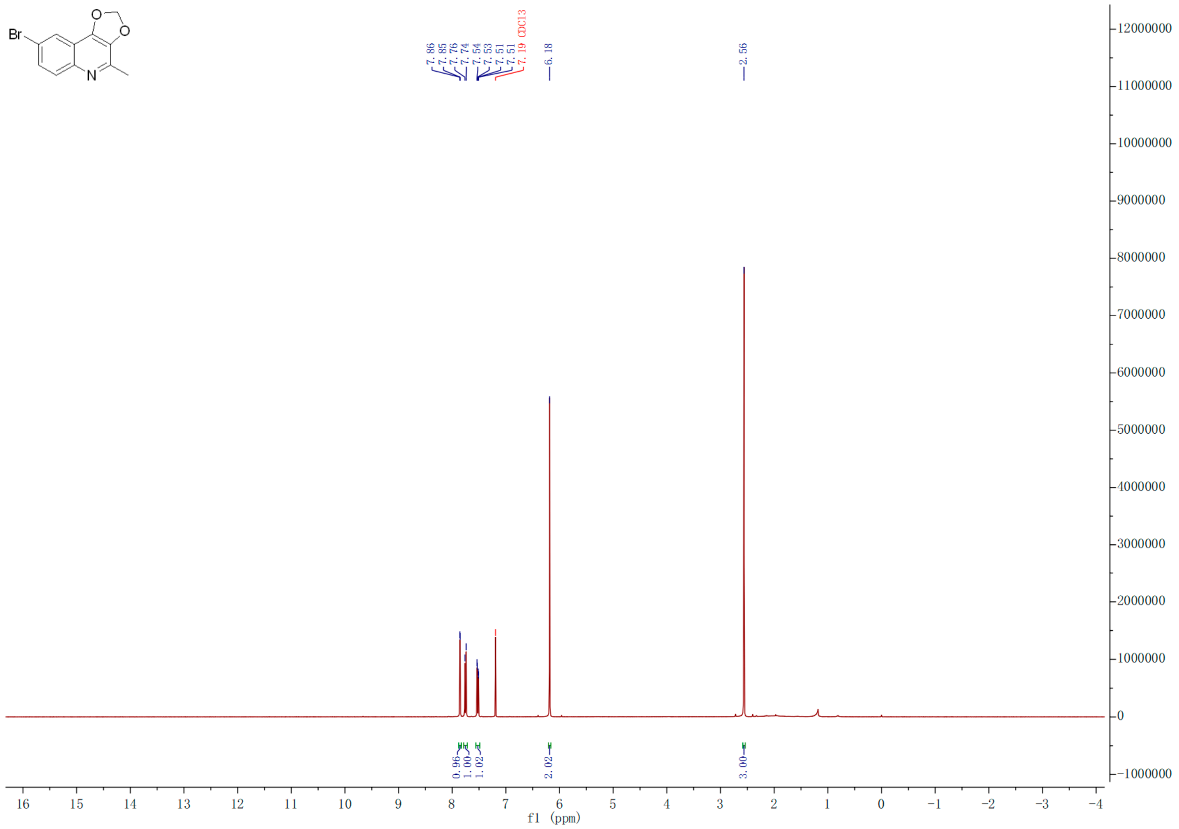

Figure S31. Compound D1 ^{1}H NMR

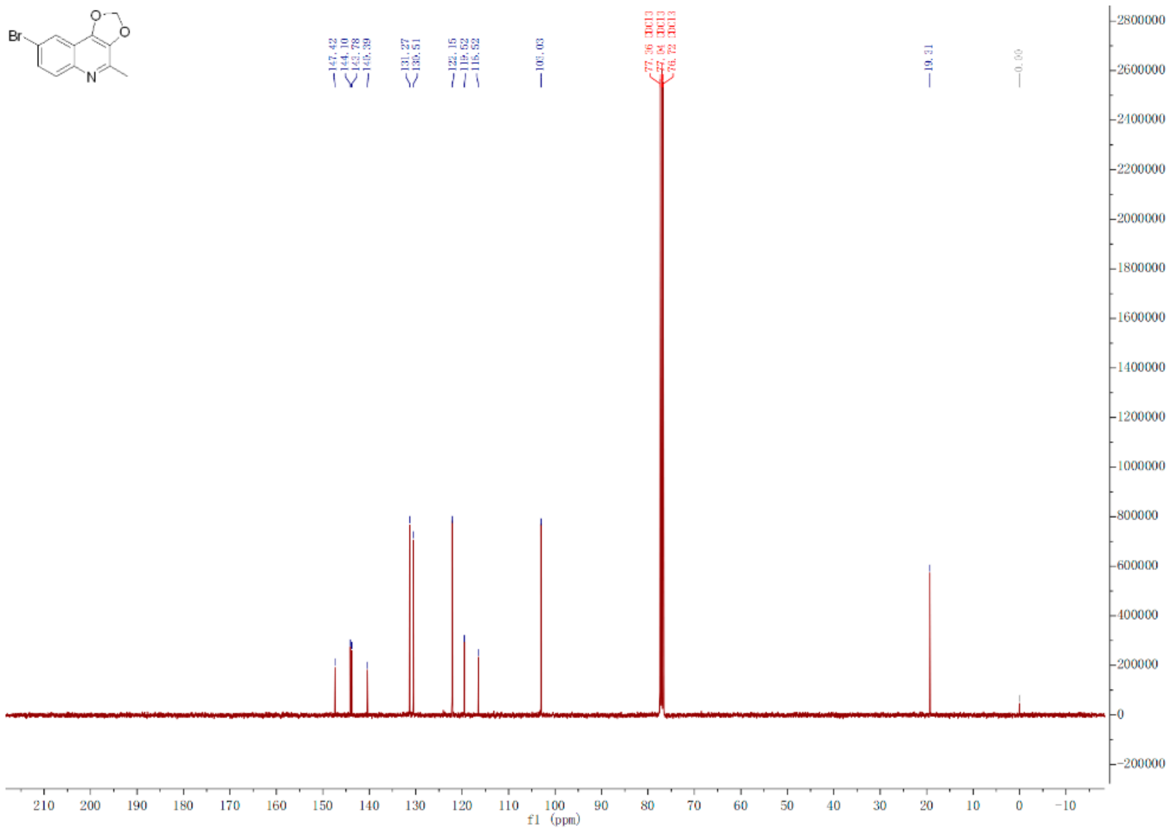

Figure S32. Compound D1 ^{13}C NMR

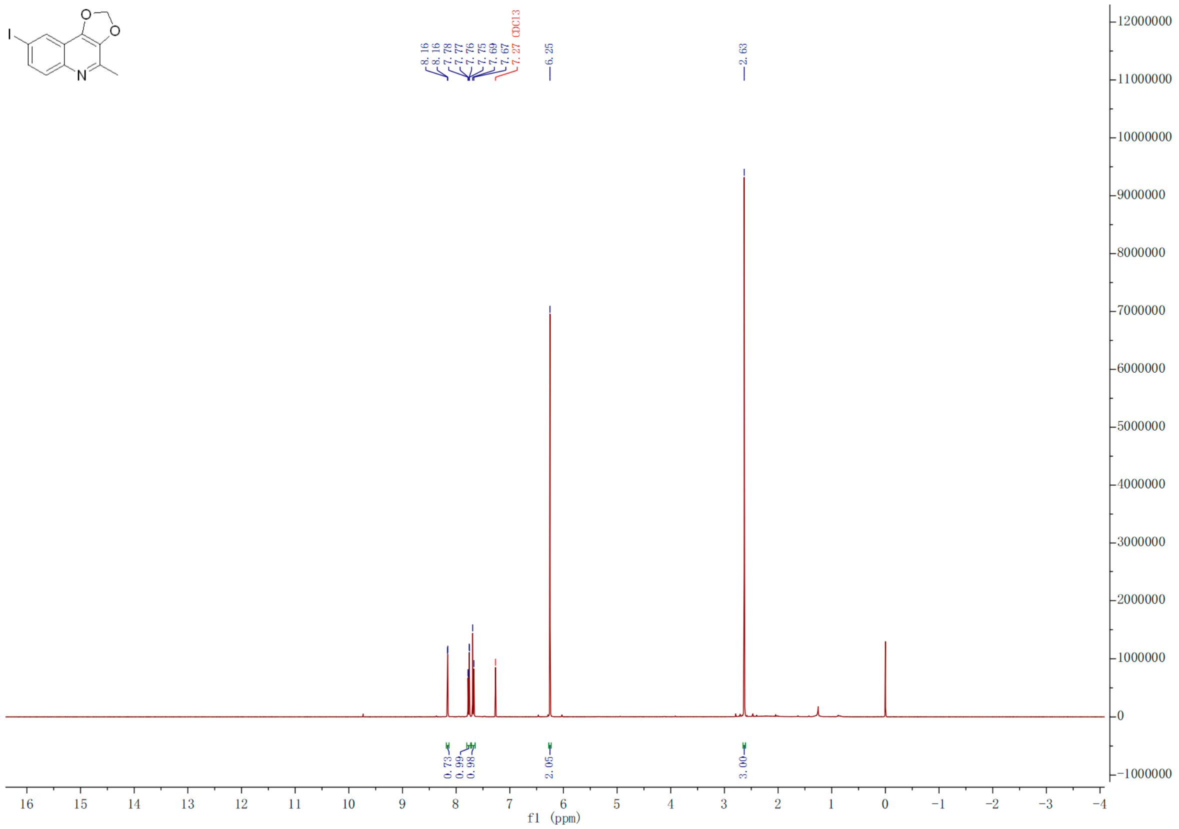

Figure S33. Compound D2 ¹H NMR

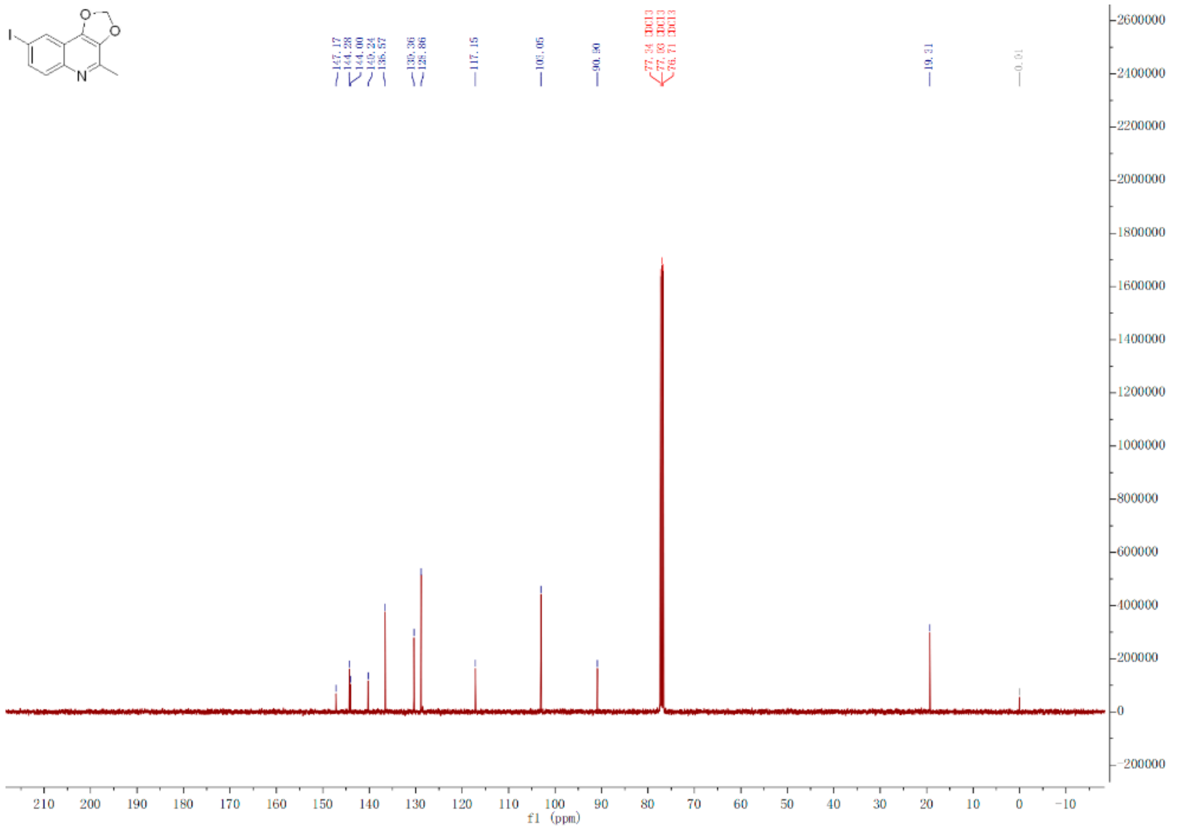

Figure S34. Compound D2 ¹³C NMR

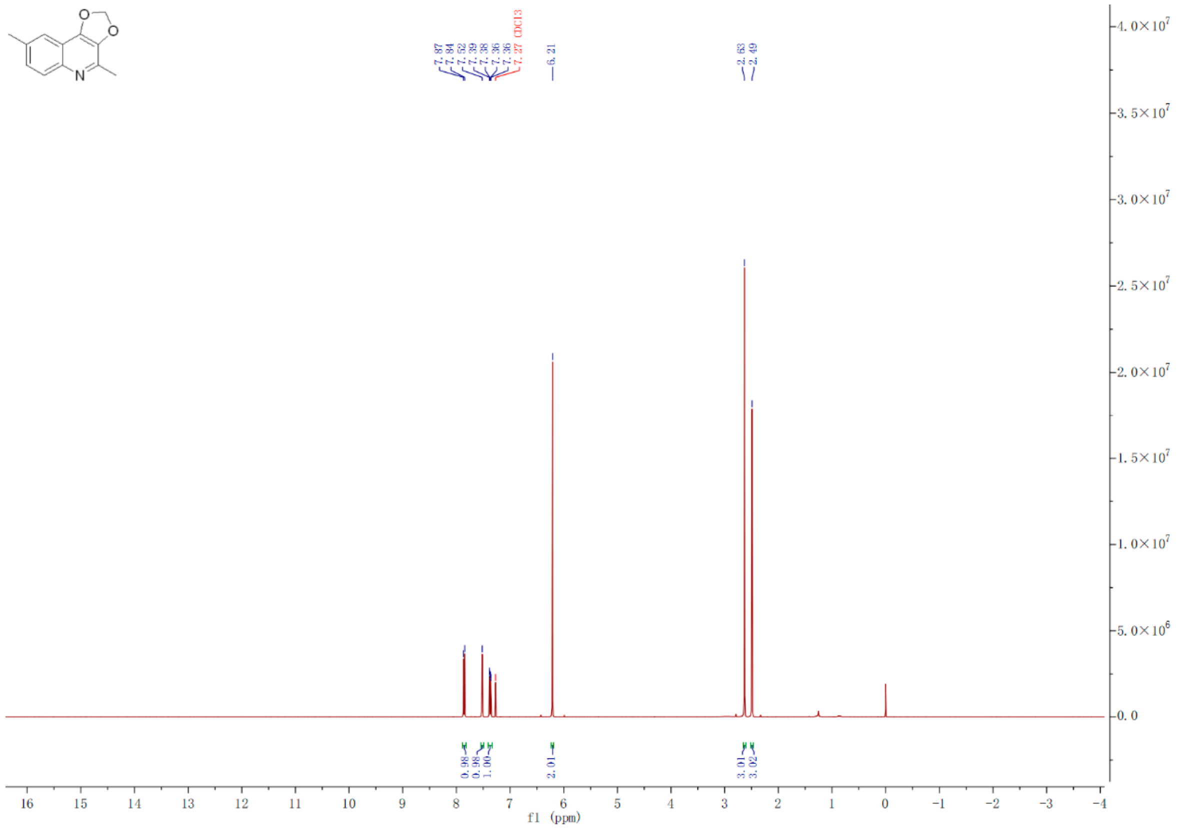

Figure S35. Compound D3 ^{1}H NMR

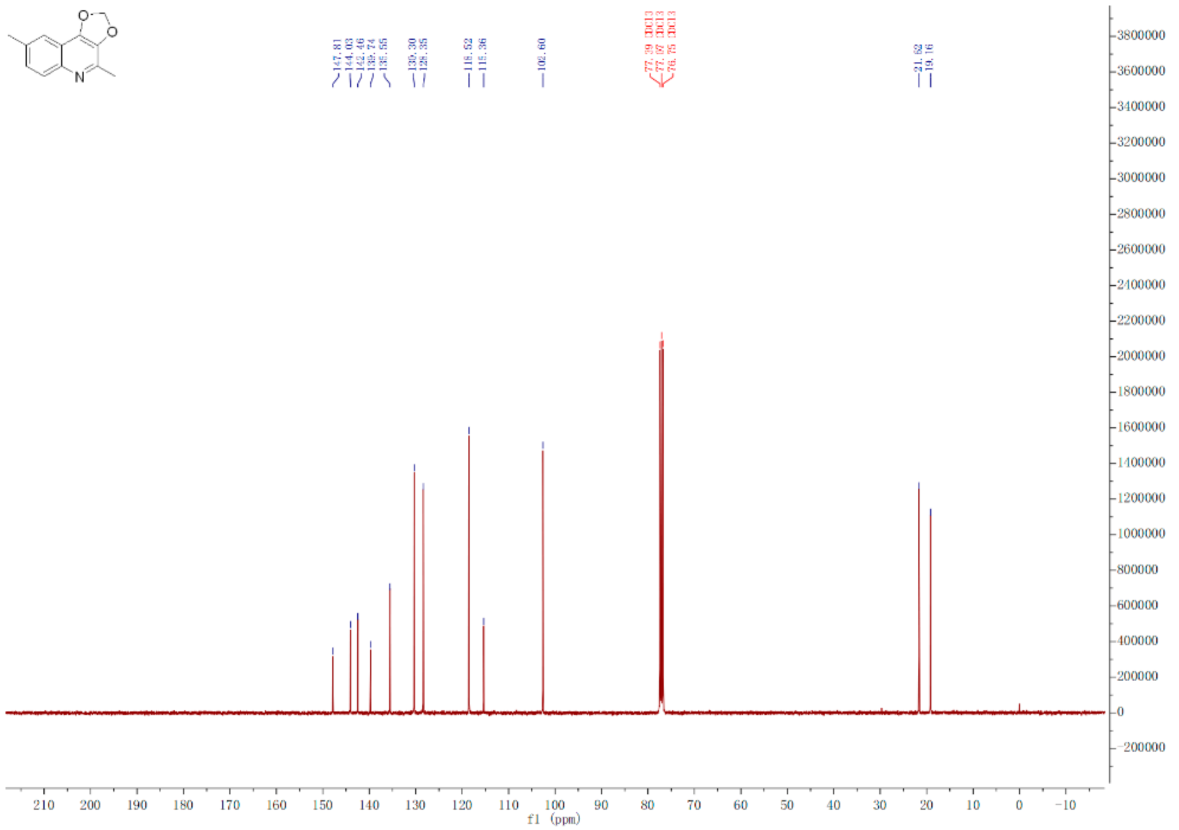

Figure S36. Compound D3 ^{13}C NMR

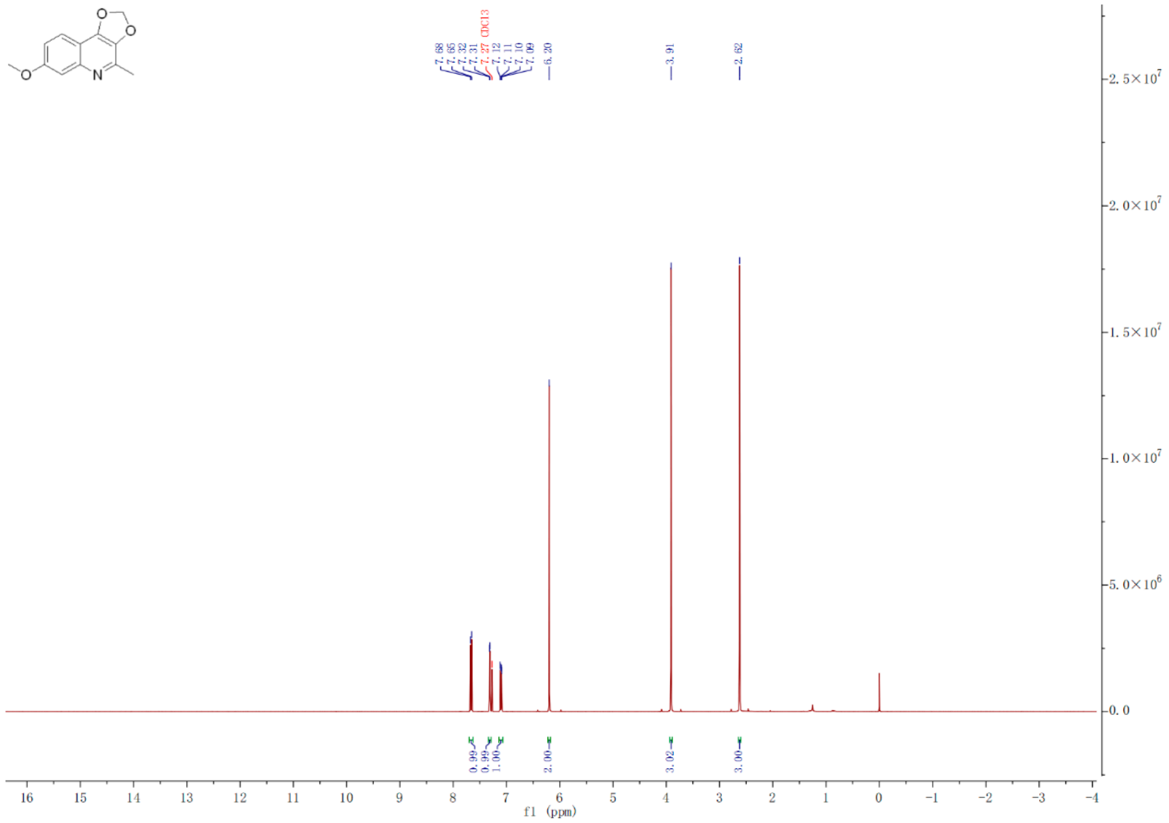

Figure S37. Compound D4 ^{1}H NMR

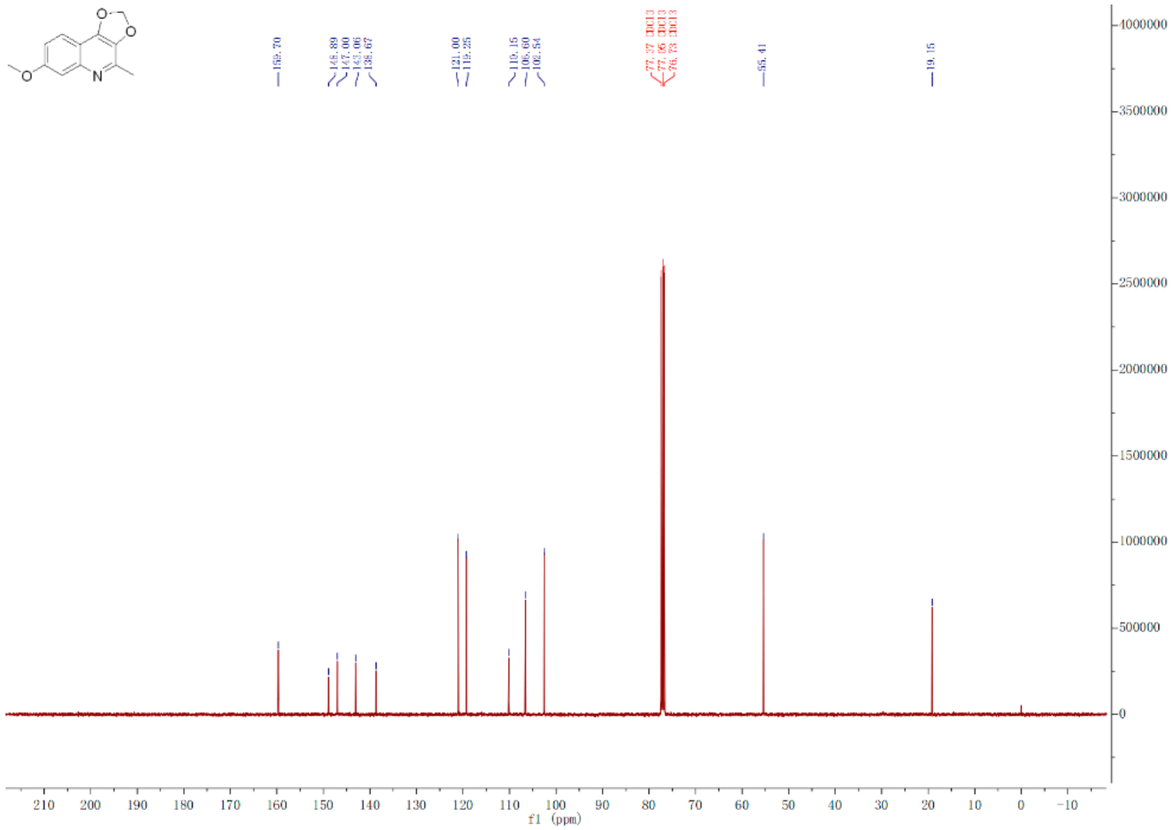

Figure S38. Compound D4 ^{13}C NMR

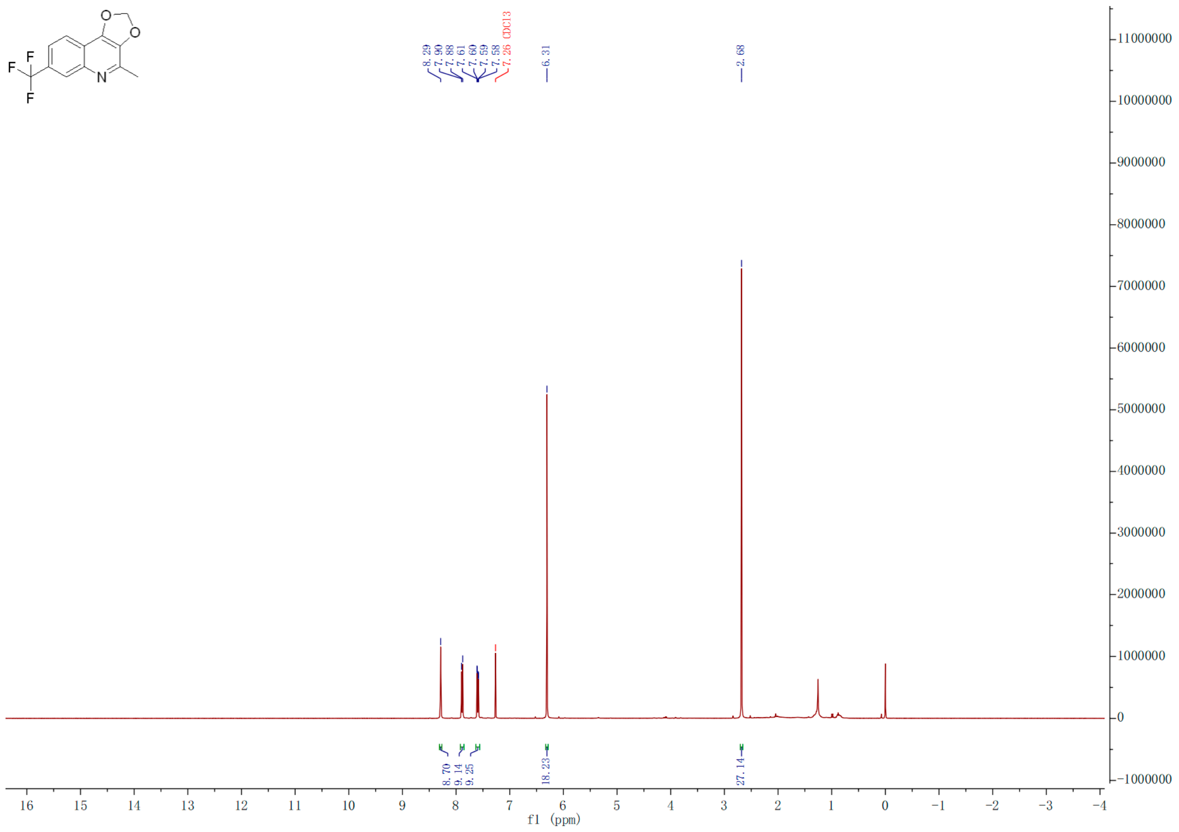

Figure S39. Compound D5 ^{1}H NMR

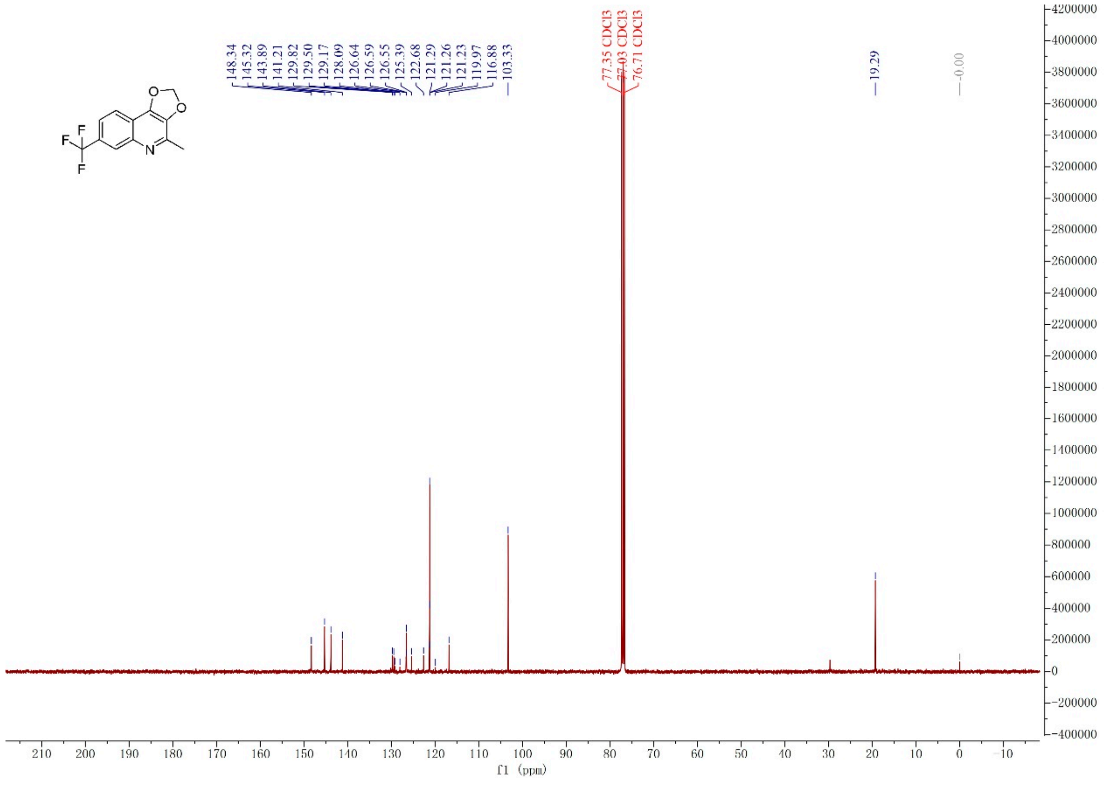

Figure S40. Compound D5 ^{13}C NMR

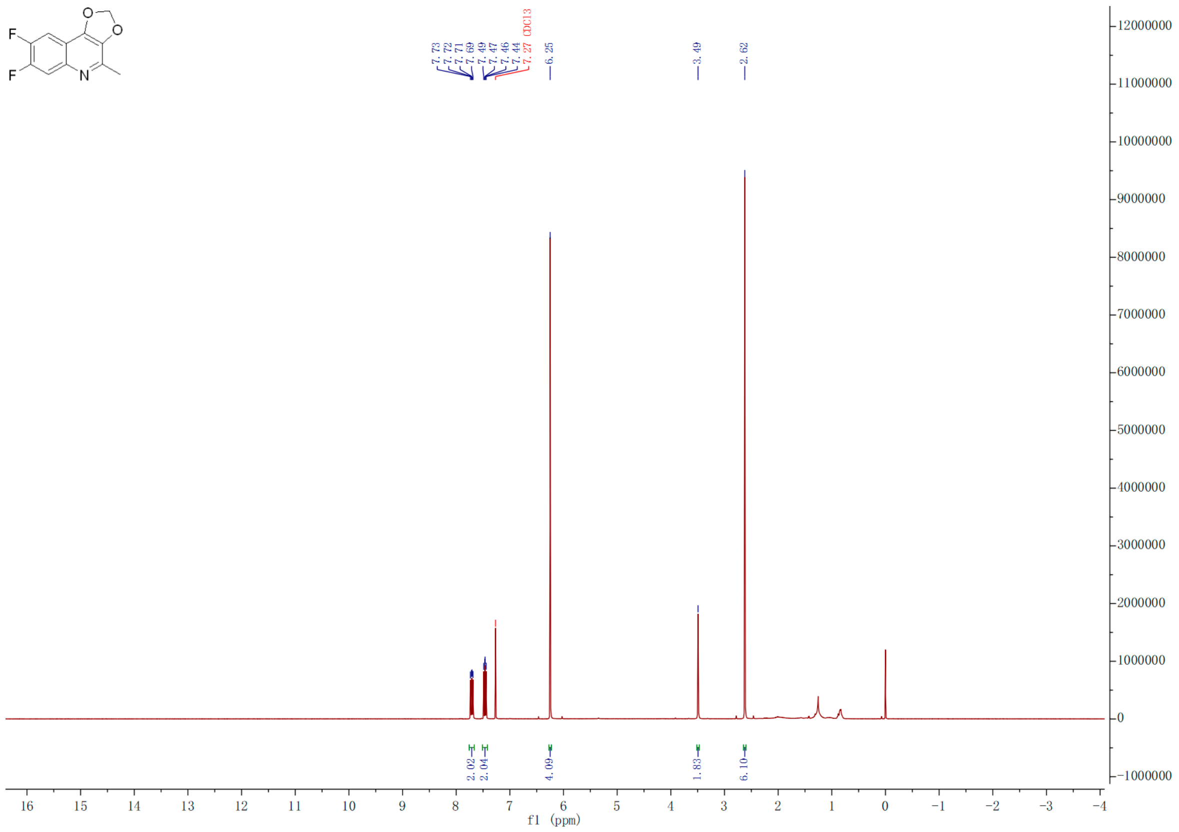

Figure S41. Compound D6 ^{1}H NMR

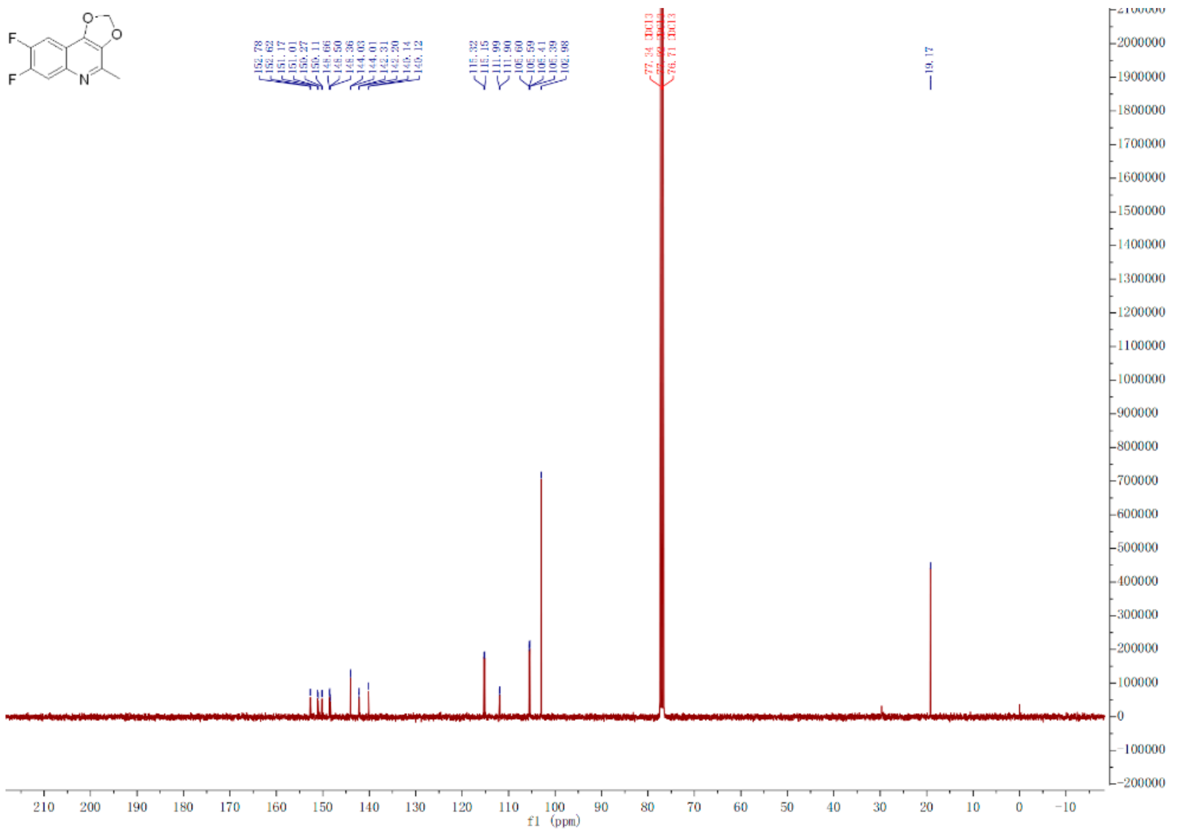

Figure S42. Compound D6 ^{13}C NMR

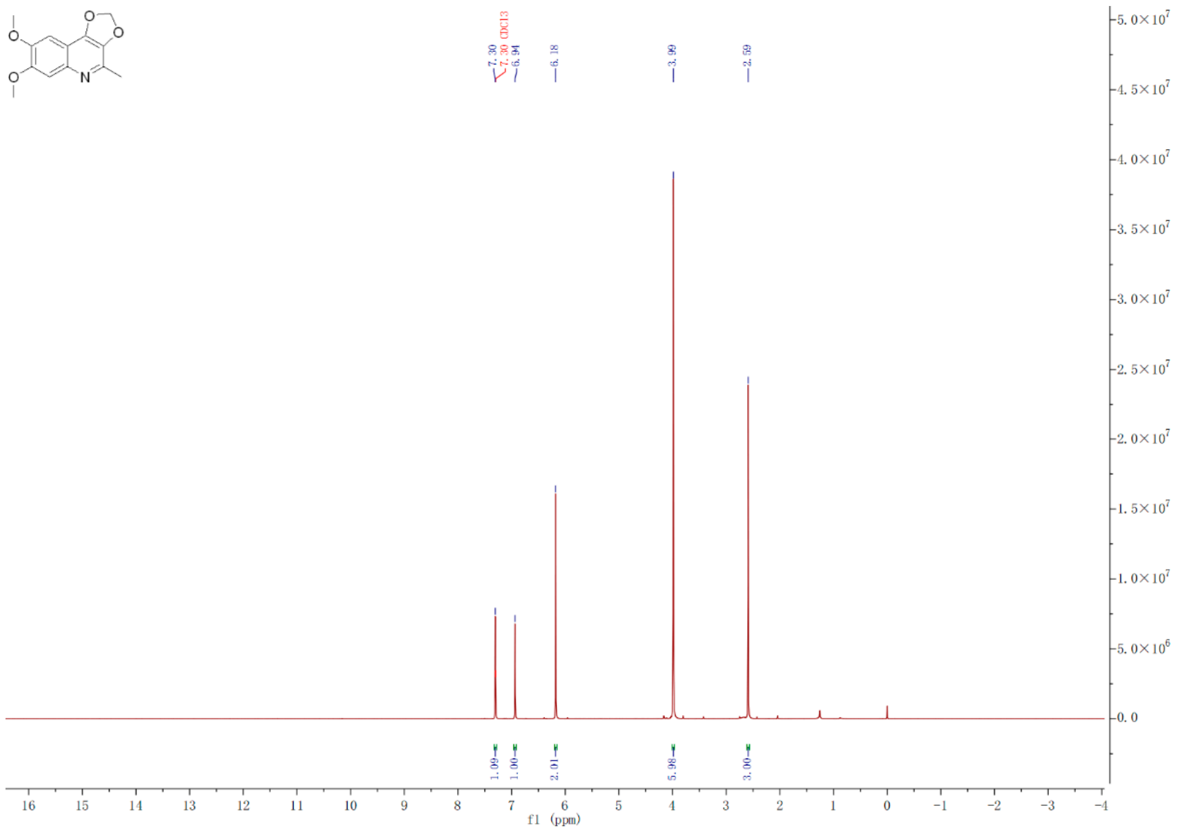

Figure S43. Compound D7 ^{1}H NMR

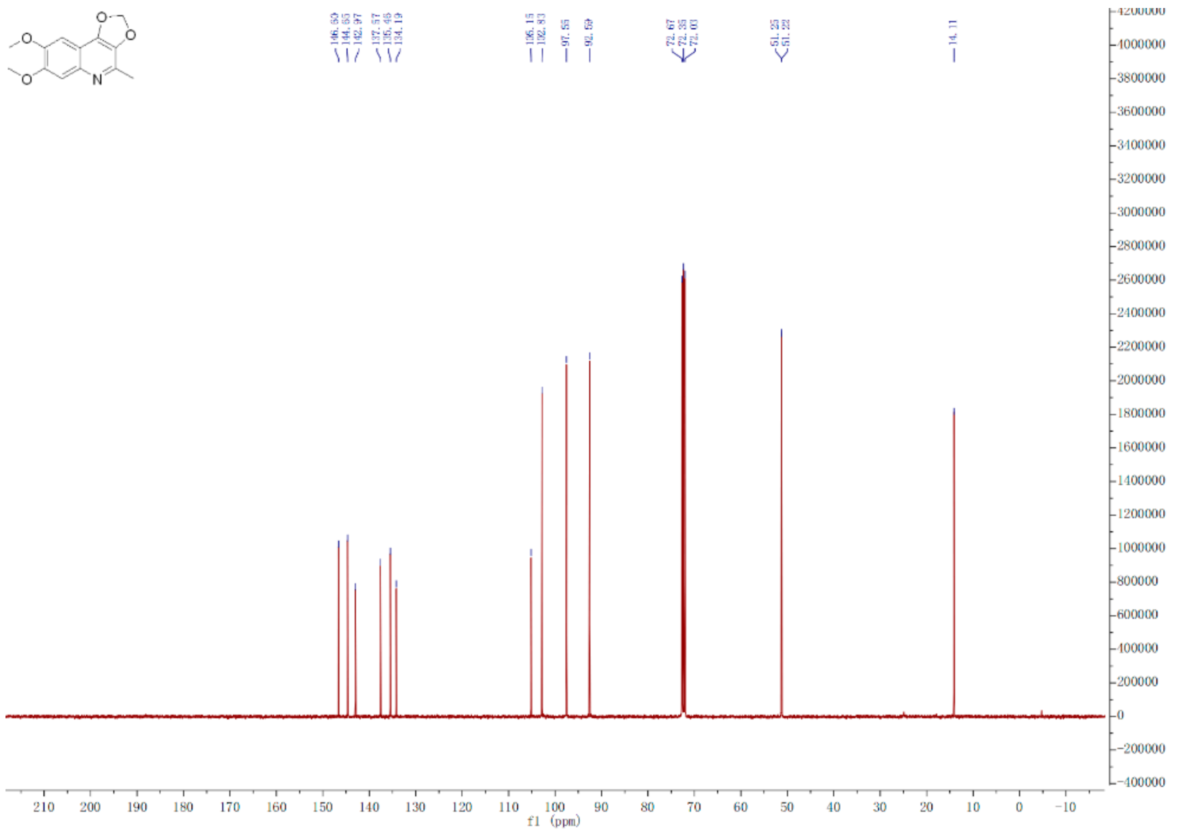

Figure S44. Compound D7 ^{13}C NMR

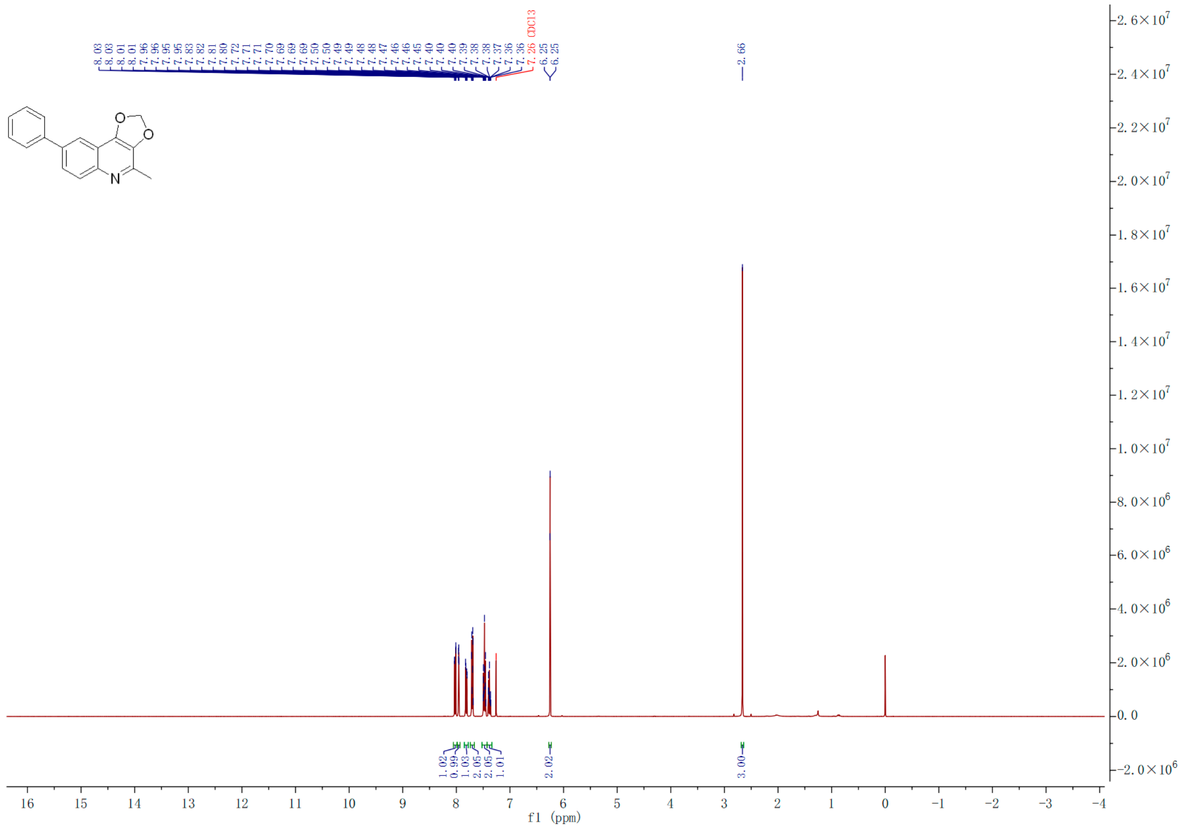

Figure S45. Compound D8 ^{1}H NMR

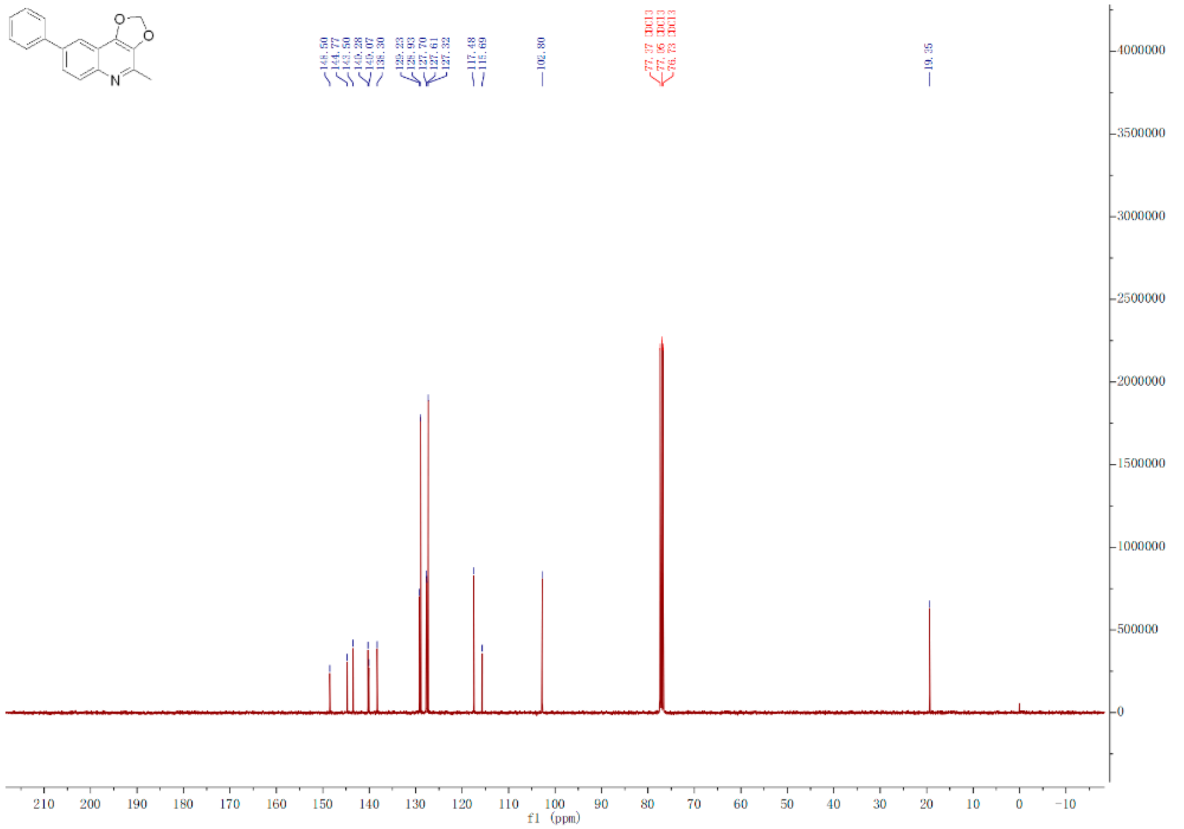

Figure S46. Compound D8 ^{13}C NMR

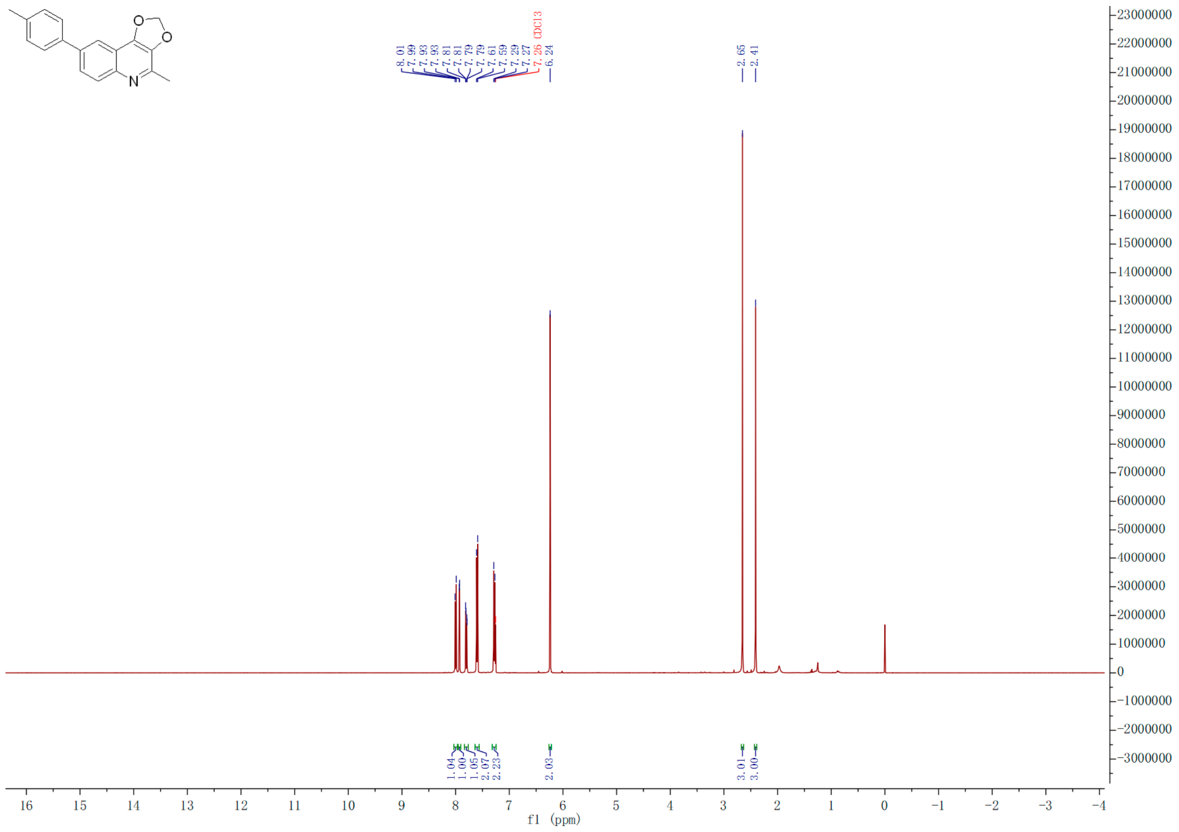

Figure S47. Compound D9 ^{1}H NMR

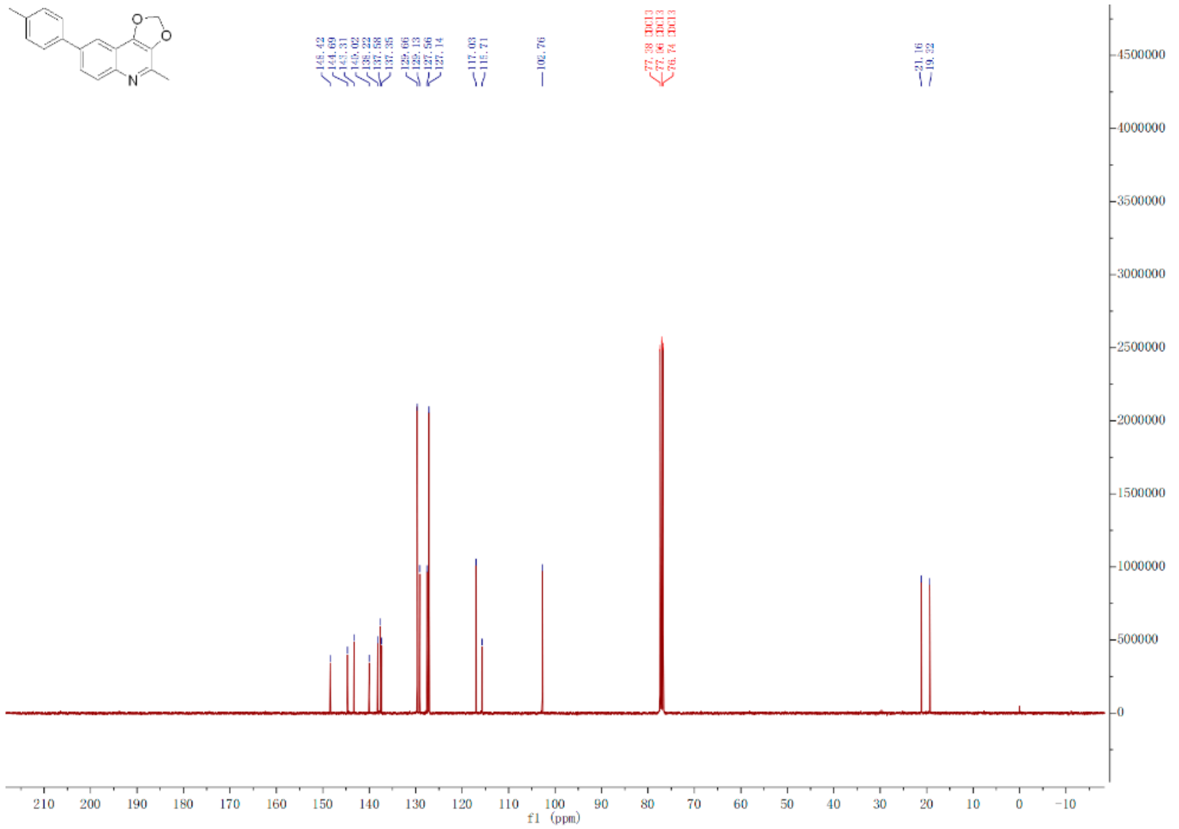

Figure S48. Compound D9 ^{13}C NMR

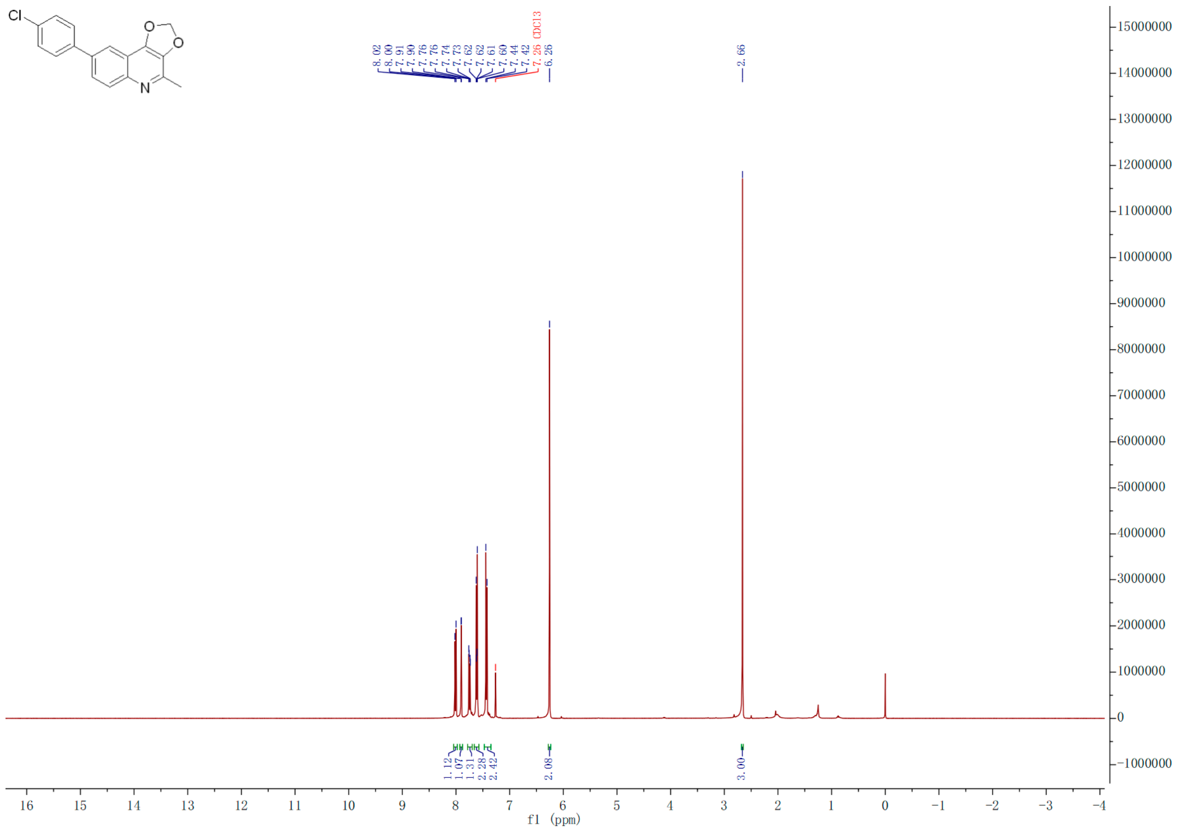

Figure S49. Compound D10 ^{1}H NMR

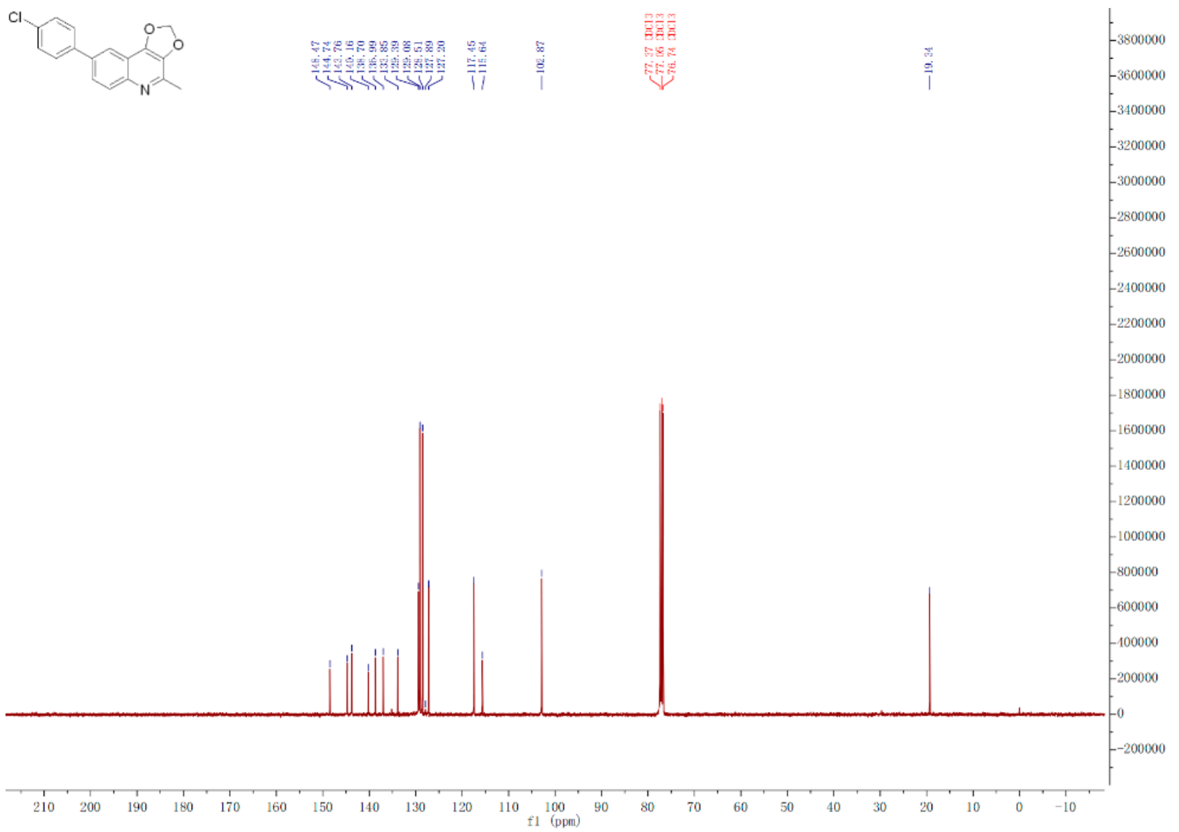

Figure S50. Compound D10 ^{13}C NMR

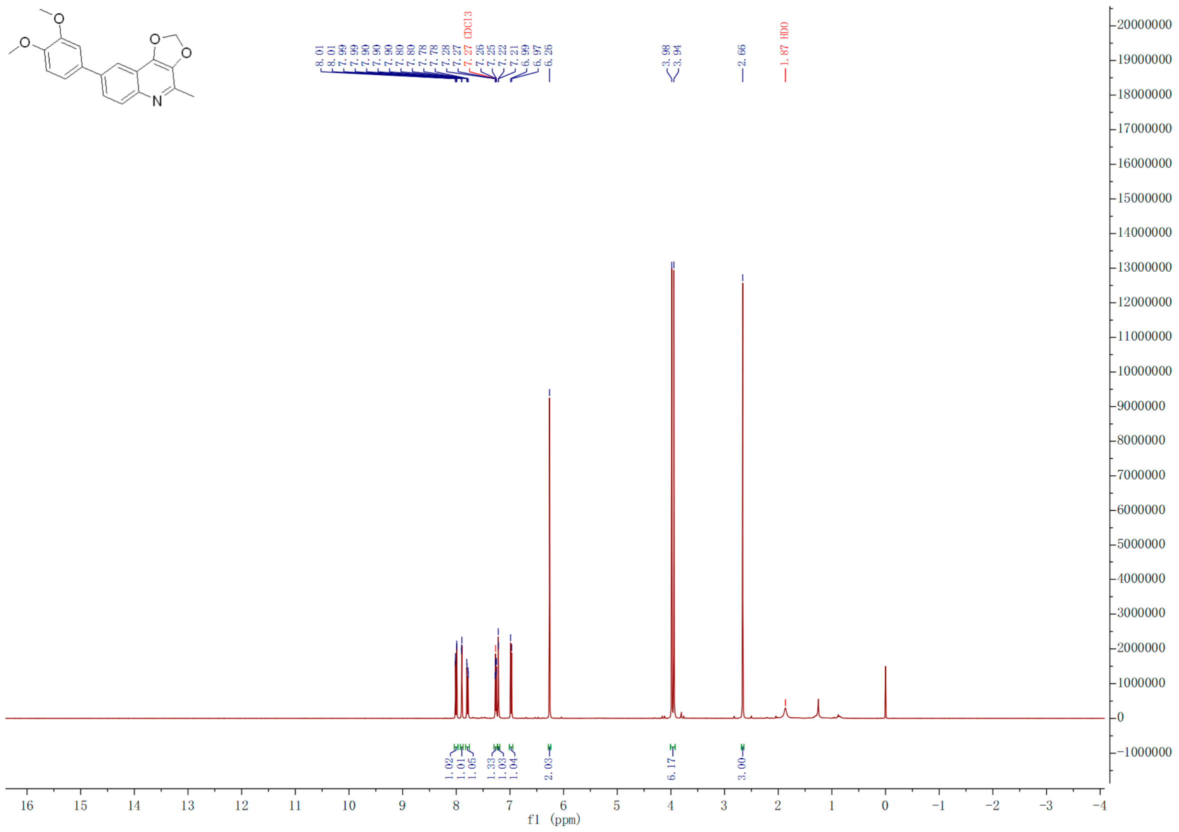

Figure S51. Compound D11 ^{1}H NMR

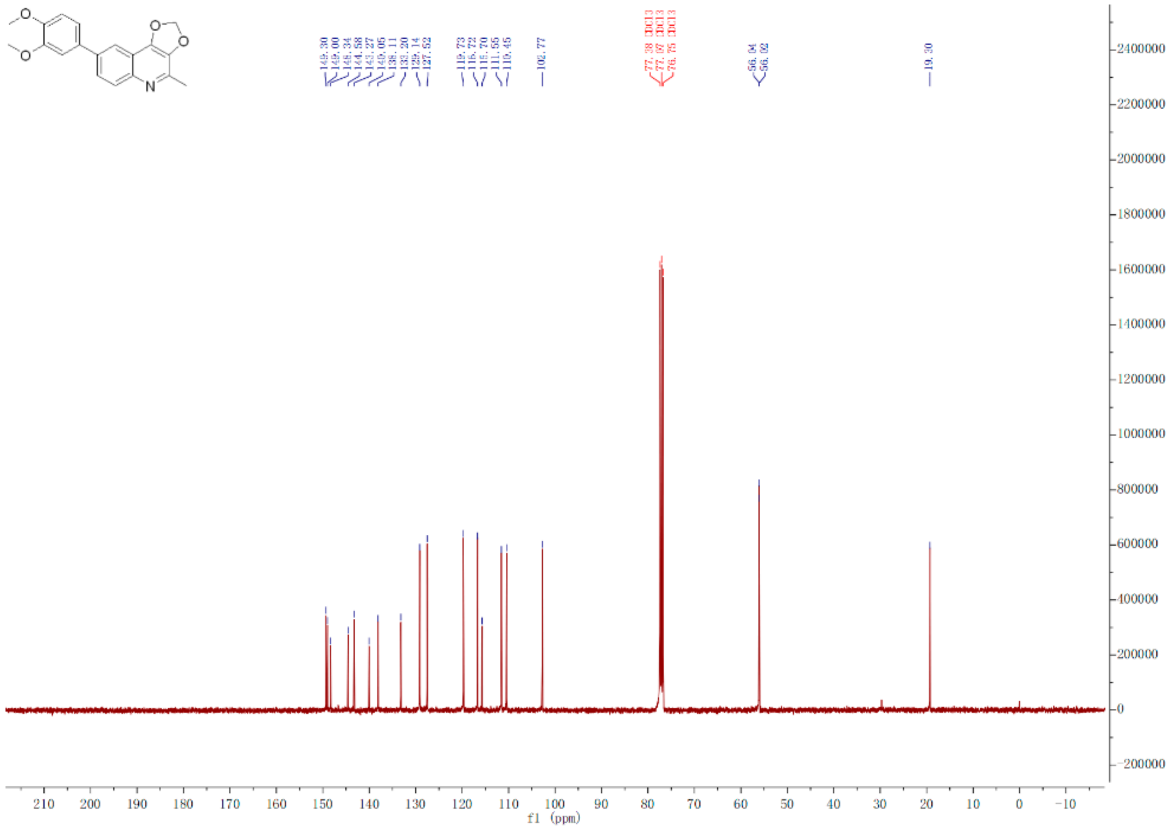

Figure S52. Compound D11 ^{13}C NMR

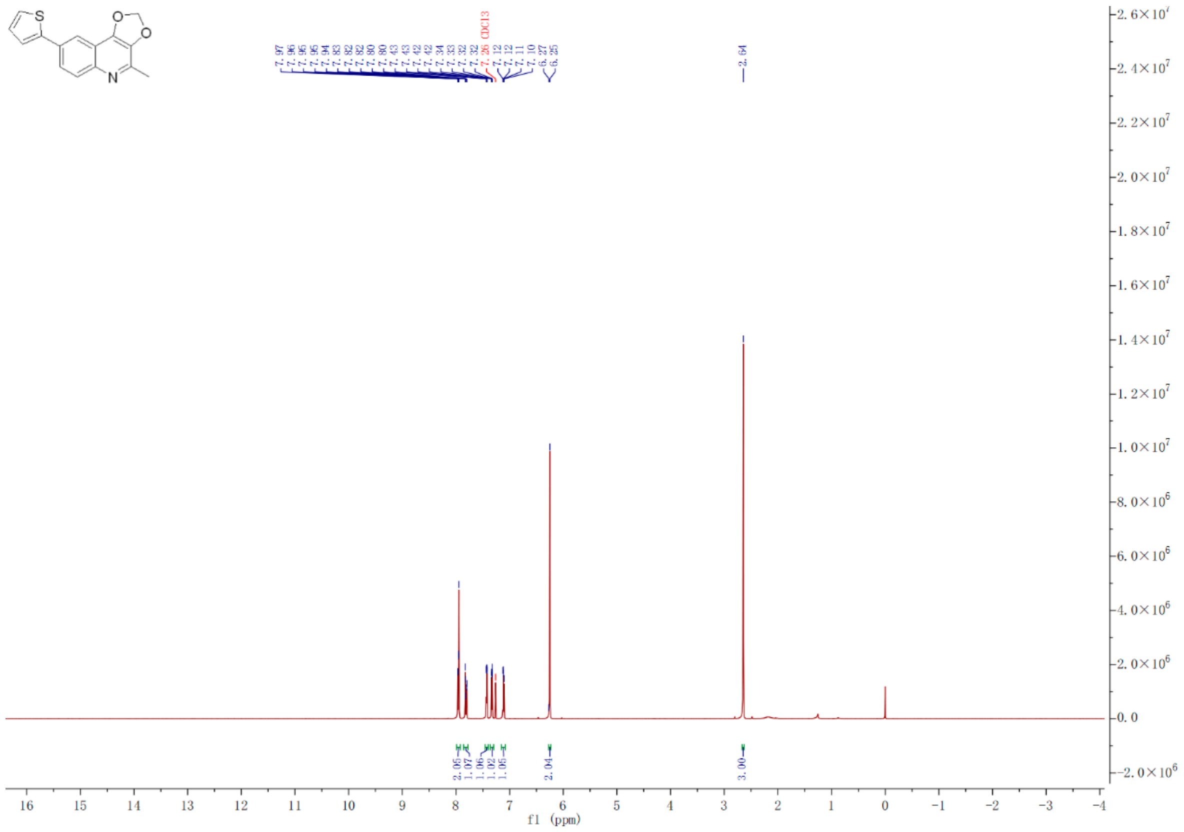

Figure S53. Compound D12 ^{1}H NMR

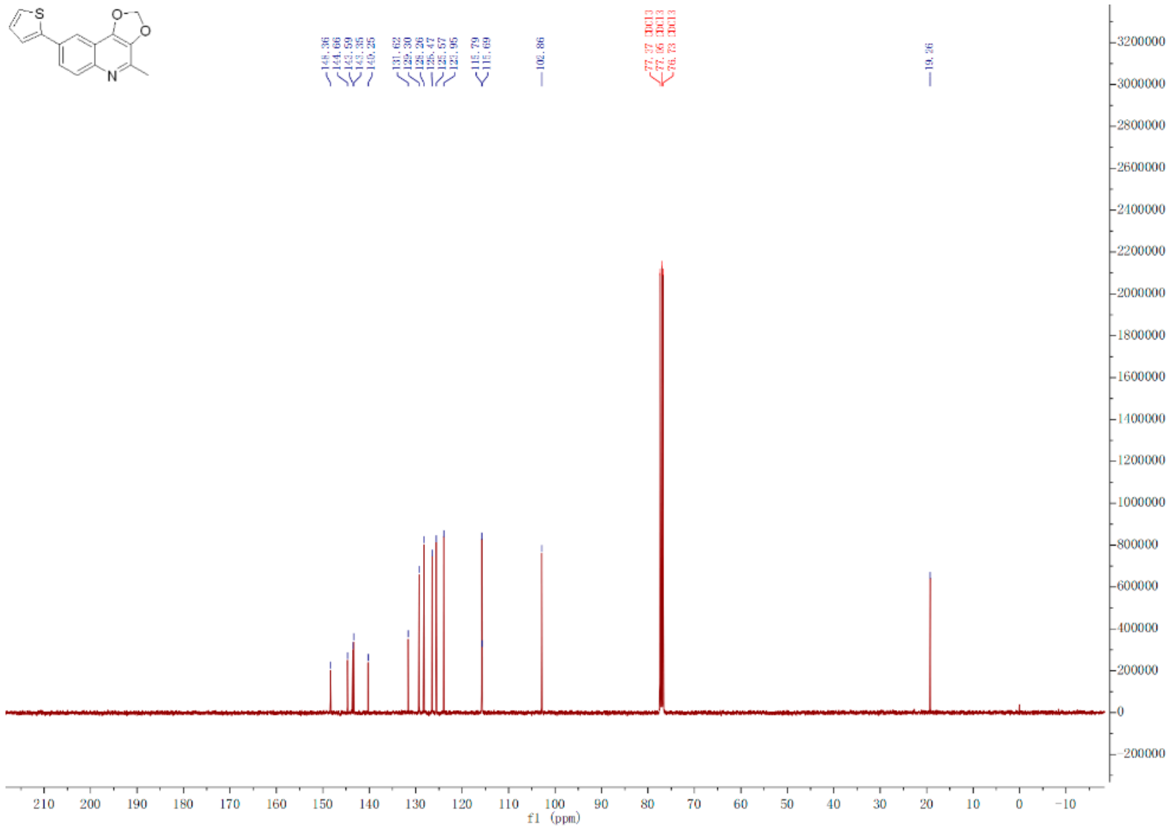

Figure S54 Compound D12 ^{13}C NMR
